# Immune Control of *Burkholderia pseudomallei*––Common, High-Frequency T-Cell Responses to a Broad Repertoire of Immunoprevalent Epitopes

**DOI:** 10.3389/fimmu.2018.00484

**Published:** 2018-03-20

**Authors:** Arnone Nithichanon, Darawan Rinchai, Surachat Buddhisa, Pornpun Saenmuang, Chidchamai Kewcharoenwong, Bianca Kessler, Prasong Khaenam, Ploenchan Chetchotisakd, Bernard Maillere, John Robinson, Catherine J. Reynolds, Rosemary J. Boyton, Daniel M. Altmann, Ganjana Lertmemongkolchai

**Affiliations:** ^1^Centre for Research & Development of Medical Diagnostic Laboratories, Faculty of Associated Medical Sciences, Mekong Health Science Research Institute, Khon Kaen University, Khon Kaen, Thailand; ^2^Department of Medicine, Faculty of Medicine, Khon Kaen University, Khon Kaen, Thailand; ^3^Protein Engineering and Research Department, CEA Saclay, Gif-sur-Yvette, France; ^4^Institute of Cellular Medicine, Newcastle University, Newcastle upon Tyne, United Kingdom; ^5^Department of Medicine, Imperial College London, London, United Kingdom

**Keywords:** *Burkholderia pseudomallei*, epitope, T cell, melioidosis, IFNγ

## Abstract

*Burkholderia pseudomallei* (Bp) is an environmental bacterial pathogen that causes potentially lethal sepsis in susceptible individuals and is considered a Category B, Tier-1 biothreat agent. As such, it is crucial to gain an improved understanding of protective immunity and potential vaccine candidates. The nature of immune correlates dictating why most exposed individuals in endemic regions undergo asymptomatic seroconversion while others succumb to life-threatening sepsis is largely uncharted. Bp seroreactive, immunogenic proteins have previously been identified by antigen microarray. We here set out to conduct an analysis of T-cell recognition of the Bp immunome using serodominant antigens represented in the original antigen microarray, examining immune correlates of disease in healthy seropositive individuals and those with acute disease or in convalescence. By screening a library of 739 overlapping peptides representing the sequences of 20 different Bp antigens, we aimed to define immune correlates of protection at the level of immunoprevalent T-cell epitopes. Responses to a large number of epitopes were common in healthy seropositive individuals: we found remarkably broad responsiveness to Bp epitopes, with 235 of 739 peptides recognized by ≥80% of all tested donors. The cumulative response to Bp epitopes in healthy, seropositive, donors from this endemic region were of the order of thousands of spot forming cells per million cells, making Bp recognition a significant component of the T-cell repertoire. Noteworthy among our findings, analysis revealed 10 highly immunoprevalent T-cell epitopes, able to induce Bp-specific IFNγ responses that were high in responding T-cell frequency within the repertoire, and also common across individuals with different human leukocyte antigen types. Acute melioidosis patients showed poor T-cell responses to the immunoprevalent epitopes, but acquired responsiveness following recovery from infection. Our findings suggest that a large repertoire of CD4 T cells, high in frequency and with broad coverage of antigens and epitopes, is important in controlling Bp infection. This offers an attractive potential strategy for subunit or epitope-based vaccines.

## Introduction

*Bukholderia pseudomallei* (Bp) is a Gram-negative bacterium responsible for melioidosis, which causes sepsis in Southeast Asia, Northern Australia, and other temperate regions (1). Bp is deemed a category B pathogen on the NIAID category A–C pathogen list since there are concerns about the potential for weaponization in a bioterrorism or biowarfare context (2, 3). Although many Bp genomes have now been sequenced, several aspects of pathogenicity and immunology of melioidosis are poorly characterized (3): this is an intracellular pathogen, predominantly infecting antigen presenting cells (APCs), such that some people suffer no clinical symptoms, while others develop sepsis with high mortality. Bp also has the ability to cause recurrent disease several decades after initial exposure. As such, it poses unresolved questions of adaptive immune control. A disease which is challenging to diagnose and which can be rapidly fatal; there is a clear need for improved diagnostics, vaccine strategies and therapies (2, 4).

Reporting of severe and lethal melioidosis cases has been dominated by Thailand, where there have been of the order of 2,000 lethal cases annually. In light of concerns that other countries with evidence of environmental Bp may have substantial underreporting (e.g., through lack of appropriate clinical microbiology), Limmathurotsakul et al. recently presented a comprehensive, evidence-based, predictive map of Bp, estimating global incidence, and mortality due to melioidosis (5). This integrated human and animal melioidosis data with environmental Bp data and environmental covariates proposed to affect the presence of Bp. The model estimates around 165,000 human cases annually, around 90,000 fatal. This suggests that melioidosis cases are underreported from around 45 countries in which it is endemic.

While there have been substantial efforts to identify protective vaccines for melioidosis, there remains a lack of consensus on the most effective vaccine targets and approach (6). The most effective vaccine candidates appear to be live, attenuated variants of Bp, capable of inducing long term protection (7, 8); however, this approach may be precluded by safety concerns (9). An alternative, safer option may be a subunit vaccine encompassing specific antigens or epitopes (10). Peptide epitope subunit vaccine offer advantages in being relatively easy and inexpensive to produce, with the potential to focus immunity on high-frequency effector populations (11). Any discussion of Bp vaccine candidates, whether for global health and/or biodefense applications, must necessarily be informed by an understanding of sequence homology and cross-reactivity with two other, related, *Burkholderia* species. *Burkholderia mallei* (Bm), the causative agent in glanders, is highly pathogenic and considered a biothreat. *Burkholderia thailandensis* (Bt), on the other hand, is non-pathogenic, may confer protective, cross-reactive immunity, and is found in the soil in parts of SE Asia and elsewhere (12).

In endemic regions, where many people will be exposed to Bp but relatively few develop clinical disease, one has a potential opportunity to investigate the nature of protective immunity (13). IFNγ-mediated immunity is likely to be important for the host response: in mice, IFNγ derived from CD4 and CD8 cells, as well as from NK cells, is critical for survival (14–16). We have previously described CD4^+^ and CD8^+^ memory T cells as the source of IFNγ in human immunity to Bp antigens (17). We recently reported epitopes of flagellin (BPSL3319, FliC) and of the alkyl hydroperoxide reductase (BPSL2096, AhpC) of Bp, which induced IFNγ production by human CD4 T cells (18, 19).

Several Bp antigens have been tested for humoral and cellular-mediated immune responses following infection (17, 18, 19, 20, 21). We previously used a Bp protein microarray to identify seroreactive antigens from seropositive individuals and patients who had recovered from melioidosis (13). We suggested that immune responses against the reported proteins may contribute to protection among individuals living in endemic areas, and protect those patients who survive severe Bp infection. In the present study, we aimed to extend the granularity of this previously reported study through comprehensive T-cell epitope mapping, using selected proteins from the same array, in Thai seropositive and clinical disease cohorts, with a view to delineating patterns of T-cell responses at the peptide-MHC (pMHC) level.

## Materials and Methods

### Human Samples

Blood-bank buffy coats were obtained from the Blood Transfusion Center, Khon Kaen Hospital, Khon Kaen, Thailand with ethical approval by Khon Kaen University Ethics Committee for Human Research (Project no. HE470506). Healthy Bp-seropositive buffy coat samples were used on the basis of displaying IHA titers of 1:40 or greater whereas seronegative samples were <1:40 (22, 23).

Melioidosis patients (defined as Bp culture-positive and displaying clinical signs and symptoms of infection) were recruited at Srinagarind Hospital, Khon Kaen University, Khon Kaen, Thailand. Patients receiving immunosuppressive therapy, with known kidney disease, or HIV-infected, were excluded from the study. “Recovered melioidosis” was defined as those individuals who had recovered from melioidosis previously diagnosed by isolation of Bp from blood or tissues, completed antibiotic treatment, and clinically well. All patients gave informed, written consent before blood donation. Peripheral mononuclear cells (PBMCs) from donors were isolated by Ficoll-Hypaque (Sigma-Aldrich) density gradient centrifugation, and stored at −80^o^C until used.

### Peptide Libraries

Synthetic peptide libraries were used, covering the full length of 20 candidate Bp proteins as shown in Table 1. Sets of 20-mer peptides, overlapping by 10 amino acids, were synthesized by GL Biochem (Shanghai, China), based on the sequence of Bp K96243 strain. We note that 20 mers are larger than most MHCII presented epitopes and that, thus, the peptides may have been reprocessed and, indeed, that some peptides may have carried more than one epitope. Fine-mapping of minimal epitopes within each peptide was beyond the scope of this study. Peptides were stored and shipped as stock solutions of 25 mg/mL in DMSO (i.e., 1,000×). We have previously confirmed baseline values for the addition to PBMC of diluted DMSO in medium or of negative control peptides.

**Table 1:** Listing and details of twenty *Burkholderia pseudomallei* candidate antigens for T-cell epitope mapping studies

Locus tag	Protein name	Number of amino acids	Number of overlapping peptides	Molecular weight (kDa)	Subcellular localization	Number of tested samples	Number of analyzed samples
BPSL0280	Flagellar hook-associated protein (FlgK)	667	67	67.2	Extracellular (flagella)	55	30
BPSL0919	4-hydroxy-3-methylbut-2-enyl diphosphate reductase	326	33	35.2	Cytoplasmic	50	34
BPSL0999	OmpA family transmembrane protein	215	22	21.4	Outer Membrane	53	47
BPSL1445	Putative lipoprotein	195	18	19.6	Extracellular	45	35
BPSL2096	Hydroperoxide reductase	182	17	20.3	Periplasmic	50	34
BPSL2504	Hydrolase	280	27	30.1	Cytoplasmic	55	37
BPSL2520	Putative exported protein BPSL2520	198	19	21.3	Unknown	45	30
BPSL2522	Outer membrane protein A	224	23	24.1	Outer Membrane	50	35
BPSL2697	Chaperonin (GroEL)	546	54	57	Extracellular	44	23
BPSL2765	Putative OmpA family lipoprotein	170	16	18.6	Surface	46	16
BPSL3319	Flagellin (FliC)	388	38	39.3	Flagellar	51	45
BPSS0477	60-kDa chaperonin (GroEL2)	546	54	56.8	Cytoplasmic	45	35
BPSS0530	Hypothetical protein BPSS0530	453	44	49.9	Cytoplasmic	52	43
BPSS1385	ATP/GTP binding protein	328	32	35.8	Cytoplasmic	51	45
BPSS1492	Burkholderia intracellular motility A (BimA)	516	50	51.7	Outer Membrane	41	25
BPSS1525	Guanine nucleotide exchange factor BopE	261	25	28.7	Extracellular	52	39
BPSS1531	Effector protein (BipC)	419	41	44.3	Periplasmic	50	35
BPSS1532	Cell invasion protein (BipB)	620	61	64.6	Cytoplasmic	45	34
BPSS1599	Type IV pilus biosynthesis protein (PilO)	432	44	47.9	Unknown	52	47
BPSS2141	Periplasmic oligopeptide-binding protein precursor (OppA)	554	54	61.8	Periplasmic	45	30

Total number of peptides			739				

### Preparation of Paraformaldehyde (PFA) Fixed Bp

Live clinical isolate Bp from Thai strain K96243 (24) was grown to mid-log phase at 37°C in Luria–Bertani (LB) broth for 18 h. Bacteria were washed twice with PBS, then separated into two sets for enumeration of number of bacteria by plating on LB agar and for PFA fixation with 2% PFA for 1 h at room temperature before being stored at −80^o^C until use. All manipulation of live Bp was carried out at laboratory biosafety level 3 (BSL3) under US Centers for Disease Control (CDC) regulations for research.

### Human T-Cell Epitope Mapping

*Burkholderia pseudomallei* T-cell epitope mapping by assay of responses to individual peptides in IFNγ ELISpot (C.T.L., USA) was as previously described (19). In brief, precoated, 96-well polyvinylidene difluoride (PVDF) plates (MSIP, Millipore) were cultured overnight with 15-µg/mL IFNγ antibody, then 2.5 million/mL of isolated PBMC, pulsed with killed, intact Bp as a positive control, with each peptide at 25 µg/mL, or with culture medium as negative control, were added in triplicate, and cocultured for 48 h. While we note that CD8 T cells can respond to optimal peptide concentrations well below 25 µg/mL, we have previously identified this as the optimal concentration for large-scale, high-throughput, CD4 screening of peptide libraries, without risk of losing responses to low avidity epitopes. IFNγ secretion was detected by addition of antihuman-IFNγ detection antibody solution at room temperature for 3 h. After washing, streptavidin-alkaline phosphatase solution was added to the plate for 30 min, and spots were revealed with developer solution for 10 min. IFNγ spots were quantified using an Immunospot analyzer (C.T.L.). Results were calculated as spot forming cells (SFC)/million PBMC.

### Human Leukocyte Antigen (HLA) Typing

Donor HLA-DRB1 genotypes were analyzed using PCR sequence-specific primer (PCR-SSP), using 20 primer pairs (25). Primer mixtures comprised a pair of allele-specific primers and a pair of 256-bp internal control primers.

### HLA Binding Assay

Human leukocyte antigen-peptide binding across a panel of HLA class-II alleles was as previously described (18). Purified HLA-DR heterodimers derived from the relevant EBV B-lymphoblastoid cell lines were assayed for peptide binding by competitive ELISA (26). HLA-DR molecules were diluted with a known, selected, binder, biotinylated peptide, and serial dilutions of competitor peptides. Unlabeled forms of the biotinylated peptides were included as reference peptides, allowing us to assess the validity of each experiment. After 24–72 h incubation at 37°C, samples were neutralized. They were then applied to 96-well Maxisorp ELISA plates (Nunc, Denmark) which had been previously coated with the HLA-DRα mAb, L243, used at 10 µg/mL. Samples were bound to antibody-coated plates for 2 h at room temperature. The extent of binding by biotinylated peptide was then detected by incubation with streptavidin-alkaline phosphatase conjugate (GE-healthcare, Saclay, France), and after washing, by addition of 4-methylumbelliferyl phosphate substrate (Sigma, France). Fluorescence was assayed at 450 nm, with excitation at 365 nm. The peptide concentration that prevented binding of 50% of the labeled peptide (IC_50_) was evaluated. Data are expressed as relative affinity, as a ratio of the IC_50_ of test peptide to the IC_50_ of a reference peptide chosen as a high binder to the corresponding HLA-DR heterodimer. Mean + SEM were calculated from three independent experiments and relative affinities of <20 were considered high binders and relative affinities of 20–100, moderate binders.

### Statistical Analysis

Statistical analysis was performed using Graphpad Prism version 6 software (GraphPad). The Mann–Whitney *U* test was applied for comparing among independent sample groups. The Wilcoxon signed rank test was applied for comparing dependent sample groups, *P*-value <0.05 was considered statistically significant (*P < 0.05, **P < 0.01, and ***P < 0.001).

## Results

### Immunoprevalent T-Cell Epitopes Recognized by Seropositive Healthy Donors

**Figure 1:**
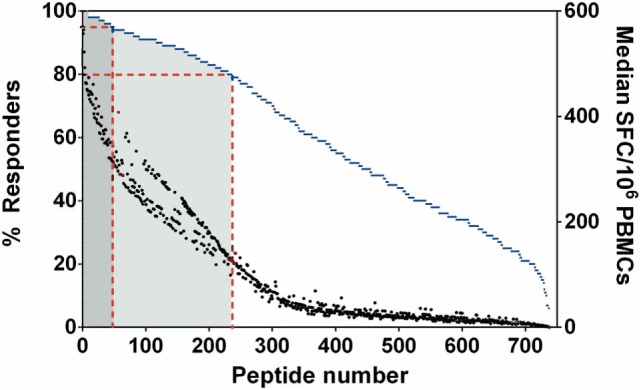
Immunoprevalent peptides identified using *Burkholderia pseudomallei* antigen peptide libraries in seropositive healthy donors. Peptides were ranked on the basis of % responders from highest to lowest, plotted on the left *y*-axis (blue dashed line represents % responders = total number of individuals that responded to a particular peptide/number of donors tested). Median spot forming cell (SFC)/10^6^ peripheral mononuclear cell (PBMC) is plotted on the right *y*-axis (black dot represented for median SFC/10^6^ PBMCs = sum of total SFC/number of donors tested). Red dashed lines indicate the 49 peptides which were recognized by ≥95% of all tested donors (dark gray zone), the 235 peptides which were recognized by ≥80% of all tested donors (light gray zone).

We initially addressed human T-cell epitope identification using overlapping peptide libraries (27, 28). Protein sequences of 20 key, serodominant, Bp proteins (13), previously reported by our group, were retrieved from the NCBI protein database. Peptide libraries were synthesized for each candidate as 20 mers with an overlap of 10. The details of peptide libraries are shown in Table 1 and the strategy for identification of T-cell epitopes is shown in supplementary data (Figure S1 in Supplementary Material). We screened for induction of T-cell IFNγ responses against each peptide, totaling 739 peptides from 20 Bp antigens; for this we used PBMC from healthy, seropositive donors from an endemic area. Demographic data and HLA frequency distribution are shown in Table S1 in Supplementary Material. Based on our demographic data, the HLA distribution of our selected cohort (*N* = 129) is concordant with previous reports from the Khon Kaen region of Thailand (25). In each case, PFA fixed, whole Bp (“Fixed Bp”) was used as a positive control. Any non/low-responder samples showing an IFNγ response to Fixed Bp lower than medium-only +2 SD cutoff were excluded (Figure S2 in Supplementary Material). The results from one representative seropositive donor are shown in supplementary data (Figure S3 in Supplementary Material). The percent of responders was defined as the number of individuals that responded to a peptide divided by the total number of individuals tested, multiplied by 100. This gives an indication of how common or rare a response to a peptide is in the population of Bp seropositive individuals tested. The frequency of the T-cell response to a given peptide was taken as the median number of IFNγ-producing cells calculated from all donors tested (SFC/10^6^ PBMC). This gives an indication of the magnitude of the T-cell IFNγ response. Peptide responses were then ranked on the basis of the percentage of responders and magnitude of response to assess relative immunoprevalence. From this analysis, there were 49 peptides that carried T-cell epitopes recognized by ≥95% of all tested seropositive donors, and 235 peptides recognized by ≥80% of all tested donors (Figure 1). The list of the top 235 commonly recognized peptides is shown in Table S2 in Supplementary Material. Positive peptide responses tended to be of relatively high frequency at around 200–400 SFC/10^6^ cells, and each seropositive donor tended to respond to multiple epitopes, both within and across different Bp antigens. Thus, the cumulative response to Bp in healthy seropositive donors from an endemic region is, generally, of the order of some thousands of SFC/10^6^ cells, making Bp antigen recognition a significant component of the T-cell repertoire.

### HLA Class-II Polymorphism and Patterns of Response to Strongly Immunoprevalent Bp Epitopes

The observation that a large number of Bp epitopes could be recognized at high frequency by CD4 T cells from this Thai, seropositive, donor cohort led us to interrogate in more detail the possibility that the most commonly recognized epitopes must have the ability to bind multiple HLA class-II products for presentation (29). We selected 10 of the most commonly recognized immunoprevalent peptides (recognized by ≥80% of all tested donors) from the top 235 peptides (Table 2). The selected peptides were analyzed for binding to HLA class-II heterodimers, including the common SE Asian polymorphisms, HLA DRB1*04, 07, 09, 11:01, 12:02, 15:01, and 15:02 (25). HLA binding affinities were then related to our findings from IFNγ ELISpots (Table 3). We found that the selected 10 immunoprevalent peptides showed broad HLA class-II binding. HLA-DRB1*15:01 was able to bind selected immunoprevalent peptides with high affinities, while the closely related variant from the Thai population, HLA-DRB1*15:02, bound most of the peptides relatively poorly. We note that these two alleles of HLA-DR15 are found in roughly equal proportions in Thai population (www.allelefrequencies.net). The heterodimers HLA DRB1*04, 07, 09, 11:01 and 12:02 tended to bind the immunoprevalent peptides with moderate affinity. Some of the immunoprevalent peptides such as p6 from hydroperoxide reductase (BPSL2096) and p18 from ATP/GTP binding protein (BPSL1385) had the property of binding to virtually all the tested HLA class-II heterodimers at moderate to high affinity.

**Table 2:** Selected 10 T-cell immunoprevalent peptides of *Burkholderia pseudomallei* (Bp) identified from seropositive healthy donors using 20 Bp libraries.

Locus tag	Peptide number	Location of peptide on protein	Peptide sequence	Region feature	% Responders
BPSL0999	P14	131–150	NQNPQITASVVGYTDSTGSA	OmpA family transmembrane protein	96
BPSL0999	P18	171–190	RGVAANRLSAQGMGASNPIA	OmpA family transmembrane protein	94
BPSL2096	P06	51–70	KDFTFVCPTEIVEFAKLAKQ	Hydroperoxide reductase	82
BPSL2522	P12	111–130	PAGKQKLDELAAKIQGMNVE	Outer membrane protein A	97
BPSL2522	P13	121–140	AAKIQGMNVEVVVATGYTDR	Outer membrane protein A	100
BPSL2522	P14	131–150	VVVATGYTDRIGSDKYNDRL	Outer membrane protein A	100
BPSS1385	P11	101–120	SNRVMWNDRYDTLLIARDPR	ATP/GTP binding protein	98
BPSS1385	P12	131–150	TDFGGLENYKELTGGADPFA	ATP/GTP binding protein	93
BPSS1385	P18	171–190	DVPIDPTSIEYLENTSFAEH	ATP/GTP binding protein	100
BPSS1531	P12	111–130	HDALVQRHVSLDGAKAAHGE	Effector protein BipC	89

**Table 3:** Analysis of 10 immunoprevalent epitopes with human leukocyte antigen (HLA) class II (DRB1) immunogenicity with respect to ELIspot response and HLA binding.

Peptide	DRB1*04		DRB1*07		DRB1*09		DRB1* 11:01		DRB1* 12:02		DRB1* 15:01		DRB1* 15:02	
	Elispot^a^	HLA binding^b^	Elispot^a^	HLA binding^b^	Elispot^a^	HLA binding^b^	Elispot^a^	HLA binding^b^	Elispot^a^	HLA binding^b^	Elispot^a^	HLA binding^b^	Elispot^a^	HLA binding^b^
BPSL0999 P14	**583**	**33**	**460**	**30.2**	**567**	**37**	**588**	189	**489**	>136	**417**	**1**	**601**	542
BPSL0999 P18	**617**	**82**	**487**	**11**	**634**	367	**616**	**72**	**441**	**14**	**415**	157	**456**	>3 333
BPSL2096 P06	**357**	**9**	**460**	**0.3**	**378**	**18**	**180**	**43**	**351**	**25**	**347**	**7**	**424**	222
BPSL2522 P12	**325**	>145	**531**	333	**541**	>689	**266**	**77**	**410**	**40**	**457**	>270	**471**	>3 333
BPSL2522 P13	**362**	nd	**487**	**11**	**578**	**42**	**351**	**23**	**534**	**5**	**488**	**5**	**487**	667
BPSL2522 P14	**383**	>145	**460**	>336	**593**	>689	**244**	>278	**461**	>136	**463**	**10**	**492**	1 826
BPSS1385 P11	**664**	**26**	**372**	**85**	**604**	556	**470**	**95**	**537**	112	**373**	**3**	**651**	213
BPSS1385 P12	**837**	107	**383**	**22**	**590**	**28**	**488**	**90**	**608**	**42**	**501**	**22**	**903**	>3 333
BPSS1385 P18	**691**	**53**	**376**	**30**	**376**	**66**	**376**	**58**	**466**	**4**	**370**	**4**	**611**	707
BPSS1531 P12	**555**	**74**	**474**	**28**	**378**	167	**325**	113	**377**	208	**501**	**36**	**367**	760

*^a^Median SFC/10^6^ PBMCs of HLA donors*.

*^b^Strength of relative HLA binding affinity; affinities of <20 were considered to be high binders (red) while 20–100 were moderate binders (green)*.

### Comparative Responses to Immunoprevalent Peptides in Healthy Seropositive Donors and in Patients during Acute Melioidosis and following Recovery

**Figure 2:**
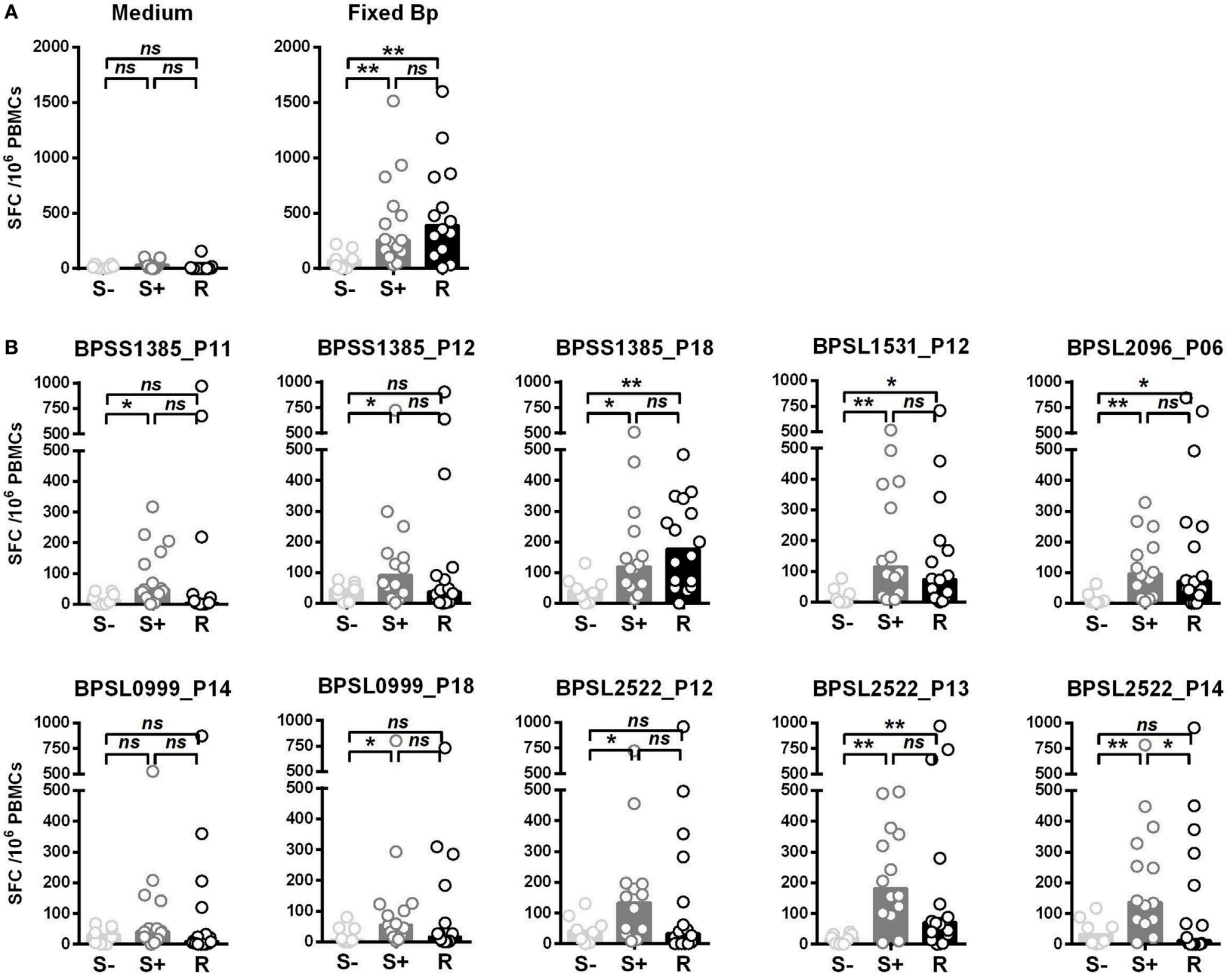
Differential IFNγ responses to 10 *Burkholderia pseudomallei* (Bp) immunoprevalent peptides in individuals that are seronegative for Bp, seropositive for Bp, or have recovered from acute melioidosis. Peripheral mononuclear cells (PBMCs) from seronegative (S−, *n* = 8), seropositive (S+ , *n* = 15), and individuals that have recovered from melioidosis (R, *n* = 15) were cultured with medium only control or paraformaldehyde (PFA) fixed Bp **(A)**, or the top 10 immunoprevalent peptides **(B)**. Horizontal lines represent mean value for each group. Statistical significance was determined using Mann–Whitney *U* test; ns, not significant, * *p* < 0.05 and ** *p* < 0.01.

We then compared T-cell responses to the set of the 10 most strongly immunoprevalent peptides in additional cohorts of Thai seronegative (S−), seropositive (S+), and recovered (R) melioidosis patients. The key question was whether there were features of T-cell epitope recognition differentiating between asymptomatic seropositives and patients who had recovered from acute, clinical disease (Figure 2). T-cell responses to Fixed Bp were similarly raised in the seropositive and recovered melioidosis sample sets relative to the seronegative group. These results are consistent with a previous report from our group (17) (Figure 2A). For most of the epitope responses tested, T-cell recognition was similar between the seropositive group and the recovered melioidosis group (Figure 2B). However, the response to the OmpA BPSL2522 p14 epitope was lower in recovered patients than in seropositive healthy donors. This raises the possibility that OmpA may be poorly expressed by Bp or poorly processed/presented to the immune system during acute melioidosis.

**Figure 3:**
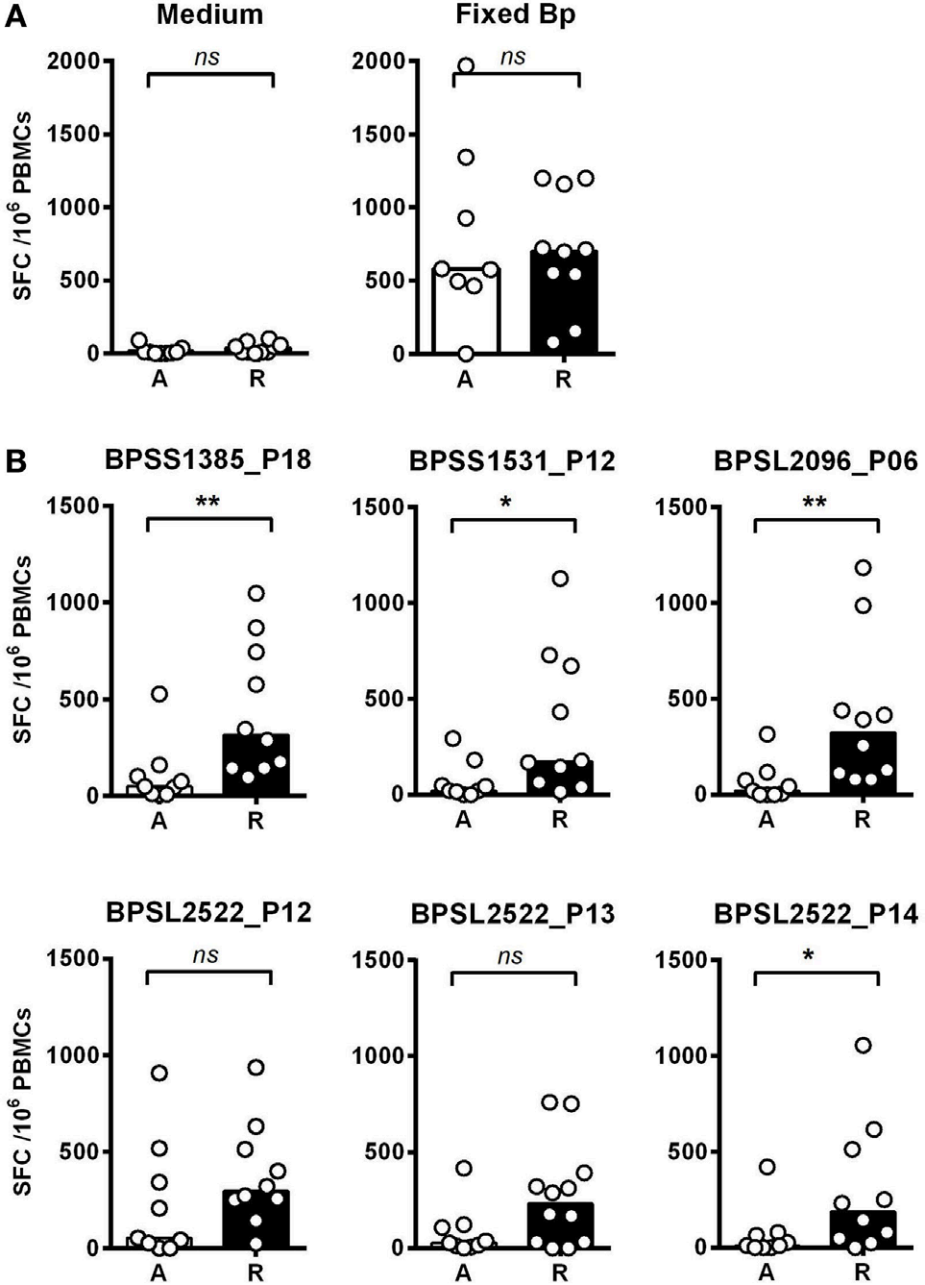
IFNγ responses to *Burkholderia pseudomallei* (Bp) and T-cell immunoprevalent peptides in patients with acute septicemic melioidosis compared with recovered individuals. IFNγ response to the top six immunoprevalent peptides of Bp measured by ELISpot analysis of peripheral mononuclear cell (PBMC) taken from patients with a diagnosis of acute melioidosis (A, *n* = 9) and melioidosis recovered individuals (R, *n* = 10). **(A)** shows the results for medium only control and paraformaldehyde (PFA) fixed Bp. **(B)** shows the results for six immunoprevalent peptides. Statistical significance was determined using Mann–Whitney *U* test; ns, not significant, * *p* < 0.05 and ** *p* < 0.01.

**Figure 4:**
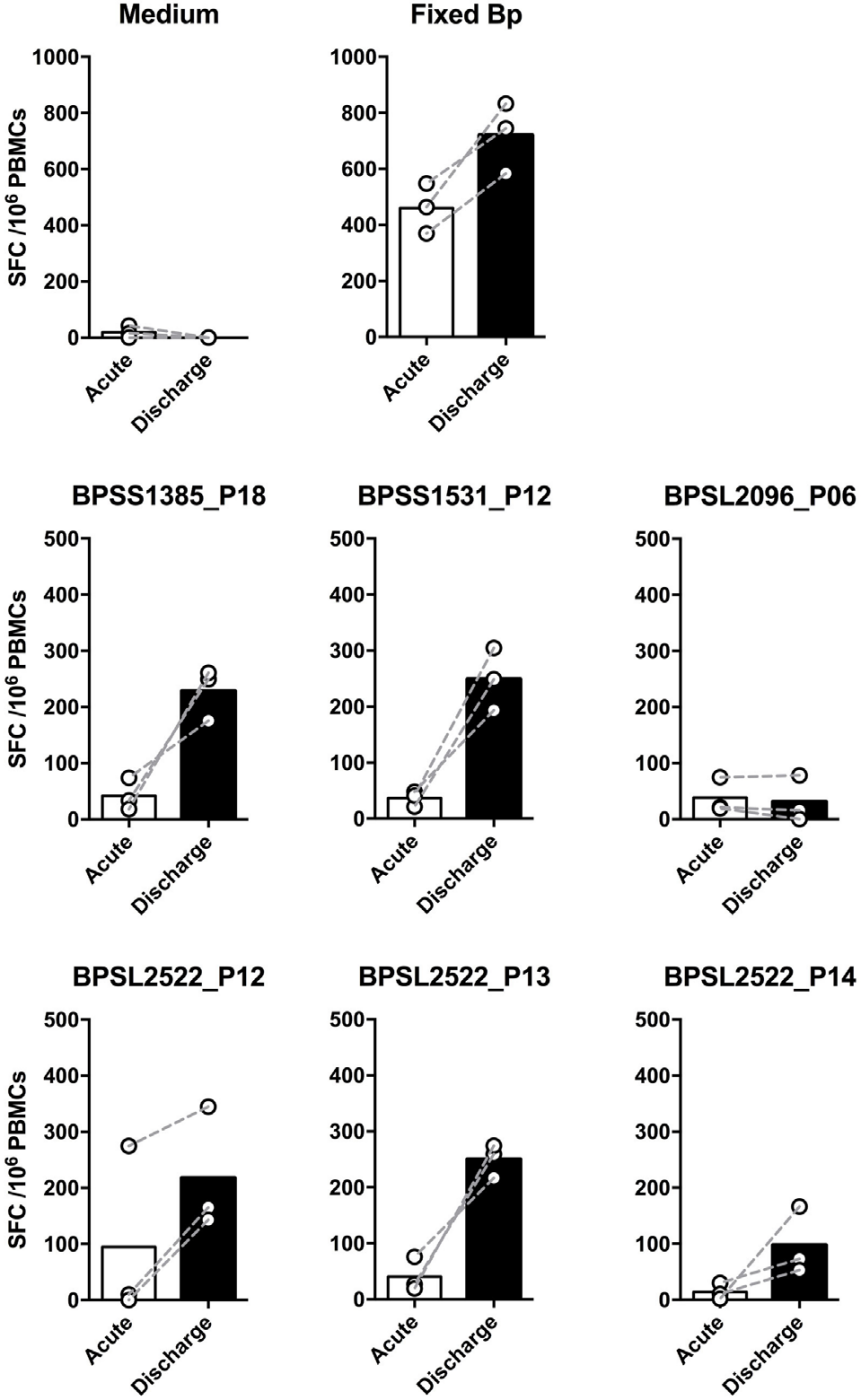
Comparison of IFNγ responses to top six T-cell immunoprevalent peptides of *Burkholderia pseudomallei* (Bp) in patients with a diagnosis of acute melioidosis at the time of acute admission and following recovery having completed antibiotic treatment. Peripheral mononuclear cells (PBMCs) from patients diagnosed with acute melioidosis were cultured with medium only control, paraformaldehyde (PFA) fixed Bp, or T-cell immunoprevalent peptides for 48 h. IFNγ induction was quantified by ELISpot. The results show the comparison of individual IFNγ responses between samples taken on admission (acute, white bar) and discharge (day 30, black bar) from the same patients (*n* = 3). The differences were not statistically significant using Wilcoxon matched-pairs signed rank test.

We then investigated T-cell epitope recognition in acute septicemic melioidosis patients compared with convalescent patients. The magnitude of T-cell response to Fixed Bp was not different between groups, arguing against a global shutdown of the specific response to bacterial infection during acute sepsis (Figure 3A). However, responses to the immunoprevalent epitopes tended to be lower during acute disease than in recovered melioidosis patients, reaching significance in the case of BPSS1385 p18, BPSS1531 p12, BPSL2096 p6, and BPSL2522 p14 (Figure 3B). Again, the markedly reduced response to strongly immunoprevalent epitopes during acute melioidosis argues a case for altered modulation of bacterial antigen expression or host antigen presentation. We further investigated whether acute melioidosis patients may generate responses to the selected immunoprevalent peptides after recovery from infection, looking at paired samples from individual donors, comparing acute and convalescent samples. We found that PBMC from septicemic patients lacked a response to the selected immunoprevalent peptides (at day 3), but could respond to them after completion of antibiotic treatment and recovery from infection (at day 30) (Figure 4). Responses to the immunoprevalent T-cell epitopes were 2–4 times greater at recovery.

## Discussion

There are estimated to be around 90,000 deaths annually from melioidosis across the globe, making development of an effective vaccine a high priority (5). A detailed understanding of protective immunity to Bp necessarily underpins such efforts, not least since the most highly susceptible individuals may often be immune-compromised, as in the large SE Asian cohort in whom diabetes is a comorbidity (9, 30, 31). Furthermore, the consideration of vaccine strategies for protection against melioidosis in a global health setting is highly overlapping with consideration of biodefense preparedness against use of either Bp or Bm as a bioweapon (9). While immunodominant antigens and correlates of protection are likely to show a high degree of overlap across these different settings, there are several key differences to consider in terms of detailed applications: while environmental Bp is likely to be encountered through contact with soil or water and to be of specific concern to susceptible individuals such as those with diabetes, weaponised Bp, or Bm is most likely to be used as an aerosolized pathogen. If efforts to leverage vaccine approaches by identifying synergies between these different threats are to flourish, there will need to be greater attention paid to differences in immune mechanisms associated with these differential modes of exposure: what are the specific requirements of a vaccine that could serve both with respect to exposure of diabetic individuals through skin lesions, as well as of immune-competent individuals across the lung mucosa?

In recent years, there have been substantial efforts to identify key Bp antigens as vaccine candidates, most commonly through analyzing protective vaccination of mice from acute Bp infection (32–40). This screening strategy has offered a relatively large number of potential immunogens. However, our focus in the present study was to adopt an approach centered on the spectrum of disease outcomes in naturally exposed human cohorts. The murine models depend generally on a single, intraperitoneal injection of a lethal dose of Bp, with or without protective vaccination during the preceding weeks, then measuring survival during short-term, acute disease. The pattern of human exposure and disease is somewhat different to this. The leads offered by the short-term murine models of acute infection undoubtedly offer important indicators as to those immunogens capable of eliciting an adaptive immune response that can significantly impact clearance of the input inoculum before there is a chance of sepsis. However, this perhaps sets the bar for protection at a different place to the challenge of conferring protection to immune-susceptible humans living in an environment supplying ongoing microbial exposure, and with a lack of any clear indication in human disease as to the immune correlates that distinguish asymptomatic and symptomatic outcomes.

In temperate countries, depending on soil and climatic conditions, there may to a variable extent be a lifetime of exposure to Bp and closely related species such as Bt, from soil, drinking water, or rainwater spray, which can lead to seroconversion in a high proportion of individuals. Adaptive and/or innate defense mechanisms appear to render healthy individuals entirely impervious to the consequences of continued, ongoing Bp exposure most of the time, except under conditions of enhanced immune susceptibility, often, diabetes. We were therefore fortunate to have the opportunity to conduct a large-scale T-cell epitope mapping screen of responses in exposed individuals from the Khon Kaen region of Thailand, comparing patterns of response between healthy seropositives, acute disease, and recovery. Mapping of T-cell activating peptides in a naturally exposed human cohort both provides crucial clues for the design of peptide-based vaccines, and also for monitoring specific immune responsiveness in the context of any trialed vaccines (28). While T-cell epitope mapping studies of large bacterial proteomes have often had to depend on screening very highly multiplexed cocktails of pooled peptides (41–45), we were keen here to obtain a pure picture of T-cell responder frequencies to individual sequences from the genome by screening against individual, overlapping peptides (28, 46). To this end, we conducted an in-depth analysis of human T-cell response to Bp peptide libraries. This showed the Bp proteome to be remarkably T-cell epitope rich. Two-hundred and thirty-five immunoprevalent peptides were identified from the top 20 candidate Bp antigens, i.e., capable of inducing a T-cell IFNγ response in ≥80% of tested seropositive human donors living in endemic area. Heatmap analysis of responses within the cohort enabled us to build a panel of 10 very commonly recognized immunoprevalent peptides, which cumulatively can account for around 1,000 SFC/10^6^ cells––i.e., 0.1% of the peripheral T-cell repertoire. Patients who had recovered from septicemic melioidosis showed a high level of T-cell IFNγ response to these epitopes compared with patients with acute septicemic melioidosis. It was noteworthy that while the T-cell response to fixed Bp overall was intact during acute sepsis, specific T-cell responses to most of these key, immunoprevalent peptides had been shut down. The notion that as much as 0.1% of the peripheral T-cell repertoire is apparently devoted to recognition and control of this one bacterial pathogen is striking. We interpret the data to mean either that lifelong reexposure to a seemingly strongly immunogenic pathogen indeed leads to exceptionally high responder cell frequencies in individuals from endemic areas, and/or that these high frequencies may also be boosted by cross-reactive antigens from phylogenetically related bacterial species. For example, a number of the epitopes described here are largely conserved in Bt. We here undertook a detailed analysis of conservation of all of the peptides analyzed, across all 20 antigen libraries, looking at conservation between pseudomallei, thailandensis and mallei (Table S3 in Supplementary Material). It should be noted in this context that, though highly related, the size of the bacterial genomes across the two chromosomes runs pseudomallei > thailandensis > mallei, in increments of an 8–9% contraction in each case. Thus, some of the key regions of Bp T-cell immunogenicity are absent from Bt and Bm. The situation can best be illustrated by considering the top 10 ranked epitope-containing peptides (Table 4): of these 7/10 are present in all three bacteria and almost entirely conserved in sequence (BPSL2522 p13, BPSL2522 p14, BPSL2522 p21, BPSS0503 p18, BPSS1532 p44, BPSS1599 p12, BPSS1599 p18). Two are present in Bp but entirely absent from the Bt and Bm genomes (BPSS1385 p14 and p18). One is present in Bp and Bt, but absent from Bm (BPSS0530 p43). The comparative analysis of immunogens between these genomes is highly informative, whether considering the potential for cross-reactive immunogenicity, such as in dual-purpose vaccines for communal/biodefense use against Bp and Bm, or considering potential for species specific diagnostics to distinguish between Bp and Bt exposure.

**Table 4:** Sequence homology between *Burkholderia pseudomallei* (Bp), *Burkholderia thailandensi*s (Bt), and *Burkholderia mallei* (Bm) for the twenty most commonly recognized Bp epitopes.

Antigen	Peptide no.	Peptide	*Burkholderia pseudomallei*	*Burkholderia thailandensis%*		*Burkholderia mallei%*	
				homology	sequence	homology	sequence
BPSS0530	P43	BPSS0530 [421-440]	KQTLAGSAMALRIVGDFPDL	100	KQTLAGSAMALRIVGDFPDL	0	–
BPSL2522	P13	BPSL2522 [121-140]	AAKIQGMNVEVVVATGYTDR	100	AAKIQGMNVEVVVATGYTDR	100	AAKIQGMNVEVVVATGYTDR
BPSL2522	P14	BPSL2522 [131-150]	VVVATGYTDRIGSDKYNDRL	100	VVVATGYTDRIGSDKYNDRL	100	VVVATGYTDRIGSDKYNDRL
BPSS1385	P14	BPSS1385 [131-150]	TDFGGLENYKELTGGADPFA	0	–	0	–
BPSL2522	P21	BPSL2522 [201-220]	APDRRVEVEVVGTQEVQKTT	100	APDRRVEVEVVGTQEVQKTT	100	APDRRVEVEVVGTQEVQKTT
BPSS0530	P18	BPSS0530 [171-190]	ALPIARVAGRNASRTVSLDP	100	ALPIARVAGRNASRTVSLDP	100	ALPIARVAGRNASRTVSLDP
BPSS1385	P18	BPSS1385 [171-190]	DVPIDPTSIEYLENTSFAEH	0	–	0	–
BPSS1599	P21	BPSS1599 [201-220]	TAACVVLGGAFAYWHHRAKV	75	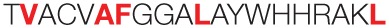	100	TAACVVLGGAFAYWHHRAKV
BPSS1532	P44	BPSS1532 [431-450]	AGAILGAVVTGVALVAAAFV	100	AGAILGAVVTGVALVAAAFV	100	AGAILGAVVTGVALVAAAFV
BPSS1599	P12	BPSS1599 [111-130]	AVRDHAFMPNGDWVGSREEA	100	AVRDHAFMPNGDWVGSREEA	100	AVRDHAFMPNGDWVGSREEA
BPSS1599	P18	BPSS1599 [171-190]	RRGGRPRTERWWALRPVERR	95	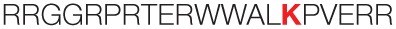	100	RRGGRPRTERWWALRPVERR
BPSS1599	P29	BPSS1599 [281-300]	ECTPGTAHYAWARNGSNVRY	100	ECTPGTAHYAWARNGSNVRY	100	ECTPGTAHYAWARNGSNVRY
BPSS1599	P20	BPSS1599 [191-210]	LSPRAALIAATAACVVLGGA	85	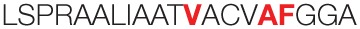	100	LSPRAALIAATAACVVLGGA
BPSS1599	P34	BPSS1599 [331-350]	TPLADDSVVRTQLLARLQWL	90	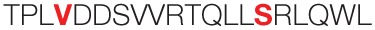	100	TPLADDSVVRTQLLARLQWL
BPSL0999	P19	BPSL0999 [181-200]	QGMGASNPIADNATEAGRAQ	100	QGMGASNPIADNATEAGRAQ	100	QGMGASNPIADNATEAGRAQ
BPSL0999	P15	BPSL0999 [141-160]	VGYTDSTGSAAHNQTLSQNR	100	VGYTDSTGSAAHNQTLSQNR	100	VGYTDSTGSAAHNQTLSQNR
BPSS1385	P21	BPSS1385 [201-220]	VVIVNDGRLGHKFLIDLPAL	0	–	0	–
BPSS1385	P11	BPSS1385 [101-120]	SNRVMWNDRYDTLLIARDPR	0	–	0	–
BPSS1385	P20	BPSS1385 [191-210]	VNTLDSHKNYVVIVNDGRLG	0	–	0	–
BPSS1385	P10	BPSS1385 [91-110]	SLMQSLSGESSNRVMWNDRY	0	–	0	

*Sequences of Bp peptides were compared with those for Bt (taxonomic identifier 57975) and Bm (taxonomic identifier 13373). Variations from the sequence for Bp are shown in red*.

The starting point for the present study was 20 serodominant antigens previously defined by ourselves with others on the basis of reactivity of patient antisera against a recombinant protein Bp antigen array (47). Here, we demonstrate that all 20 antigens which had been defined by their antibody reactivity also contain multiple T-cell epitopes. In most cases, these antigens have not been previously reported as targets of T-cell recognition. A noteworthy feature contributing to the strength and breadth of the response to immunoprevalent peptides we describe here was that T-cell recognition was in most cases able to traverse highly diverse HLA class-II haplotypes (Table S1 in Supplementary Material), as reflected also in the relatively broad HLA-DR binding profiles (Table 3). While our binding analysis was in this case limited to HLA-DR heterodimers, we acknowledge that the same epitopes may often be presented by HLA-DP and DQ heterodimers. For example, we have previously defined BPSL2096 p6 as an epitope that can be presented also through HLA-DQB1*0602 and HLA-DQB1*0302 (19). While our study was designed with a view to delineation of HLAII/CD4 epitopes, we cannot exclude the possibility that the peptides we used may have also contained within them, shorter, CD8 epitopes. While the extent to which our 20-mer peptides could have accessed the HLAI biosynthetic pathway for reprocessing, loading, and presentation is presumably limited, we have previously described examples of Bp peptides capable of presentation to both CD4 and CD8 cells (19). As an intracellular pathogen that is likely to access the endogenous HLAI pathway, Bp presumably induces protective CD8 immunity as well as CD4, which will be an important focus for future studies (17, 19, 21).

Having established that Bp carries a very large number of highly immunogenic and epitope-rich CD4 antigens, and that most of these can bind to diverse HLA II alleles, the challenge now will be to use this knowledge to gain an improved understanding of bacterial pathogenesis. It is given that Bp comes under host control through both innate (48–50) and adaptive immunity (13, 16–18, 51), yet data on immune correlates of overt melioidosis are still limited (21). Previous work from our group on acute melioidosis donors focused on the observation that patients who survived acute infection showed a T-cell IFNγ response to AhpC significantly greater than those who died (19). According to the studies shown here, the CD4 IFNγ response to immunoprevalent Bp epitopes was suppressed, but not the overall responses to whole bacteria. This is in line with accounts of immune suppression during bacterial sepsis, surviving patients showing a restoration of adaptive immunity with convalescence (52). Further analysis will be required to determine the extent to which specific immune shutdown during sepsis might be variously attributed to apoptosis, Treg control, and/or specific immune exhaustion. In addition, septicemic melioidosis patients display overexpression of many transcripts involved in inflammation and innate immune response processes (53–55) while transcripts associated with adaptive immune processes, including cytotoxicity, are underexpressed (53).

Any effective vaccine for melioidosis will need to provide broad population coverage and induce high-frequency, protective immunity against Bp. As with any vaccine strategy, Bp vaccination will be a trade-off between the immunogenicity of a live-attenuated vaccine (including Bt-based approaches) with the associated potential for adverse events, and the lesser immunogenicity but greater safety of subunit (including epitope-based) approaches (56). Our findings suggest that multivalent Bp antigen constructs or multi-epitope string vaccines may offer readily generated, potent and safe means of achieving this goal. As with other subunit vaccine approaches, appropriate adjuvants for immunogenicity with minimal boosting will be paramount. The library of highly immunogenic, protection-correlated, CD4 activating peptides described here encompass the ability to bind diverse HLA-DR alleles, suggesting that such epitope vaccines could be applicable across diverse populations.

## Ethics Statement

This study was carried out in accordance with the recommendations of Khon Kaen University Ethics Committee for Human Research (Project no. HE470506) with written informed consent from all subjects. All subjects gave written informed consent in accordance with the Declaration of Helsinki. The protocol was approved by the Khon Kaen University Ethics Committee for Human Research.

## Author Contributions

JR, RB, DA, and GL conceived the research. RB, DA, and GL oversaw the study and data analysis. PC recruited melioidosis patients Srinagarind Hospital, and provided clinical data analysis. AN, DR, SB, PS, CK, BK, CR, and PK performed the experiments on T-cell responses and data analysis. BM performed and interpreted HLA binding assay. AN, DR, RB, DA, and GL interpreted the data and wrote the manuscript. All authors read, commented on, and agreed on the content of the manuscript.

## Conflict of Interest Statement

The authors declare that the research was conducted in the absence of any commercial or financial relationships that could be construed as a potential conflict of interest.
