# Supplementary material for: Immune Control of Burkholderia pseudomallei––Common, High-Frequency T-Cell Responses to a Broad Repertoire of Immunoprevalent Epitopes
Source: Front Immunol. 2018 Mar 20;9:484. doi: 10.3389/fimmu.2018.00484 (PMC5869189; doi:10.3389/fimmu.2018.00484)
Supplement: Supplementary file 1 [file table_1.PDF]

Table S1: Demographic and HLA class II allele frequency of individuals recruited for T cell epitope mapping studies (total n = 129)

**TEM including sample**			**129 (100%)**
Male			70 (54.3%)
Female			59 (45.7%)
Age			35.7 (18-60)
**HLA frequency distribution**			
HLA DRB1 allele		***HLA DQB1 allele***	
01	***2 (0.76%)***	***02:01***	***36 (13.95%)***
01	*1*	***03***	***75 (29.07%)***
01:01	*1*	*03:(03,15)*	*1*
03	***15 (6.20%)***	*03:01*	*43*
03	*4*	*03:02*	*7*
03:01	*12*	*03:03*	*24*
04	***17 (6.60%)***	***04***	***10 (3.88%)***
04	*11*	*04:01*	*9*
04:01	*2*	*04:02*	*1*
04:05	*3*	***05***	***109 (42.25%)***
04:06	*1*	*05:01*	*40*
07	***23 (8.92%)***	*05:02*	*60*
07	*21*	*05:03*	*9*
07:01	*2*	***06***	***28 (10.85%)***
08	***10 (3.88%)***	*06:(04,17)*	*1*
08	*5*	*06:01*	*18*
08:20/13:07	*5*	*06:02*	*4*
09	***22 (8.53%)***	*06:03*	*2*
09	*19*	*06:05*	*3*
09:01	*3*		
10:01	***3 (1.16%)***		
11	***17 (6.60%)***		
11	*3*		
11:01	*14*		
12	***45 (17.44%)***		
12	*1*		
12:02	*44*		
13:01	***3 (1.16%)***		
14	***23 (8.91%)***		
14	*3*		
14:01	*16*		
14:02	*1*		
14:04	*3*		
15:01	***26 (10.08%)***		
15:02	***39 (15.12%)***		
16	***12 (4.65%)***		
16	*3*		
16:01	*7*		
16:02	*2*		
