# Supplementary material for: Immune Control of Burkholderia pseudomallei––Common, High-Frequency T-Cell Responses to a Broad Repertoire of Immunoprevalent Epitopes
Source: Front Immunol. 2018 Mar 20;9:484. doi: 10.3389/fimmu.2018.00484 (PMC5869189; doi:10.3389/fimmu.2018.00484)
Supplement: Supplementary file 2 [file table_2.PDF]

Table S2: Top 235 peptides from 20 Bp antigen peptide libraries identified in seropositive healthy donors ranked by % responders (Median IFN-γ SFC number/10^6 PBMC is shown).

Locus tag	% Responders	Median SFC/10^6 PBMCs	Protein category	Subcellular localization
BPSS0530_P43	100.0	299	Hypothetical protein BPSS0530	Cytoplasmic
BPSL2522_P13	100.0	291	Outer membrane protein A	Outer Membrane
BPSL2522_P14	100.0	290	Outer membrane protein A	Outer Membrane
BPSS1385_P14	100.0	288	ATP/GTP binding protein	Cytoplasmic
BPSL2522_P21	100.0	273	Outer membrane protein A	Outer Membrane
BPSS0530_P18	100.0	249	Hypothetical protein BPSS0530	Cytoplasmic
BPSS1385_P18	100.0	225	ATP/GTP binding protein	Cytoplasmic
BPSS1599_P21	100.0	170	Type IV pilus biosynthesis protein	Unknown
BPSS1532_P44	100.0	156	Cell invasion protein (BipB)	Extracellular
BPSS1599_P12	97.9	178	Type IV pilus biosynthesis protein	Unknown
BPSS1599_P18	97.9	172	Type IV pilus biosynthesis protein	Unknown
BPSS1599_P29	97.9	162	Type IV pilus biosynthesis protein	Unknown
BPSS1599_P20	97.9	150	Type IV pilus biosynthesis protein	Unknown
BPSS1599_P34	97.9	147	Type IV pilus biosynthesis protein	Unknown
BPSL0999_P19	97.9	375	OmpA family transmembrane protein	Outer Membrane
BPSL0999_P15	97.9	367	OmpA family transmembrane protein	Outer Membrane
BPSS1385_P21	97.8	272	ATP/GTP binding protein	Cytoplasmic
BPSS1385_P11	97.8	230	ATP/GTP binding protein	Cytoplasmic
BPSS1385_P20	97.8	212	ATP/GTP binding protein	Cytoplasmic
BPSS1385_P10	97.8	211	ATP/GTP binding protein	Cytoplasmic
BPSS0530_P19	97.7	334	Hypothetical protein BPSS0530	Cytoplasmic
BPSS0530_P12	97.7	308	Hypothetical protein BPSS0530	Cytoplasmic
BPSS0530_P17	97.7	274	Hypothetical protein BPSS0530	Cytoplasmic
BPSS0530_P13	97.7	267	Hypothetical protein BPSS0530	Cytoplasmic
BPSS0530_P11	97.7	256	Hypothetical protein BPSS0530	Cytoplasmic
BPSS0530_P31	97.7	240	Hypothetical protein BPSS0530	Cytoplasmic
BPSL0919_P20	97.3	442	4-hydroxy-3-methylbut-2-enyl diphosphate reductase	Cytoplasmic
BPSL0919_P23	97.3	405	4-hydroxy-3-methylbut-2-enyl diphosphate reductase	Cytoplasmic
BPSL2522_P23	97.1	361	Outer membrane protein A	Outer Membrane
BPSL2522_P12	97.1	308	Outer membrane protein A	Outer Membrane
BPSL2522_P15	97.1	290	Outer membrane protein A	Outer Membrane
BPSL2522_P22	97.1	270	Outer membrane protein A	Outer Membrane
BPSS1531_P21	97.1	225	Effector protein BipC	Periplasmic
BPSL2096_P12	97.1	317	Hydroperoxide reductase	Extracellular
BPSS1599_P10	95.8	189	Type IV pilus biosynthesis protein	Unknown
BPSS1599_P13	95.8	182	Type IV pilus biosynthesis protein	Unknown
BPSS1599_P19	95.8	169	Type IV pilus biosynthesis protein	Unknown
BPSS1599_P22	95.8	169	Type IV pilus biosynthesis protein	Unknown
BPSS1599_P14	95.8	157	Type IV pilus biosynthesis protein	Unknown
BPSS1599_P11	95.8	148	Type IV pilus biosynthesis protein	Unknown
BPSS1599_P7	95.8	132	Type IV pilus biosynthesis protein	Unknown
BPSL0999_P11	95.7	399	OmpA family transmembrane protein	Outer Membrane
BPSL0999_P14	95.7	373	OmpA family transmembrane protein	Outer Membrane
BPSS1385_P15	95.6	228	ATP/GTP binding protein	Cytoplasmic
BPSS0530_P32	95.3	316	Hypothetical protein BPSS0530	Cytoplasmic
BPSS0530_P36	95.3	298	Hypothetical protein BPSS0530	Cytoplasmic
BPSS0530_P14	95.3	279	Hypothetical protein BPSS0530	Cytoplasmic
BPSL0919_P12	94.6	441	4-hydroxy-3-methylbut-2-enyl diphosphate reductase	Cytoplasmic
BPSL0919_P21	94.6	422	4-hydroxy-3-methylbut-2-enyl diphosphate reductase	Cytoplasmic
BPSL2522_P18	94.3	288	Outer membrane protein A	Outer Membrane
BPSS1531_P13	94.3	239	Effector protein BipC	Periplasmic
BPSS1531_P15	94.3	235	Effector protein BipC	Periplasmic
BPSS1531_P18	94.3	230	Effector protein BipC	Periplasmic
BPSS1531_P23	94.3	221	Effector protein BipC	Periplasmic
BPSS1531_P29	94.3	216	Effector protein BipC	Periplasmic
BPSS1531_P22	94.3	215	Effector protein BipC	Periplasmic
BPSS1531_P37	94.3	178	Effector protein BipC	Periplasmic
BPSS1531_P35	94.3	148	Effector protein BipC	Periplasmic
BPSL2096_P13	94.1	315	Hydroperoxide reductase	Extracellular
BPSL1445_P12	93.9	321	Putative lipoprotein	Extracellular
BPSS1599_P37	93.8	147	Type IV pilus biosynthesis protein	Unknown
BPSS1599_P35	93.8	145	Type IV pilus biosynthesis protein	Unknown
BPSL0999_P10	93.6	382	OmpA family transmembrane protein	Outer Membrane

Locus tag	% Responders	Median SFU/10^6 PBMCs	Protein category	Subcellular localization
BPSL0999_P18	93.6	375	OmpA family transmembrane protein	Outer Membrane
BPSL0999_P20	93.6	353	OmpA family transmembrane protein	Outer Membrane
BPSL0999_P21	93.6	348	OmpA family transmembrane protein	Outer Membrane
BPSS1385_P12	93.3	291	ATP/GTP binding protein	Cytoplasmic
BPSS1385_P23	93.3	275	ATP/GTP binding protein	Cytoplasmic
BPSS1385_P28	93.3	195	ATP/GTP binding protein	Cytoplasmic
BPSS1385_P3	93.3	195	ATP/GTP binding protein	Cytoplasmic
BPSL0280_P18	93.1	523	Flagellar hook-associated protein (FlgK)	Extracellular (flagella)
BPSL0280_P21	93.1	493	Flagellar hook-associated protein (FlgK)	Extracellular (flagella)
BPSL0280_P22	93.1	464	Flagellar hook-associated protein (FlgK)	Extracellular (flagella)
BPSL0280_P29	93.1	463	Flagellar hook-associated protein (FlgK)	Extracellular (flagella)
BPSS0530_P10	93.0	280	Hypothetical protein BPSS0530	Cytoplasmic
BPSS0530_P1	93.0	258	Hypothetical protein BPSS0530	Cytoplasmic
BPSS0530_P21	93.0	258	Hypothetical protein BPSS0530	Cytoplasmic
BPSS0530_P15	93.0	255	Hypothetical protein BPSS0530	Cytoplasmic
BPSS0530_P29	93.0	234	Hypothetical protein BPSS0530	Cytoplasmic
BPSS0530_P5	93.0	224	Hypothetical protein BPSS0530	Cytoplasmic
BPSS1525_P20	92.3	216	Guanine nucleotide exchange factor BopE	Extracellular
BPSS1525_P13	92.3	200	Guanine nucleotide exchange factor BopE	Extracellular
BPSL0919_P11	91.9	449	4-hydroxy-3-methylbut-2-enyl diphosphate reductase	Cytoplasmic
BPSL0919_P15	91.9	433	4-hydroxy-3-methylbut-2-enyl diphosphate reductase	Cytoplasmic
BPSL0919_P19	91.9	427	4-hydroxy-3-methylbut-2-enyl diphosphate reductase	Cytoplasmic
BPSL0919_P29	91.9	394	4-hydroxy-3-methylbut-2-enyl diphosphate reductase	Cytoplasmic
BPSS1599_P30	91.7	155	Type IV pilus biosynthesis protein	Unknown
BPSS1599_P23	91.7	147	Type IV pilus biosynthesis protein	Unknown
BPSS1599_P42	91.7	139	Type IV pilus biosynthesis protein	Unknown
BPSL0999_P13	91.5	357	OmpA family transmembrane protein	Outer Membrane
BPSL0999_P6	91.5	357	OmpA family transmembrane protein	Outer Membrane
BPSL2522_P4	91.4	368	Outer membrane protein A	Outer Membrane
BPSL2504_P8	91.4	317	Hydrolase	Cytoplasmic
BPSL2522_P10	91.4	297	Outer membrane protein A	Outer Membrane
BPSL2522_P6	91.4	291	Outer membrane protein A	Outer Membrane
BPSS1531_P11	91.4	235	Effector protein BipC	Periplasmic
BPSS1531_P14	91.4	229	Effector protein BipC	Periplasmic
BPSS1531_P20	91.4	225	Effector protein BipC	Periplasmic
BPSS1531_P30	91.4	213	Effector protein BipC	Periplasmic
BPSS1531_P36	91.4	205	Effector protein BipC	Periplasmic
BPSL2096_P14	91.2	344	Hydroperoxide reductase	Extracellular
BPSL2096_P3	91.2	308	Hydroperoxide reductase	Extracellular
BPSS1385_P9	91.1	256	ATP/GTP binding protein	Cytoplasmic
BPSS1385_P13	91.1	229	ATP/GTP binding protein	Cytoplasmic
BPSS1385_P7	91.1	224	ATP/GTP binding protein	Cytoplasmic
BPSS1385_P4	91.1	206	ATP/GTP binding protein	Cytoplasmic
BPSS1385_P5	91.1	187	ATP/GTP binding protein	Cytoplasmic
BPSS0530_P38	90.7	305	Hypothetical protein BPSS0530	Cytoplasmic
BPSS0530_P6	90.7	288	Hypothetical protein BPSS0530	Cytoplasmic
BPSS0530_P9	90.7	283	Hypothetical protein BPSS0530	Cytoplasmic
BPSS0530_P23	90.7	267	Hypothetical protein BPSS0530	Cytoplasmic
BPSS0530_P2	90.7	260	Hypothetical protein BPSS0530	Cytoplasmic
BPSS0530_P20	90.7	248	Hypothetical protein BPSS0530	Cytoplasmic
BPSS0530_P35	90.7	243	Hypothetical protein BPSS0530	Cytoplasmic
BPSS0530_P44	90.7	225	Hypothetical protein BPSS0530	Cytoplasmic
BPSS0530_P7	90.7	211	Hypothetical protein BPSS0530	Cytoplasmic
BPSS1525_P15	89.7	220	Guanine nucleotide exchange factor BopE	Extracellular
BPSS1525_P5	89.7	178	Guanine nucleotide exchange factor BopE	Extracellular
BPSL0280_P14	89.7	565	Flagellar hook-associated protein (FlgK)	Extracellular (flagella)
BPSS1599_P26	89.6	164	Type IV pilus biosynthesis protein	Unknown
BPSS1599_P3	89.6	156	Type IV pilus biosynthesis protein	Unknown
BPSS1599_P36	89.6	143	Type IV pilus biosynthesis protein	Unknown
BPSS1599_P40	89.6	137	Type IV pilus biosynthesis protein	Unknown
BPSS1599_P5	89.6	129	Type IV pilus biosynthesis protein	Unknown
BPSS1599_P38	89.6	125	Type IV pilus biosynthesis protein	Unknown
BPSS1599_P8	89.6	99	Type IV pilus biosynthesis protein	Unknown

Locus tag	% Responders	Median SFU/10^6 PBMCs	Protein category	Subcellular localization
BPSL0919_P13	89.2	474	4-hydroxy-3-methylbut-2-enyl diphosphate reductase	Cytoplasmic
BPSL0919_P2	89.2	444	4-hydroxy-3-methylbut-2-enyl diphosphate reductase	Cytoplasmic
BPSL0919_P28	89.2	434	4-hydroxy-3-methylbut-2-enyl diphosphate reductase	Cytoplasmic
BPSL0919_P31	89.2	405	4-hydroxy-3-methylbut-2-enyl diphosphate reductase	Cytoplasmic
BPSL0919_P30	89.2	389	4-hydroxy-3-methylbut-2-enyl diphosphate reductase	Cytoplasmic
BPSS1385_P2	88.9	274	ATP/GTP binding protein	Cytoplasmic
BPSS1385_P24	88.9	267	ATP/GTP binding protein	Cytoplasmic
BPSS1385_P22	88.9	200	ATP/GTP binding protein	Cytoplasmic
BPSL2522_P9	88.6	353	Outer membrane protein A	Outer Membrane
BPSL2522_P2	88.6	273	Outer membrane protein A	Outer Membrane
BPSL2522_P3	88.6	267	Outer membrane protein A	Outer Membrane
BPSS1531_P10	88.6	243	Effector protein BipC	Periplasmic
BPSS1531_P19	88.6	226	Effector protein BipC	Periplasmic
BPSS1531_P27	88.6	212	Effector protein BipC	Periplasmic
BPSS1531_P38	88.6	198	Effector protein BipC	Periplasmic
BPSS1531_P12	88.6	182	Effector protein BipC	Periplasmic
BPSS1531_P31	88.6	182	Effector protein BipC	Periplasmic
BPSS1531_P34	88.6	180	Effector protein BipC	Periplasmic
BPSS1531_P39	88.6	178	Effector protein BipC	Periplasmic
BPSS1531_P5	88.6	159	Effector protein BipC	Periplasmic
BPSS0530_P30	88.4	310	Hypothetical protein BPSS0530	Cytoplasmic
BPSS0530_P28	88.4	260	Hypothetical protein BPSS0530	Cytoplasmic
BPSS0530_P22	88.4	247	Hypothetical protein BPSS0530	Cytoplasmic
BPSS0530_P26	88.4	240	Hypothetical protein BPSS0530	Cytoplasmic
BPSL2096_P10	88.2	351	Hydroperoxide reductase	Extracellular
BPSL2096_P2	88.2	323	Hydroperoxide reductase	Extracellular
BPSL2096_P11	88.2	320	Hydroperoxide reductase	Extracellular
BPSL2096_P4	88.2	305	Hydroperoxide reductase	Extracellular
BPSS1599_P15	87.5	179	Type IV pilus biosynthesis protein	Unknown
BPSS1599_P28	87.5	163	Type IV pilus biosynthesis protein	Unknown
BPSS1599_P32	87.5	148	Type IV pilus biosynthesis protein	Unknown
BPSS1599_P39	87.5	143	Type IV pilus biosynthesis protein	Unknown
BPSS1599_P41	87.5	135	Type IV pilus biosynthesis protein	Unknown
BPSS1599_P24	87.5	119	Type IV pilus biosynthesis protein	Unknown
BPSS1525_P21	87.2	179	Guanine nucleotide exchange factor BopE	Extracellular
BPSS1532_P35	87.1	172	Cell invasion protein (BipB)	Extracellular
BPSS1385_P19	86.7	276	ATP/GTP binding protein	Cytoplasmic
BPSL3319_P27	86.7	259	Flagellin	Extracellular (flagella)
BPSS1385_P16	86.7	216	ATP/GTP binding protein	Cytoplasmic
BPSS1385_P6	86.7	203	ATP/GTP binding protein	Cytoplasmic
BPSL0919_P22	86.5	461	4-hydroxy-3-methylbut-2-enyl diphosphate reductase	Cytoplasmic
BPSL0919_P10	86.5	452	4-hydroxy-3-methylbut-2-enyl diphosphate reductase	Cytoplasmic
BPSL0919_P4	86.5	369	4-hydroxy-3-methylbut-2-enyl diphosphate reductase	Cytoplasmic
BPSL0919_P7	86.5	368	4-hydroxy-3-methylbut-2-enyl diphosphate reductase	Cytoplasmic
BPSS0530_P34	86.0	299	Hypothetical protein BPSS0530	Cytoplasmic
BPSS0530_P27	86.0	239	Hypothetical protein BPSS0530	Cytoplasmic
BPSS0530_P33	86.0	233	Hypothetical protein BPSS0530	Cytoplasmic
BPSS0530_P39	86.0	221	Hypothetical protein BPSS0530	Cytoplasmic
BPSL2522_P11	85.7	408	Outer membrane protein A	Outer Membrane
BPSS1531_P26	85.7	219	Effector protein BipC	Periplasmic
BPSS1531_P40	85.7	198	Effector protein BipC	Periplasmic
BPSS1531_P6	85.7	194	Effector protein BipC	Periplasmic
BPSS1531_P33	85.7	164	Effector protein BipC	Periplasmic
BPSS1599_P4	85.4	137	Type IV pilus biosynthesis protein	Unknown
BPSS1599_P6	85.4	129	Type IV pilus biosynthesis protein	Unknown
BPSS1599_P9	85.4	126	Type IV pilus biosynthesis protein	Unknown
BPSL2096_P7	85.3	304	Hydroperoxide reductase	Extracellular
BPSL0999_P16	85.1	350	OmpA family transmembrane protein	Outer Membrane
BPSS1525_P12	84.6	201	Guanine nucleotide exchange factor BopE	Extracellular
BPSS1525_P11	84.6	184	Guanine nucleotide exchange factor BopE	Extracellular
BPSS1385_P27	84.4	263	ATP/GTP binding protein	Cytoplasmic
BPSS1385_P29	84.4	262	ATP/GTP binding protein	Cytoplasmic
BPSS1385_P31	84.4	192	ATP/GTP binding protein	Cytoplasmic

Locus tag	% Responders	Median SFU/10^6 PBMCs	Protein category	Subcellular localization
BPSS1532_P45	83.9	142	Cell invasion protein (BipB)	Extracellular
BPSL0919_P18	83.8	447	4-hydroxy-3-methylbut-2-enyl diphosphate reductase	Cytoplasmic
BPSL0919_P32	83.8	419	4-hydroxy-3-methylbut-2-enyl diphosphate reductase	Cytoplasmic
BPSL0919_P24	83.8	398	4-hydroxy-3-methylbut-2-enyl diphosphate reductase	Cytoplasmic
BPSL0919_P33	83.8	386	4-hydroxy-3-methylbut-2-enyl diphosphate reductase	Cytoplasmic
BPSS0530_P3	83.7	253	Hypothetical protein BPSS0530	Cytoplasmic
BPSS0530_P4	83.7	239	Hypothetical protein BPSS0530	Cytoplasmic
BPSS0530_P42	83.7	237	Hypothetical protein BPSS0530	Cytoplasmic
BPSS0530_P37	83.7	222	Hypothetical protein BPSS0530	Cytoplasmic
BPSS1599_P31	83.3	151	Type IV pilus biosynthesis protein	Unknown
BPSS1599_P33	83.3	147	Type IV pilus biosynthesis protein	Unknown
BPSS1599_P16	83.3	142	Type IV pilus biosynthesis protein	Unknown
BPSS1599_P17	83.3	141	Type IV pilus biosynthesis protein	Unknown
BPSL0999_P12	83.0	358	OmpA family transmembrane protein	Outer Membrane
BPSL2522_P16	82.9	317	Outer membrane protein A	Outer Membrane
BPSL2522_P17	82.9	312	Outer membrane protein A	Outer Membrane
BPSL2522_P7	82.9	265	Outer membrane protein A	Outer Membrane
BPSS1531_P2	82.9	219	Effector protein BipC	Periplasmic
BPSS1531_P28	82.9	163	Effector protein BipC	Periplasmic
BPSS1531_P3	82.9	162	Effector protein BipC	Periplasmic
BPSL0280_P12	82.8	557	Flagellar hook-associated protein (FlgK)	Extracellular (flagella)
BPSL2096_P6	82.4	333	Hydroperoxide reductase	Extracellular
BPSL2096_P15	82.4	314	Hydroperoxide reductase	Extracellular
BPSS1385_P25	82.2	206	ATP/GTP binding protein	Cytoplasmic
BPSS1385_P30	82.2	193	ATP/GTP binding protein	Cytoplasmic
BPSS1525_P6	82.1	245	Guanine nucleotide exchange factor BopE	Extracellular
BPSS1525_P14	82.1	200	Guanine nucleotide exchange factor BopE	Extracellular
BPSS1525_P16	82.1	197	Guanine nucleotide exchange factor BopE	Extracellular
BPSS1525_P19	82.1	197	Guanine nucleotide exchange factor BopE	Extracellular
BPSS0530_P41	81.4	292	Hypothetical protein BPSS0530	Cytoplasmic
BPSS0530_P16	81.4	266	Hypothetical protein BPSS0530	Cytoplasmic
BPSS0530_P25	81.4	244	Hypothetical protein BPSS0530	Cytoplasmic
BPSS0530_P40	81.4	219	Hypothetical protein BPSS0530	Cytoplasmic
BPSS1599_P1	81.3	189	Type IV pilus biosynthesis protein	Unknown
BPSS1599_P2	81.3	137	Type IV pilus biosynthesis protein	Unknown
BPSS1599_P43	81.3	120	Type IV pilus biosynthesis protein	Unknown
BPSL0919_P26	81.1	415	4-hydroxy-3-methylbut-2-enyl diphosphate reductase	Cytoplasmic
BPSL0999_P5	80.9	346	OmpA family transmembrane protein	Outer Membrane
BPSL0999_P7	80.9	322	OmpA family transmembrane protein	Outer Membrane
BPSS1532_P54	80.6	189	Cell invasion protein (BipB)	Extracellular
BPSS1385_P26	80.0	265	ATP/GTP binding protein	Cytoplasmic
BPSS1385_P17	80.0	226	ATP/GTP binding protein	Cytoplasmic
BPSS1385_P32	80.0	208	ATP/GTP binding protein	Cytoplasmic
BPSS1531_P7	80.0	199	Effector protein BipC	Periplasmic
BPSS1531_P41	80.0	197	Effector protein BipC	Periplasmic
BPSS1531_P16	80.0	169	Effector protein BipC	Periplasmic
