# Supplementary material for: Immune Control of Burkholderia pseudomallei––Common, High-Frequency T-Cell Responses to a Broad Repertoire of Immunoprevalent Epitopes
Source: Front Immunol. 2018 Mar 20;9:484. doi: 10.3389/fimmu.2018.00484 (PMC5869189; doi:10.3389/fimmu.2018.00484)
Supplement: Supplementary file 3 [file table_3.PDF]

Table S3 Sequence homology between *Burkholderia pseudomallei, Burkholderia thailandensis* and *Burkholderia mallei* for the 20 antigen peptide panels. Sequences of Bp epitopes were blasted, using NCBI blastp, against *Burkholderia thailandensis* (taxonomic identifier 57975) and *Burkholderia mallei* (taxonomic identifier 13373). Variations from the *Burkholderia pseudomallei* sequence are shown in red.

BPSL0280

	Antigen BPSL0280	*Burkholderia pseudomallei*	*Burkholderia thailandensis*		*Burkholderia mallei*	
Peptide No.	Peptide		% homology	Sequence	% homology	Sequence
1	BPSL0280 [1-20]	MSNTLMNLGVSGLNAALWGL	100	MSNTLMNLGVSGLNAALWGL	100	MSNTLMNLGVSGLNAALWGL
2	BPSL0280 [11-30]	SGLNAALWGLTTTGQNISNA	100	SGLNAALWGLTTTGQNISNA	100	SGLNAALWGLTTTGQNISNA
3	BPSL0280 [21-40]	TTTGQNISNAATPGYSVERP	100	TTTGQNISNAATPGYSVERP	100	TTTGQNISNAATPGYSVERP
4	BPSL0280 [31-50]	ATPGYSVERPVYAEASGQYT	100	ATPGYSVERPVYAEASGQYT	100	ATPGYSVERPVYAEASGQYT
5	BPSL0280 [41-60]	VYAEASGQYTSSGYLPQGVS	100	VYAEASGQYTSSGYLPQGVS	100	VYAEASGQYTSSGYLPQGVS
6	BPSL0280 [51-70]	SSGYLPQGVSTVTVERQYNQ	100	SSGYLPQGVSTVTVERQYNQ	100	SSGYLPQGVSTVTVERQYNQ
7	BPSL0280 [61-80]	TVTVERQYNQYLSNQLNAAQ	100	TVTVERQYNQYLSNQLNAAQ	100	TVTVERQYNQYLSNQLNAAQ
8	BPSL0280 [71-90]	YLSNQLNAAQTQGSSLSTYY	100	YLSNQLNAAQTQGSSLSTYY	100	YLSNQLNAAQTQGSSLSTYY
9	BPSL0280 [81-100]	TQGSSLSTYYTLVAQLNNYV	100	TQGSSLSTYYTLVAQLNNYV	100	TQGSSLSTYYTLVAQLNNYV
10	BPSL0280 [91-110]	TLVAQLNNYVGSPTAGIATA	100	TLVAQLNNYVGSPTAGIATA	100	TLVAQLNNYVGSPTAGIATA
11	BPSL0280 [101-120]	GSPTAGIATAITNYFTGLQT	100	GSPTAGIATAITNYFTGLQT	100	GSPTAGIATAITNYFTGLQT
12	BPSL0280 [111-130]	ITNYFTGLQTVANNAADPSA	100	ITNYFTGLQTVANNAADPSA	100	ITNYFTGLQTVANNAADPSA
13	BPSL0280 [121-140]	VANNAADPSARQTAMSNAQT	100	VANNAADPSARQTAMSNAQT	100	VANNAADPSARQTAMSNAQT
14	BPSL0280 [131-150]	RQTAMSNAQTLASQLVAAGQ	100	RQTAMSNAQTLASQLVAAGQ	100	RQTAMSNAQTLASQLVAAGQ
15	BPSL0280 [141-160]	LASQLVAAGQQYSQLRQSVN	100	LASQLVAAGQQYSQLRQSVN	100	LASQLVAAGQQYSQLRQSVN
16	BPSL0280 [151-170]	QYSQLRQSVNSQLTDTVTQI	100	QYSQLRQSVNSQLTDTVTQI	100	QYSQLRQSVNSQLTDTVTQI
17	BPSL0280 [161-180]	SQLTDTVTQINSYTSQIAQL	100	SQLTDTVTQINSYTSQIAQL	100	SQLTDTVTQINSYTSQIAQL
18	BPSL0280 [171-190]	NSYTSQIAQLNEQIASASSQ	100	NSYTSQIAQLNEQIASASSQ	100	NSYTSQIAQLNEQIASASSQ
19	BPSL0280 [181-200]	NEQIASASSQGQPPNQLLDQ	100	NEQIASASSQGQPPNQLLDQ	100	NEQIASASSQGQPPNQLLDQ
20	BPSL0280 [191-210]	GQPPNQLLDQRDLAVSKLSQ	100	GQPPNQLLDQRDLAVSKLSQ	100	GQPPNQLLDQRDLAVSKLSQ
21	BPSL0280 [201-220]	RDLAVSKLSQLAGVQVVQSN	85	RDLAVSKLSQFAGVQVVPTN	100	RDLAVSKLSQLAGVQVVQSN
22	BPSL0280 [211-230]	LAGVQVVQSNGNYSVFLSGG	75	LAGVQVVPTNGSYSVFLAGG	100	LAGVQVVQSNGNYSVFLSGG
23	BPSL0280 [221-240]	GNYSVFLSGGQPLVVGNASY	90	GSYSVFLAGGQPLVVGNASY	100	GNYSVFLSGGQPLVVGNASY
24	BPSL0280 [231-250]	QPLVVGNASYQLATVASPSD	90	QPLVVGNASYQLAAVASKSD	100	QPLVVGNASYQLATVASPSD
25	BPSL0280 [241-260]	QLATVASPSDPSELTIVSKG	85	QLAAVASKSDPSELTIVSNG	100	QLATVASPSDPSELTIVSKG
26	BPSL0280 [251-270]	PSELTIVSKGVAGSAQPGPT	70	PSELTIVSNGVAG-ANPQGS	100	PSELTIVSKGVAGSAQPGPT
27	BPSL0280 [261-280]	VAGSAQPGPTQYLPDVSLTG	70	VAG-ANPQGSPQYLPDASLTG	100	VAGSAQPGPTQYLPDVSLTG
28	BPSL0280 [271-290]	QYLPDVSLTGGALGGLLAFR	90	QYLPDASLTGGTLGGLLAFR	100	QYLPDVSLTGGALGGLLAFR
29	BPSL0280 [281-300]	GALGGLLAFRSQTLDPAQAQ	95	GTLGGLLAFRSQTLDPAQAQ	100	GALGGLLAFRSQTLDPAQAQ
30	BPSL0280 [291-310]	SQTLDPAQAQLGALAVSFAS	100	SQTLDPAQAQLGALAVSFAS	100	SQTLDPAQAQLGALAVSFAS
31	BPSL0280 [301-320]	LGALAVSFASQVNAQNALGV	100	LGALAVSFASQVNAQNALGV	100	LGALAVSFASQVNAQNALGV
32	BPSL0280 [311-330]	QVNAQNALGVDMSGNPGGSL	95	QVNAQNALGVDMSGNPGGNL	100	QVNAQNALGVDMSGNPGGSL
33	BPSL0280 [321-340]	DMSGNPGGSLFAVGAPAVYA	75	DMSGNPGGNLFTAGSPIVYA	100	DMSGNPGGSLFAVGAPAVYA
34	BPSL0280 [331-350]	FAVGAPAVYANQNNTGSATL	65	FTAGSPIVYANQGNTSSSTL	100	FAVGAPAVYANQNNTGSATL
35	BPSL0280 [341-360]	NQNNTGSATLSVSFVDGTQP	60	NQGNTSSSTLSASIANGAQP	100	NQNNTGSATLSVSFVDGTQP
36	BPSL0280 [351-370]	SVSFVDGTQPTTSDYALSYD	65	SASIANGAQPPSSDYALSYD	100	SVSFVDGTQPTTSDYALSYD
37	BPSL0280 [361-380]	TTSDYALSYDGAKYTLTDRA	85	PSSDYALSYDGSKYTLTDRA	100	TTSDYALSYDGAKYTLTDRA
38	BPSL0280 [371-390]	GAKYTLTDRATGSVVGTATP	95	GSKYTLTDRATGSVVGTATP	100	GAKYTLTDRATGSVVGTATP
39	BPSL0280 [381-400]	TGSVVGTATPSSTPPTMTIG	85	TGSVVGTATPATNPPTMTIG	100	TGSVVGTATPSSTPPTMTIG
40	BPSL0280 [391-410]	SSTPPTMTIGGLKLSLSSTP	75	ATNPPTMTIGGLNLSLSATP	100	SSTPPTMTIGGLKLSLSSTP
41	BPSL0280 [401-420]	GLKLSLSSTPNAGDSFTVLP	90	GLNLSLSATPNAGDSFTVLP	100	GLKLSLSSTPNAGDSFTVLP
42	BPSL0280 [411-430]	NAGDSFTVLPTRGALDGFSL	95	NAGDSFTVLPTRGALEGFSL	100	NAGDSFTVLPTRGALDGFSL
43	BPSL0280 [421-440]	TRGALDGFSLATANGSAIAA	95	TRGALEGFSLATANGSAIAA	100	TRGALDGFSLATANGSAIAA
44	BPSL0280 [431-450]	ATANGSAIAAASPVLAAGVA	95	ATANGSAIAAASPVLAAGVS	100	ATANGSAIAAASPVLAAGVA
45	BPSL0280 [441-460]	ASPVLAAGVATNSGTGVISQ	95	ASPVLAAGVSTNSGTGVISQ	100	ASPVLAAGVATNSGTGVISQ
46	BPSL0280 [451-470]	TNSGTGVISQGSVSAGYQLP	95	TNSGTGVISQGAVSAGYQLP	100	TNSGTGVISQGSVSAGYQLP
47	BPSL0280 [461-480]	GSVSAGYQLPSGTTTLAYNA	85	GAVSAGYQLPAGTTTLTYNA	100	GSVSAGYQLPSGTTTLAYNA
48	BPSL0280 [471-490]	SGTTTLAYNAASKTLSGFPV	85	AGTTTLTYNASSKTLSGFPV	100	SGTTTLAYNAASKTLSGFPV
49	BPSL0280 [481-500]	ASKTLSGFPVGTTVTIAGTP	95	SSKTLSGFPVGTTVTIAGTP	100	ASKTLSGFPVGTTVTIAGTP
50	BPSL0280 [491-510]	GTTVTIAGTPPTSINITSAT	100	GTTVTIAGTPPTSINITSAT	100	GTTVTIAGTPPTSINITSAT
51	BPSL0280 [501-520]	PTSINITSATTPVPYDPSKG	95	PTSINITSATTPVPYDPTKG	100	PTSINITSATTPVPYDPSKG
52	BPSL0280 [511-530]	TPVPYDPSKGASMTISSTTQ	90	TPVPYDPTKGASLTISSTTQ	100	TPVPYDPSKGASMTISSTTQ
53	BPSL0280 [521-540]	ASMTISSTTQPAPSGVMNGV	95	ASLTISSTTQPAPSGVMNGV	100	ASMTISSTTQPAPSGVMNGV
54	BPSL0280 [531-550]	PAPSGVMNGVSVSLSGTPAD	100	PAPSGVMNGVSVSLSGTPAD	100	PAPSGVMNGVSVSLSGTPAD
55	BPSL0280 [541-560]	SVSLSGTPADGDQFTIGANK	100	SVSLSGTPADGDQFTIGANK	100	SVSLSGTPADGDQFTIGANK
56	BPSL0280 [551-570]	GDQFTIGANKGTNDGRNALA	100	GDQFTIGANKGTNDGRNALA	100	GDQFTIGANKGTNDGRNALA
57	BPSL0280 [561-580]	GTNDGRNALALSQLVNSKTM	100	GTNDGRNALALSQLVNSKTM	100	GTNDGRNALALSQLVNSKTM
58	BPSL0280 [571-590]	LSQLVNSKTMNNGTTTLTGA	100	LSQLVNSKTMNNGTTTLTGA	100	LSQLVNSKTMNNGTTTLTGA
59	BPSL0280 [581-600]	NNGTTTLTGAYAGYVNAIGN	100	NNGTTTLTGAYAGYVNAIGN	100	NNGTTTLTGAYAGYVNAIGN
60	BPSL0280 [591-610]	YAGYVNAIGNAASQLKASSA	100	YAGYVNAIGNAASQLKASSA	100	YAGYVNAIGNAASQLKASSA
61	BPSL0280 [601-620]	AASQLKASSAAQTALVGQIT	100	AASQLKASSAAQTALVGQIT	100	AASQLKASSAAQTALVGQIT
62	BPSL0280 [611-630]	AQTALVGQITQAQQSVSGVN	100	AQTALVGQITQAQQSVSGVN	100	AQTALVGQITQAQQSVSGVN
63	BPSL0280 [621-640]	QAQQSVSGVNQNEEAANLMQ	100	QAQQSVSGVNQNEEAANLMQ	100	QAQQSVSGVNQNEEAANLMQ
64	BPSL0280 [631-650]	QNEEAANLMQYQQLYQANAK	100	QNEEAANLMQYQQLYQANAK	100	QNEEAANLMQYQQLYQANAK
65	BPSL0280 [641-660]	YQQLYQANAKVIQTANSVFQ	100	YQQLYQANAKVIQTANSVFQ	100	YQQLYQANAKVIQTANSVFQ
66	BPSL0280 [651-667]	VIQTANSVFQTVLGLFN	100	VIQTANSVFQTVLGLFN	100	VIQTANSVFQTVLGLFN
67	BPSL0280 [648-667]	NAKVIQTANSVFQTVLGLFN	100	NAKVIQTANSVFQTVLGLFN	100	NAKVIQTANSVFQTVLGLFN

BPSL0919

	Antigen BPSL0919	*Burkholderia pseudomallei*	*Burkholderia thailandensis*		*Burkholderia mallei*	
Peptide No.	Peptide		% homology	Sequence	% homology	Sequence
1	BPSL0919 [1-20]	MSSTDTLSGQVAAADAEILL	100	MSSTDTLSGQVAAADAEILL	0	
2	BPSL0919 [11-30]	VAAADAEILLAQPRGFCAGV	100	VAAADAEILLAQPRGFCAGV	55	**MRVI**LAQPRGFCAGV
3	BPSL0919 [21-40]	AQPRGFCAGVDRAIEIVERA	100	AQPRGFCAGVDRAIEIVERA	90	AQPRGFCAGV**V**RAI**G**IVERA
4	BPSL0919 [31-50]	DRAIEIVERAIAMHGAPIYV	100	DRAIEIVERAIAMHGAPIYV	70	**V**RAI**G**IVERA**LQQN**GAPIYV
5	BPSL0919 [41-60]	IAMHGAPIYVRHEIVHNKYV	100	IAMHGAPIYVRHEIVHNKYV	80	**LQQN**GAPIYVRHEIVHNKYV
6	BPSL0919 [51-70]	RHEIVHNKYVVEDLKKKGAI	100	RHEIVHNKYVVEDLKKKGAI	100	RHEIVHNKYVVEDLKKKGAI
7	BPSL0919 [61-80]	VEDLKKKGAIFVEELEEVPS	100	VEDLKKKGAIFVEELEEVPS	100	VEDLKKKGAIFVEELEEVPS
8	BPSL0919 [71-90]	FVEELEEVPSGNTVIFSAHG	100	FVEELEEVPSGNTVIFSAHG	100	FVEELEEVPSGNTVIFSAHG
9	BPSL0919 [81-100]	GNTVIFSAHGVSKAVRDEAA	100	GNTVIFSAHGVSKAVRDEAA	100	GNTVIFSAHGVSKAVRDEAA
10	BPSL0919 [91-110]	VSKAVRDEAAVRGLRIYDAT	100	VSKAVRDEAAVRGLRIYDAT	100	VSKAVRDEAAVRGLRIYDAT
11	BPSL0919 [101-120]	VRGLRIYDATCPLVTKVHVE	100	VRGLRIYDATCPLVTKVHVE	100	VRGLRIYDATCPLVTKVHVE
12	BPSL0919 [111-130]	CPLVTKVHVEVAKMRQEGVD	100	CPLVTKVHVEVAKMRQEGVD	100	CPLVTKVHVEVAKMRQEGVD
13	BPSL0919 [121-140]	VAKMRQEGVDIVMIGHKGHP	100	VAKMRQEGVDIVMIGHKGHP	100	VAKMRQEGVDIVMIGHKGHP
14	BPSL0919 [131-150]	IVMIGHKGHPEVEGTMGQVE	100	IVMIGHKGHPEVEGTMGQVE	100	IVMIGHKGHPEVEGTMGQVE
15	BPSL0919 [141-160]	EVEGTMGQVERGMHLVESVE	100	EVEGTMGQVERGMHLVESVE	100	EVEGTMGQVERGMHLVESVE
16	BPSL0919 [151-170]	RGMHLVESVEDVRRLELPDP	90	RGMHLVESVEDV**LK**LELPDP	100	RGMHLVESVEDVRRLELPDP
17	BPSL0919 [161-180]	DVRRLELPDPERVALVTQTT	90	DV**LK**LELPDPERVALVTQTT	100	DVRRLELPDPERVALVTQTT
18	BPSL0919 [171-190]	ERVALVTQTTLSVDDAAEII	100	ERVALVTQTTLSVDDAAEII	100	ERVALVTQTTLSVDDAAEII
19	BPSL0919 [181-200]	LSVDDAAEIIGALKAKFPAI	100	LSVDDAAEIIGALKAKFPAI	100	LSVDDAAEIIGALKAKFPAI
20	BPSL0919 [191-210]	GALKAKFPAIREPKKQDICY	100	GALKAKFPAIREPKKQDICY	100	GALKAKFPAIREPKKQDICY
21	BPSL0919 [201-220]	REPKKQDICYATQNRQDAVK	100	REPKKQDICYATQNRQDAVK	100	REPKKQDICYATQNRQDAVK
22	BPSL0919 [211-230]	ATQNRQDAVKFMAPQCDVVI	100	ATQNRQDAVKFMAPQCDVVI	100	ATQNRQDAVKFMAPQCDVVI
23	BPSL0919 [221-240]	FMAPQCDVVIVVGSPNSSNS	100	FMAPQCDVVIVVGSPNSSNS	100	FMAPQCDVVIVVGSPNSSNS
24	BPSL0919 [231-250]	VVGSPNSSNSSRLREVAEKR	100	VVGSPNSSNSSRLREVAEKR	100	VVGSPNSSNSSRLREVAEKR
25	BPSL0919 [241-260]	SRLREVAEKRGVAAYMVDAP	100	SRLREVAEKRGVAAYMVDAP	100	SRLREVAEKRGVAAYMVDAP
26	BPSL0919 [251-270]	GVAAYMVDAPEQIDPAWVAG	100	GVAAYMVDAPEQIDPAWVAG	100	GVAAYMVDAPEQIDPAWVAG
27	BPSL0919 [261-280]	EQIDPAWVAGKRRIGVTAGA	100	EQIDPAWVAGKRRIGVTAGA	100	EQIDPAWVAGKRRIGVTAGA
28	BPSL0919 [271-290]	KRRIGVTAGASAPEVLAQAV	100	KRRIGVTAGASAPEVLAQAV	100	KRRIGVTAGASAPEVLAQAV
29	BPSL0919 [281-300]	SAPEVLAQAVIARLRELGVT	100	SAPEVLAQAVIARLRELGVT	100	SAPEVLAQAVIARLRELGVT
30	BPSL0919 [291-310]	IARLRELGVTNVRALEGIEE	100	IARLRELGVTNVRALEGIEE	100	IARLRELGVTNVRALEGIEE
31	BPSL0919 [301-320]	NVRALEGIEENVSFPLPRGL	100	NVRALEGIEENVSFPLPRGL	100	NVRALEGIEENVSFPLPRGL
32	BPSL0919 [311-326]	NVSFPLPRGLNLSSAA	100	NVSFPLPRGLNLSSAA	100	NVSFPLPRGLNLSSAA
33	BPSL0919 [307-326]	GIEENVSFPLPRGLNLSSAA	100	GIEENVSFPLPRGLNLSSAA	100	GIEENVSFPLPRGLNLSSAA

BPSL0999

Peptide No.	Antigen BPSL0999 Peptide	*Burkholderia pseudomallei*	*Burkholderia thailandensis* % homology	*Burkholderia thailandensis* Sequence	*Burkholderia mallei* % homology	*Burkholderia mallei* Sequence
1	BPSL0999 [1-20]	MNTKIATRLSVFALAGALLA	100	MNTKIATRLSVFALAGALLA	55	**M**SVFALAGALLA
2	BPSL0999 [11-30]	VFALAGALLAGCATQQGTNT	100	VFALAGALLAGCATQQGTNT	100	VFALAGALLAGCATQQGTNT
3	BPSL0999 [21-40]	GCATQQGTNTAVGTGTGAAL	100	GCATQQGTNTAVGTGTGAAL	100	GCATQQGTNTAVGTGTGAAL
4	BPSL0999 [31-50]	AVGTGTGAALGAGIGALAGG	100	AVGTGTGAALGAGIGALAGG	100	AVGTGTGAALGAGIGALAGG
5	BPSL0999 [41-60]	GAGIGALAGGGKGAAIGAGV	100	GAGIGALAGGGKGAAIGAGV	100	GAGIGALAGGGKGAAIGAGV
6	BPSL0999 [51-70]	GKGAAIGAGVGALVGGVTGY	100	GKGAAIGAGVGALVGGVTGY	100	GKGAAIGAGVGALVGGVTGY
7	BPSL0999 [61-80]	GALVGGVTGYNWQAIKNKLA	100	GALVGGVTGYNWQAIKNKLA	100	GALVGGVTGYNWQAIKNKLA
8	BPSL0999 [71-90]	NWQAIKNKLAPSAQQTGTQV	100	NWQAIKNKLAPSAQQTGTQV	100	NWQAIKNKLAPSAQQTGTQV
9	BPSL0999 [81-100]	PSAQQTGTQVTEQPDGSLKL	100	PSAQQTGTQVTEQPDGSLKL	100	PSAQQTGTQVTEQPDGSLKL
10	BPSL0999 [91-110]	TEQPDGSLKLNVPSSVTFAT	100	TEQPDGSLKLNVPSSVTFAT	100	TEQPDGSLKLNVPSSVTFAT
11	BPSL0999 [101-120]	NVPSSVTFATDQYAITPAFT	95	NVPSSVTFATDQYA**V**TPAFT	100	NVPSSVTFATDQYAITPAFT
12	BPSL0999 [111-130]	DQYAITPAFTPLLNDLATTL	95	DQYA**V**TPAFTPLLNDLATTL	100	DQYAITPAFTPLLNDLATTL
13	BPSL0999 [121-140]	PLLNDLATTLNQNPQITASV	100	PLLNDLATTLNQNPQITASV	100	PLLNDLATTLNQNPQITASV
14	BPSL0999 [131-150]	NQNPQITASVVGYTDSTGSA	100	NQNPQITASVVGYTDSTGSA	100	NQNPQITASVVGYTDSTGSA
15	BPSL0999 [141-160]	VGYTDSTGSAAHNQTLSQNR	100	VGYTDSTGSAAHNQTLSQNR	100	VGYTDSTGSAAHNQTLSQNR
16	BPSL0999 [151-170]	AHNQTLSQNRAQSVVNALAQ	100	AHNQTLSQNRAQSVVNALAQ	100	AHNQTLSQNRAQSVVNALAQ
17	BPSL0999 [161-180]	AQSVVNALAQRGVAANRLSA	95	AQSVVNALAQRGVAA**T**RLSA	100	AQSVVNALAQRGVAANRLSA
18	BPSL0999 [171-190]	RGVAANRLSAQGMGASNPIA	95	RGVAA**T**RLSAQGMGASNPIA	100	RGVAANRLSAQGMGASNPIA
19	BPSL0999 [181-200]	QGMGASNPIADNATEAGRAQ	100	QGMGASNPIADNATEAGRAQ	100	QGMGASNPIADNATEAGRAQ
20	BPSL0999 [191-210]	DNATEAGRAQNRRVEIYLRA	100	DNATEAGRAQNRRVEIYLRA	100	DNATEAGRAQNRRVEIYLRA
21	BPSL0999 [201-215]	NRRVEIYLRAPQAAQ	100	NRRVEIYLRAPQAAQ	100	NRRVEIYLRAPQAAQ

BPSL1445

Peptide No.	Antigen BPSL1445 Peptide	*Burkholderia pseudomallei*	*Burkholderia thailandensis* % homology	*Burkholderia thailandensis* Sequence	*Burkholderia mallei* % homology	*Burkholderia mallei* Sequence
1	BPSL1445 [1-20]	MHKRNLVLKAAAALVVGSLA	100	MHKRNLVLKAAAALVVGSLA	50	MRKTLVMKVAIATVLGSLA
2	BPSL1445 [11-30]	AAALVVGSLALAGCTTTPDK	100	AAALVVGSLALAGCTTTPDK	85	AIATVLGSLALAGCTTTPDK
3	BPSL1445 [21-40]	LAGCTTTPDKPANAATNASK	100	LAGCTTTPDKPANAATNASK	85	LAGCTTTPDKPANASSNAST
4	BPSL1445 [31-50]	PANAATNASKRQAIDASVDA	100	PANAATNASKRQAIDASVDA	70	PANASSNASTREAIDARVNA
5	BPSL1445 [41-60]	RQAIDASVDATLSRLYSTVR	90	RQAIDASVDATLSRMYSTVP	80	REAIDARVNATLSRLYSTVP
6	BPSL1445 [51-70]	TLSRLYSTVRGSRELVAKSR	90	TLSRMYSTVPGSRELVAKSR	90	TLSRLYSTVPGSRELVSKSR
7	BPSL1445 [61-80]	GSRELVAKSRGVLVFPDVIQ	100	GSRELVAKSRGVLVFPDVIQ	85	GSRELVSKSRGVLVFPNVLQ
8	BPSL1445 [71-90]	GVLVFPDVIQAGLIIGGQTG	100	GVLVFPDVIQAGLIIGGQTG	75	GVLVFPNVLQAGFIVGGQSG
9	BPSL1445 [81-100]	AGLIIGGQTGNGALRVGGAT	100	AGLIIGGQTGNGALRVGGAT	80	AGFIVGGQSGNGALRVGGST
10	BPSL1445 [91-110]	NGALRVGGATVGYYNTSSLS	100	NGALRVGGATVGYYNTSSLS	90	NGALRVGGSTLGYYNTSSLS
11	BPSL1445 [101-120]	VGYYNTSSLSVGLQAGAQSK	100	VGYYNTSSLSVGLQAGAQSK	95	LGYYNTSSLSVGLQAGAQSK
12	BPSL1445 [111-130]	VGLQAGAQSKAIVFLFMTQD	100	VGLQAGAQSKAIVFLFMTQD	85	VGLQAGAQSKALIFLFMSQD
13	BPSL1445 [121-140]	AIVFLFMTQDALDKFRNSDG	95	AIVFLFMTQDALDKFRGSDG	85	ALIFLFMSQDALDKFRNSDG
14	BPSL1445 [131-150]	ALDKFRNSDGWAAGADASVA	95	ALDKFRGSDGWAAGADASVA	100	ALDKFRNSDGWAAGADASVA
15	BPSL1445 [141-160]	WAAGADASVALVKMGANGAI	100	WAAGADASVALVKMGANGAI	90	WAAGADASVALVKVGANGAV
16	BPSL1445 [151-170]	LVKMGANGAIDTTTATAPVE	100	LVKMGANGAIDTTTATAPVE	80	LVKVGANGAVDTSTVTAPVE
17	BPSL1445 [161-180]	DTTTATAPVEVIVLTNAGLM	100	DTTTATAPVEVIVLTNAGLM	90	DTSTVTAPVEVIVLTNAGLM
18	BPSL1445 [171-195]	VIVLTNAGLMGDVSISGTKVTKLKI	100	VIVLTNAGLMGDVSISGTKVTKLKI	52	VIVLTNAGLMGDLSVN

BPSL2096

Peptide No.	Antigen BPSL2096 Peptide	*Burkholderia pseudomallei*	*Burkholderia thailandensis* % homology	*Burkholderia thailandensis* Sequence	*Burkholderia mallei* % homology	*Burkholderia mallei* Sequence
1	BPSL2096 [1-20]	MKTVGDKLEAFTVVAAKPGF	100	MKTVGDKLEAFTVVAAKPGF	0	MKTVGDKLEAFTVVAAKPGF
2	BPSL2096 [11-30]	FTVVAAKPGFNNHEENGQSA	100	FTVVAAKPGFNNHEENGQSA	80	**M**AAKPGFNNHEENGQSA
3	BPSL2096 [21-40]	NNHEENGQSAFETVTEASFP	100	NNHEENGQSAFETVTEASFP	100	NNHEENGQSAFETVTEASFP
4	BPSL2096 [31-50]	FETVTEASFPGKWKIIYFYP	100	FETVTEASFPGKWKIIYFYP	100	FETVTEASFPGKWKIIYFYP
5	BPSL2096 [41-60]	GKWKIIYFYPKDFTFVCPTE	100	GKWKIIYFYPKDFTFVCPTE	100	GKWKIIYFYPKDFTFVCPTE
6	BPSL20961 [51-70]	KDFTFVCPTEIVEFAKLAKQ	100	KDFTFVCPTEIVEFAKLAKQ	100	KDFTFVCPTEIVEFAKLAKQ
7	BPSL20961 [61-80]	IVEFAKLAKQFEERDAVLLG	95	IVEFAKLAKQFEERDA**I**LLG	100	IVEFAKLAKQFEERDAVLLG
8	BPSL20961 [71-90]	FEERDAVLLGGSSDNEFVKL	95	FEERDA**I**LLGGSSDNEFVKL	100	FEERDAVLLGGSSDNEFVKL
9	BPSL20961 [81-100]	GSSDNEFVKLAWRREHKDLD	100	GSSDNEFVKLAWRREHKDLD	100	GSSDNEFVKLAWRREHKDLD
10	BPSL20961 [91-110]	AWRREHKDLDKLNHYSFGDV	100	AWRREHKDLDKLNHYSFGDV	100	AWRREHKDLDKLNHYSFGDV
11	BPSL20961 [101-120]	KLNHYSFGDVKGELIDQLGV	100	KLNHYSFGDVKGELIDQLGV	100	KLNHYSFGDVKGELIDQLGV
12	BPSL20961 [111-130]	KGELIDQLGVRDKEAGVALR	100	KGELIDQLGVRDKEAGVALR	100	KGELIDQLGVRDKEAGVALR
13	BPSL2096 [121-140]	RDKEAGVALRATFIVDPDNT	100	RDKEAGVALRATFIVDPDNT	100	RDKEAGVALRATFIVDPDNT
14	BPSL2096 [131-150]	ATFIVDPDNTIQHVSVNNLN	100	ATFIVDPDNTIQHVSVNNLN	100	ATFIVDPDNTIQHVSVNNLN
15	BPSL2096 [141-160]	IQHVSVNNLNVGRSPEEILR	100	IQHVSVNNLNVGRSPEEILR	100	IQHVSVNNLNVGRSPEEILR
16	BPSL2096 [151-170]	VGRSPEEILRILDGLQTDEL	100	VGRSPEEILRILDGLQTDEL	100	VGRSPEEILRILDGLQTDEL
17	BPSL2096[161-182]	ILDGLQTDELCPCNRAIGGATL	100	ILDGLQTDELCPCNRAIGGATL	100	ILDGLQTDELCPCNRAIGGATL

BPSL2504

	Antigen BPSL2504	*Burkholderia pseudomallei*	*Burkholderia thailandensis*		*Burkholderia mallei*	
**Peptide No.**	**Peptide**		**% homology**	**Sequence**	**% homology**	**Sequence**
1	BPSL2504 [1-20]	MRTADGLELACYRWPSTAPS	75	MRTADGLELA**S**YRWP**AAGL**S	90	**V**RTAD**R**LELACYRWPSTAPS
2	BPSL2504 [11-30]	CYRWPSTAPSCAAPRATVAL	70	**S**YRWP**AAGL**S**P**AAPRATVAL	100	CYRWPSTAPSCAAPRATVAL
3	BPSL2504 [21-40]	CAAPRATVALVHGLAEHAGR	95	**P**AAPRATVALVHGLAEHAGR	100	CAAPRATVALVHGLAEHAGR
4	BPSL2504 [31-50]	VHGLAEHAGRYQAFAERLNA	95	VHGLAEHAGRYQA**L**AERLNA	100	VHGLAEHAGRYQAFAERLNA
5	BPSL2504 [41-60]	YQAFAERLNAAGIEVVAIDL	95	YQA**L**AERLNAAGIEVVAIDL	100	YQAFAERLNAAGIEVVAIDL
6	BPSL2504 [51-70]	AGIEVVAIDLRGHGRSPGER	95	AGIEVVAIDLRGHG**H**SPGER	100	AGIEVVAIDLRGHGRSPGER
7	BPSL2504 [61-80]	RGHGRSPGERAWAERFDRYL	85	RGHG**H**SPGERAW**V**ERFD**Q**YL	100	RGHGRSPGERAWAERFDRYL
8	BPSL2504 [71-90]	AWAERFDRYLDDADALVASA	80	AW**V**ERFD**Q**YL**E**DADALVAS**V**	100	AWAERFDRYLDDADALVASA
9	BPSL2504 [81-100]	DDADALVASAARENTPLFLM	80	**E**DADALVAS**V**AR**DD**TPLFLM	100	DDADALVASAARENTPLFLM
10	BPSL2504 [91-110]	ARENTPLFLMGHSMGGAIAA	85	AR**DD**TPLFLMGHSMGGA**V**AA	100	ARENTPLFLMGHSMGGAIAA
11	BPSL2504 [101-120]	GHSMGGAIAALYAIERAAAR	85	GHSMGGA**V**AALYA**V**ERAA**V**R	100	GHSMGGAIAALYAIERAAAR
12	BPSL2504 [111-130]	LYAIERAAARHASLAGLILS	70	LYA**V**ERAA**V**R**RPG**L**T**GLILS	95	LYAIERAAARHA**N**LAGLILS
13	BPSL2504 [121-140]	HASLAGLILSSPALAPGRDV	80	**RPG**L**T**GLILSSPALAPGRDV	95	HA**N**LAGLILSSPALAPGRDV
14	BPSL2504 [131-150]	SPALAPGRDVPQWMLAMSRF	95	SPALAPGRDVP**R**WMLAMSRF	100	SPALAPGRDVPQWMLAMSRF
15	BPSL2504 [141-160]	PQWMLAMSRFISRVWPRFPA	95	P**R**WMLAMSRFISRVWPRFPA	100	PQWMLAMSRFISRVWPRFPA
16	BPSL2504 [151-170]	ISRVWPRFPALKIDAALLSR	95	ISRVWPRFPA**I**KIDAALLSR	100	ISRVWPRFPALKIDAALLSR
17	BPSL2504 [161-180]	LKIDAALLSRDPAVVAANRA	95	**I**KIDAALLSRDPAVVAANRA	100	LKIDAALLSRDPAVVAANRA
18	BPSL2504 [171-190]	DPAVVAANRADPLVHHGSVP	95	DPAVVAANRADPLVHHG**P**VP	100	DPAVVAANRADPLVHHGSVP
19	BPSL2504 [181-200]	DPLVHHGSVPARTGAEILDA	95	DPLVHHG**P**VPARTGAEILDA	100	DPLVHHGSVPARTGAEILDA
20	BPSL2504 [191-210]	ARTGAEILDAMRRIEAGRAA	100	ARTGAEILDAMRRIEAGRAA	95	ARTGAEILDAMRRI**A**AGRAA
21	BPSL2504 [201-220]	MRRIEAGRAALRLPVLVYHG	95	MRRIEAGRAALR**V**PVLVYHG	90	MRRI**A**AGRAALR**I**PVLVYHG
22	BPSL2504 [211-230]	LRLPVLVYHGTADKLTEPDG	95	LR**V**PVLVYHGTADKLTEPDG	95	LR**I**PVLVYHGTADKLTEPDG
23	BPSL2504 [221-240]	TADKLTEPDGSRDFGAHVGS	95	TADKLTEPDGSRDFG**R**HVGS	100	TADKLTEPDGSRDFGAHVGS
24	BPSL2504 [231-250]	SRDFGAHVGSPDRTLTLYEG	95	SRDFG**R**HVGSPDRTLTLYEG	100	SRDFGAHVGSPDRTLTLYEG
25	BPSL2504 [241-260]	PDRTLTLYEGNYHETMNDLE	100	PDRTLTLYEGNYHETMNDLE	100	PDRTLTLYEGNYHETMNDLE
26	BPSL2504 [251-270]	NYHETMNDLERERVIGALID	100	NYHETMNDLERERVIGALID	100	NYHETMNDLERERVIGALID
27	BPSL2504 [261-280]	RERVIGALIDWIAARVPARG	95	RERVIGALIDWIAAR**A**PARG	100	RERVIGALIDWIAARVPARG

BPSL2520

	Antigen BPSL2520	*Burkholderia pseudomallei*	*Burkholderia thailandensis*		*Burkholderia mallei*	
Peptide No.	Peptide		% homology	Sequence	% homology	Sequence
1	BPSL2520 [1-20]	MQKRFKQLVLLAAMVPAFAM	100	MQKRFKQLVLLAAMVPAFAM	100	MQKRFKQLVLLAAMVPAFAM
2	BPSL2520 [11-30]	LAAMVPAFAMAQSLSNQTSA	90	LAAMVPAFAMAQSLSNQ**SA**A	100	LAAMVPAFAMAQSLSNQTSA
3	BPSL2520 [21-40]	AQSLSNQTSAPAAAAPIDAD	90	AQSLSNQ**SA**APAAAAPIDAD	100	AQSLSNQTSAPAAAAPIDAD
4	BPSL2520 [31-50]	PAAAAPIDADKKAAIKDLLD	100	PAAAAPIDADKKAAIKDLLD	100	PAAAAPIDADKKAAIKDLLD
5	BPSL2520 [41-60]	KKAAIKDLLDAIDAPKLVSA	100	KKAAIKDLLDAIDAPKLVSA	100	KKAAIKDLLDAIDAPKLVSA
6	BPSL2520 [51-70]	AIDAPKLVSAIANSAEMQSK	100	AIDAPKLVSAIANSAEMQSK	100	AIDAPKLVSAIANSAEMQSK
7	BPSL2520 [61-80]	IANSAEMQSKQLVPAILSDA	100	IANSAEMQSKQLVPAILSDA	100	IANSAEMQSKQLVPAILSDA
8	BPSL2520 [71-90]	QLVPAILSDALSENKTLNDK	100	QLVPAILSDALSENKTLNDK	100	QLVPAILSDALSENKTLNDK
9	BPSL2520 [81-100]	LSENKTLNDKQKQAAVPTLQ	100	LSENKTLNDKQKQAAVPTLQ	100	LSENKTLNDKQKQAAVPTLQ
10	BPSL2520 [91-110]	QKQAAVPTLQKNAVPKLVDG	100	QKQAAVPTLQKNAVPKLVDG	100	QKQAAVPTLQKNAVPKLVDG
11	BPSL2520 [101-120]	KNAVPKLVDGAGKVFGTQQF	100	KNAVPKLVDGAGKVFGTQQF	100	KNAVPKLVDGAGKVFGTQQF
12	BPSL2520 [111-130]	AGKVFGTQQFTNDAMQAQYD	100	AGKVFGTQQFTNDAMQAQYD	100	AGKVFGTQQFTNDAMQAQYD
13	BPSL2520 [121-140]	TNDAMQAQYDAYAKYYSTSE	100	TNDAMQAQYDAYAKYYSTSE	100	TNDAMQAQYDAYAKYYSTSE
14	BPSL2520 [131-150]	AYAKYYSTSEIKDLTTFYKS	100	AYAKYYSTSEIKDLTTFYKS	100	AYAKYYSTSEIKDLTTFYKS
15	BPSL2520 [141-160]	IKDLTTFYKSPTGRKFIQVQ	100	IKDLTTFYKSPTGRKFIQVQ	100	IKDLTTFYKSPTGRKFIQVQ
16	BPSL2520 [151-170]	PTGRKFIQVQDQVGRDVVNG	100	PTGRKFIQVQDQVGRDVVNG	100	PTGRKFIQVQDQVGRDVVNG
17	BPSL2520 [161-180]	DQVGRDVVNGLMQKYMPQAI	100	DQVGRDVVNGLMQKYMPQAI	100	DQVGRDVVNGLMQKYMPQAI
18	BPSL2520 [171-190]	LMQKYMPQAIKATRDQADKE	100	LMQKYMPQAIKATRDQADKE	100	LMQKYMPQAIKATRDQADKE
19	BPSL2520 [181-198]	KATRDQADKEVAAVKPGK	100	KATRDQADKEVAAVKPGK	100	KATRDQADKEVAAVKPGK

BPSL2522

	Antigen BPSL2522	*Burkholderia pseudomallei*	*Burkholderia thailandensis*		*Burkholderia mallei*	
Peptide No.	Peptide		% homology	Sequence	% homology	Sequence
1	BPSL2522 [1-20]	MNKLSKLAFIAATAVMAASA	100	MNKLSKLAFIAATAVMAASA	0	MNKLSKLAFIAATAVMAASA
2	BPSL2522 [11-30]	AATAVMAASASAQSVPASRQ	100	AATAVMAASASAQSVPASRQ	0	AATAVMAASASAQSVPASRQ
3	BPSL2522 [21-40]	SAQSVPASRQAVNDNWVNGT	100	SAQSVPASRQAVNDNWVNGT	75	**M**PASRQAVNDNWVNGT
4	BPSL2522 [31-50]	AVNDNWVNGTGEWVWMNGTN	100	AVNDNWVNGTGEWVWMNGTN	100	AVNDNWVNGTGEWVWMNGTN
5	BPSL2522 [41-60]	GEWVWMNGTNELCWRDAFWT	100	GEWVWMNGTNELCWRDAFWT	100	GEWVWMNGTNELCWRDAFWT
6	BPSL2522 [51-70]	ELCWRDAFWTPATANAKCDG	100	ELCWRDAFWTPATANAKCDG	100	ELCWRDAFWTPATANAKCDG
7	BPSL2522 [61-80]	PATANAKCDGALVAQAPAPA	100	PATANAKCDGALVAQAPAPA	100	PATANAKCDGALVAQAPAPA
8	BPSL2522 [71-90]	ALVAQAPAPAPVAPVAPAIT	100	ALVAQAPAPAPVAPVAPAIT	100	ALVAQAPAPAPVAPVAPAIT
9	BPSL2522 [81-100]	PVAPVAPAITSQKITYQADT	100	PVAPVAPAITSQKITYQADT	100	PVAPVAPAITSQKITYQADT
10	BPSL2522 [91-110]	SQKITYQADTLFDFDKAVLK	95	SQKITYQADTLFDFDKA**I**LK	100	SQKITYQADTLFDFDKAVLK
11	BPSL2522 [101-120]	LFDFDKAVLKPAGKQKLDEL	95	LFDFDKA**I**LKPAGKQKLDEL	100	LFDFDKAVLKPAGKQKLDEL
12	BPSL2522 [111-130]	PAGKQKLDELAAKIQGMNVE	100	PAGKQKLDELAAKIQGMNVE	100	PAGKQKLDELAAKIQGMNVE
13	BPSL2522 [121-140]	AAKIQGMNVEVVVATGYTDR	100	AAKIQGMNVEVVVATGYTDR	100	AAKIQGMNVEVVVATGYTDR
14	BPSL2522 [131-150]	VVVATGYTDRIGSDKYNDRL	100	VVVATGYTDRIGSDKYNDRL	100	VVVATGYTDRIGSDKYNDRL
15	BPSL2522 [141-160]	IGSDKYNDRLSLRRAQAVKS	100	IGSDKYNDRLSLRRAQAVKS	100	IGSDKYNDRLSLRRAQAVKS
16	BPSL2522 [151-170]	SLRRAQAVKSYLVSKGVPAN	100	SLRRAQAVKSYLVSKGVPAN	100	SLRRAQAVKSYLVSKGVPAN
17	BPSL2522 [161-180]	YLVSKGVPANKVYTEGKGKR	100	YLVSKGVPANKVYTEGKGKR	100	YLVSKGVPANKVYTEGKGKR
18	BPSL2522 [171-190]	KVYTEGKGKRNPVTGNTCKQ	95	KVYTEGKGKRNP**I**TGNTCKQ	100	KVYTEGKGKRNPVTGNTCKQ
19	BPSL2522 [181-200]	NPVTGNTCKQKNRKQLIACL	95	NP**I**TGNTCKQKNRKQLIACL	100	NPVTGNTCKQKNRKQLIACL
20	BPSL2522 [191-210]	KNRKQLIACLAPDRRVEVEV	100	KNRKQLIACLAPDRRVEVEV	100	KNRKQLIACLAPDRRVEVEV
21	BPSL2522 [201-220]	APDRRVEVEVVGTQEVQKTT	100	APDRRVEVEVVGTQEVQKTT	100	APDRRVEVEVVGTQEVQKTT
22	BPSL2522 [211-224]]	VGTQEVQKTTVPAQ	93	VGTQEVQKTTVPA**K**	100	VGTQEVQKTTVPAQ
23	BPSL2522 [205-224]	RVEVEVVGTQEVQKTTVPAQ	95	RVEVEVVGTQEVQKTTVPA**K**	100	RVEVEVVGTQEVQKTTVPAQ

BPSL2697

Peptide No.	Antigen BPSL2697 Peptide	*Burkholderia pseudomallei*	*Burkholderia thailandensis* % homology	*Burkholderia thailandensis* Sequence	*Burkholderia mallei* % homology	*Burkholderia mallei* Sequence
1	BPSL2697 [1-20]	MAAKDVVFGDSARAKMVEGV	100	MAAKDVVFGDSARAKMVEGV	100	MAAKDVVFGDSARAKMVEGV
2	BPSL2697 [11-30]	SARAKMVEGVNILANAVKVT	100	SARAKMVEGVNILANAVKVT	100	SARAKMVEGVNILANAVKVT
3	BPSL2697 [21-40]	NILANAVKVTLGPKGRNVVL	100	NILANAVKVTLGPKGRNVVL	100	NILANAVKVTLGPKGRNVVL
4	BPSL2697 [31-50]	LGPKGRNVVLERSFGGPTVT	100	LGPKGRNVVLERSFGGPTVT	100	LGPKGRNVVLERSFGGPTVT
5	BPSL2697 [41-60]	ERSFGGPTVTKDGVSVAKEI	100	ERSFGGPTVTKDGVSVAKEI	100	ERSFGGPTVTKDGVSVAKEI
6	BPSL2697 [51-70]	KDGVSVAKEIELKDKLQNMG	100	KDGVSVAKEIELKDKLQNMG	100	KDGVSVAKEIELKDKLQNMG
7	BPSL2697 [61-80]	ELKDKLQNMGAQMVKEVASK	100	ELKDKLQNMGAQMVKEVASK	100	ELKDKLQNMGAQMVKEVASK
8	BPSL2697 [71-90]	AQMVKEVASKTSDNAGDGTT	100	AQMVKEVASKTSDNAGDGTT	100	AQMVKEVASKTSDNAGDGTT
9	BPSL2697 [81-100]	TSDNAGDGTTTATVLAQSIV	100	TSDNAGDGTTTATVLAQSIV	100	TSDNAGDGTTTATVLAQSIV
10	BPSL2697 [91-110]	TATVLAQSIVREGMKYVASG	100	TATVLAQSIVREGMKYVASG	100	TATVLAQSIVREGMKYVASG
11	BPSL2697 [101-120]	REGMKYVASGMNPMDLKRGI	100	REGMKYVASGMNPMDLKRGI	100	REGMKYVASGMNPMDLKRGI
12	BPSL2697 [111-130]	MNPMDLKRGIDKAVAAAVEE	100	MNPMDLKRGIDKAVAAAVEE	100	MNPMDLKRGIDKAVAAAVEE
13	BPSL2697 [121-140]	DKAVAAAVEELKKISKPCTT	100	DKAVAAAVEELKKISKPCTT	100	DKAVAAAVEELKKISKPCTT
14	BPSL2697 [131-150]	LKKISKPCTTNKEIAQVGAI	95	LKKISKPCTTNKEIAQVG**S**I	100	LKKISKPCTTNKEIAQVGAI
15	BPSL2697 [141-160]	NKEIAQVGAISANSDSSIGD	95	NKEIAQVG**S**ISANSDSSIGD	100	NKEIAQVGAISANSDSSIGD
16	BPSL2697 [151-170]	SANSDSSIGDRIAEAMDKVG	100	SANSDSSIGDRIAEAMDKVG	100	SANSDSSIGDRIAEAMDKVG
17	BPSL2697 [161-180]	RIAEAMDKVGKEGVITVEDG	100	RIAEAMDKVGKEGVITVEDG	100	RIAEAMDKVGKEGVITVEDG
18	BPSL2697 [171-190]	KEGVITVEDGKSLADELDVV	100	KEGVITVEDGKSLADELDVV	100	KEGVITVEDGKSLADELDVV
19	BPSL2697 [181-200]	KSLADELDVVEGMQFDRGYL	100	KSLADELDVVEGMQFDRGYL	100	KSLADELDVVEGMQFDRGYL
20	BPSL2697 [191-210]	EGMQFDRGYLSPYFINNPDK	100	EGMQFDRGYLSPYFINNPDK	100	EGMQFDRGYLSPYFINNPDK
21	BPSL2697 [201-220]	SPYFINNPDKQVAVLENPFV	100	SPYFINNPDKQVAVLENPFV	100	SPYFINNPDKQVAVLENPFV
22	BPSL2697 [211-230]	QVAVLENPFVLLHDKKVSNI	100	QVAVLENPFVLLHDKKVSNI	100	QVAVLENPFVLLHDKKVSNI
23	BPSL2697 [221-240]	LLHDKKVSNIRDLLPVLEQV	100	LLHDKKVSNIRDLLPVLEQV	100	LLHDKKVSNIRDLLPVLEQV
24	BPSL2697 [231-250]	RDLLPVLEQVAKAGRPLLII	100	RDLLPVLEQVAKAGRPLLII	100	RDLLPVLEQVAKAGRPLLII
25	BPSL2697 [241-260]	AKAGRPLLIIAEDVEGEALA	100	AKAGRPLLIIAEDVEGEALA	100	AKAGRPLLIIAEDVEGEALA
26	BPSL2697 [251-270]	AEDVEGEALATLVVNNIRGI	100	AEDVEGEALATLVVNNIRGI	100	AEDVEGEALATLVVNNIRGI
27	BPSL2697 [261-280]	TLVVNNIRGILKTVAVKAPG	100	TLVVNNIRGILKTVAVKAPG	100	TLVVNNIRGILKTVAVKAPG
28	BPSL2697 [271-290]	LKTVAVKAPGFGDRRKAMLE	100	LKTVAVKAPGFGDRRKAMLE	100	LKTVAVKAPGFGDRRKAMLE
29	BPSL2697 [281-300]	FGDRRKAMLEDIAILTGGQV	100	FGDRRKAMLEDIAILTGGQV	100	FGDRRKAMLEDIAILTGGQV
30	BPSL2697 [291-310]	DIAILTGGQVIAEETGLTLE	100	DIAILTGGQVIAEETGLTLE	100	DIAILTGGQVIAEETGLTLE
31	BPSL2697 [301-320]	IAEETGLTLEKATLAELGQA	100	IAEETGLTLEKATLAELGQA	100	IAEETGLTLEKATLAELGQA
32	BPSL2697 [311-330]	KATLAELGQAKRIEVGKENT	100	KATLAELGQAKRIEVGKENT	100	KATLAELGQAKRIEVGKENT
33	BPSL2697 [321-340]	KRIEVGKENTTIIDGAGEAV	100	KRIEVGKENTTIIDGAGEAV	100	KRIEVGKENTTIIDGAGEAV
34	BPSL2697 [331-350]	TIIDGAGEAVNIEARVKQIR	100	TIIDGAGEAVNIEARVKQIR	100	TIIDGAGEAVNIEARVKQIR
35	BPSL2697 [341-360]	NIEARVKQIRTQIEEATSDY	100	NIEARVKQIRTQIEEATSDY	100	NIEARVKQIRTQIEEATSDY
36	BPSL2697 [351-370]	TQIEEATSDYDREKLQERVA	100	TQIEEATSDYDREKLQERVA	100	TQIEEATSDYDREKLQERVA
37	BPSL2697 [361-380]	DREKLQERVAKLAGGVAVIK	100	DREKLQERVAKLAGGVAVIK	100	DREKLQERVAKLAGGVAVIK
38	BPSL2697 [371-390]	KLAGGVAVIKVGAATEVEMK	100	KLAGGVAVIKVGAATEVEMK	100	KLAGGVAVIKVGAATEVEMK
39	BPSL2697 [381-400]	VGAATEVEMKEKKARVEDAL	100	VGAATEVEMKEKKARVEDAL	100	VGAATEVEMKEKKARVEDAL
40	BPSL2697 [391-410]	EKKARVEDALHATRAAVEEG	100	EKKARVEDALHATRAAVEEG	100	EKKARVEDALHATRAAVEEG
41	BPSL2697 [401-420]	HATRAAVEEGIVPGGGVALI	100	HATRAAVEEGIVPGGGVALI	100	HATRAAVEEGIVPGGGVALI
42	BPSL2697 [411-430]	IVPGGGVALIRARTAIAGLT	95	IVPGGGVALIRARTAIA**A**LT	95	IVPGGGVALIRARTAIA**S**LT
43	BPSL2697 [421-440]	RARTAIAGLTGVNADQNAGI	95	RARTAIA**A**LTGVNADQNAGI	95	RARTAIA**S**LTGVNADQNAGI
44	BPSL2697 [431-450]	GVNADQNAGIKIVLRAMEEP	100	GVNADQNAGIKIVLRAMEEP	100	GVNADQNAGIKIVLRAMEEP
45	BPSL2697 [441-460]	KIVLRAMEEPLRQIVTNGGE	100	KIVLRAMEEPLRQIVTNGGE	100	KIVLRAMEEPLRQIVTNGGE
46	BPSL2697 [451-470]	LRQIVTNGGEEASVVVAAVA	100	LRQIVTNGGEEASVVVAAVA	100	LRQIVTNGGEEASVVVAAVA
47	BPSL2697 [461-480]	EASVVVAAVAAGKGNYGYNA	100	EASVVVAAVAAGKGNYGYNA	100	EASVVVAAVAAGKGNYGYNA
48	BPSL2697 [471-490]	AGKGNYGYNAATGEYVDMVE	100	AGKGNYGYNAATGEYVDMVE	100	AGKGNYGYNAATGEYVDMVE
49	BPSL2697 [481-500]	ATGEYVDMVEAGVVDPTKVT	100	ATGEYVDMVEAGVVDPTKVT	100	ATGEYVDMVEAGVVDPTKVT
50	BPSL2697 [491-510]	AGVVDPTKVTRTALQNAASV	100	AGVVDPTKVTRTALQNAASV	100	AGVVDPTKVTRTALQNAASV
51	BPSL2697 [501-520]	RTALQNAASVAGLLLTTDAA	100	RTALQNAASVAGLLLTTDAA	100	RTALQNAASVAGLLLTTDAA
52	BPSL2697 [511-530]	AGLLLTTDAAVAELPKEDAP	100	AGLLLTTDAAVAELPKEDAP	100	AGLLLTTDAAVAELPKEDAP
53	BPSL2697 [521-540]	VAELPKEDAPMPGGMPGGMG	100	VAELPKEDAPMPGGMPGGMG	100	VAELPKEDAPMPGGMPGGMG
54	BPSL2697 [531-546]	MPGGMPGGMGGMGMDM	100	MPGGMPGGMGGMGMDM	94	MPGGMPGGMGGMGM**G**M

BPSL2765

Peptide No.	Antigen BPSL2765 Peptide	*Burkholderia pseudomallei*	*Burkholderia thailandensis* % homology	*Burkholderia thailandensis* Sequence	*Burkholderia mallei* % homology	*Burkholderia mallei* Sequence
1	BPSL2765 [1-20]	MMSKKLRLAFAMLMIGALAA	100	MMSKKLRLAFAMLMIGALAA	95	MSKKLRLAFAMLMIGALAA
2	BPSL2765 [11-30]	AMLMIGALAACKSGVKLDEH	100	AMLMIGALAACKSGVKLDEH	100	AMLMIGALAACKSGVKLDEH
3	BPSL2765 [21-40]	CKSGVKLDEHANQGDAVSTQ	95	CKSGVKLDEHANQG**G**AVSTQ	100	CKSGVKLDEHANQGDAVSTQ
4	BPSL2765 [31-50]	ANQGDAVSTQPNPENVAQVT	95	ANQG**G**AVSTQPNPENVAQVT	100	ANQGDAVSTQPNPENVAQVT
5	BPSL2765 [41-60]	PNPENVAQVTVDPLNDPNSP	100	PNPENVAQVTVDPLNDPNSP	100	PNPENVAQVTVDPLNDPNSP
6	BPSL2765 [51-70]	VDPLNDPNSPLAKRSVYFDF	100	VDPLNDPNSPLAKRSVYFDF	100	VDPLNDPNSPLAKRSVYFDF
7	BPSL2765 [61-80]	LAKRSVYFDFDSYSVQDQYQ	100	LAKRSVYFDFDSYSVQDQYQ	100	LAKRSVYFDFDSYSVQDQYQ
8	BPSL2765 [71-90]	DSYSVQDQYQALLQQHAQYL	95	DSYSVQDQYQ**P**LLQQHAQYL	100	DSYSVQDQYQALLQQHAQYL
9	BPSL2765 [81-100]	ALLQQHAQYLKSHPQRHILI	95	**P**LLQQHAQYLKSHPQRHILI	100	ALLQQHAQYLKSHPQRHILI
10	BPSL2765 [91-110]	KSHPQRHILIQGNTDERGTS	100	KSHPQRHILIQGNTDERGTS	100	KSHPQRHILIQGNTDERGTS
11	BPSL2765 [101-120]	QGNTDERGTSEYNLALGQKR	100	QGNTDERGTSEYNLALGQKR	100	QGNTDERGTSEYNLALGQKR
12	BPSL2765 [111-130]	EYNLALGQKRAEAVRRALSL	100	EYNLALGQKRAEAVRRALSL	100	EYNLALGQKRAEAVRRALSL
13	BPSL2765 [121-140]	AEAVRRALSLLGVGDAQMEA	95	AEAVRRALSLLGVGD**S**QMEA	100	AEAVRRALSLLGVGDAQMEA
14	BPSL2765 [131-150]	LGVGDAQMEAVSLGKEKPVA	95	LGVGD**S**QMEAVSLGKEKPVA	100	LGVGDAQMEAVSLGKEKPVA
15	BPSL2765 [141-160]	VSLGKEKPVALGHDEASWAQ	100	VSLGKEKPVALGHDEASWAQ	100	VSLGKEKPVALGHDEASWAQ
16	BPSL2765 [151-170]	LGHDEASWAQNRRADLVYQQ	100	LGHDEASWAQNRRADLVYQQ	100	LGHDEASWAQNRRADLVYQQ

BPSL3319

Peptide No.	Antigen BPSL3319 Peptide	*Burkholderia pseudomallei*	*Burkholderia thailandensis* % homology	*Burkholderia thailandensis* Sequence	*Burkholderia mallei* % homology	*Burkholderia mallei* Sequence
1	BPSL3319 [1-20]	MLGINSNINSLVAQQNLNGS	95	MLGINSN**L**NSLVAQQNLNGS	100	MLGINSNINSLVAQQNLNGS
2	BPSL3319 [11-30]	LVAQQNLNGSQGALSQAITR	95	LVAQQNLNGSQ**S**ALSQAITR	100	LVAQQNLNGSQGALSQAITR
3	BPSL3319 [21-40]	QGALSQAITRLSSGKRINSA	95	Q**S**ALSQAITRLSSGKRINSA	100	QGALSQAITRLSSGKRINSA
4	BPSL3319 [31-50]	LSSGKRINSAADDAAGLAIA	100	LSSGKRINSAADDAAGLAIA	100	LSSGKRINSAADDAAGLAIA
5	BPSL3319 [41-60]	ADDAAGLAIATRMQTQINGL	100	ADDAAGLAIATRMQTQINGL	100	ADDAAGLAIATRMQTQINGL
6	BPSL3319 [51-70]	TRMQTQINGLNQGVSNANDG	100	TRMQTQINGLNQGVSNANDG	100	TRMQTQINGLNQGVSNANDG
7	BPSL3319 [61-80]	NQGVSNANDGVSILQTASSG	100	NQGVSNANDGVSILQTASSG	100	NQGVSNANDGVSILQTASSG
8	BPSL3319 [71-90]	VSILQTASSGLTSLTNSLQR	100	VSILQTASSGLTSLTNSLQR	100	VSILQTASSGLTSLTNSLQR
9	BPSL3319 [81-100]	LTSLTNSLQRIRQLAVQASN	100	LTSLTNSLQRIRQLAVQASN	100	LTSLTNSLQRIRQLAVQASN
10	BPSL3319 [91-110]	IRQLAVQASNGPLSASDASA	100	IRQLAVQASNGPLSASDASA	100	IRQLAVQASNGPLSASDASA
11	BPSL3319 [101-120]	GPLSASDASALQQEVAQQIS	100	GPLSASDASALQQEVAQQIS	100	GPLSASDASALQQEVAQQIS
12	BPSL3319 [111-130]	LQQEVAQQISEVNRIASQTN	100	LQQEVAQQISEVNRIASQTN	100	LQQEVAQQISEVNRIASQTN
13	BPSL3319 [121-140]	EVNRIASQTNYNGKNILDGS	100	EVNRIASQTNYNGKNILDGS	100	EVNRIASQTNYNGKNILDGS
14	BPSL3319 [131-150]	YNGKNILDGSAGTLSFQVGA	100	YNGKNILDGSAGTLSFQVGA	100	YNGKNILDGSAGTLSFQVGA
15	BPSL3319 [141-160]	AGTLSFQVGANVGQTVSVDL	95	AGTLSFQVGANVGQTV**T**VDL	100	AGTLSFQVGANVGQTVSVDL
16	BPSL3319 [151-170]	NVGQTVSVDLTQSMSAAKIG	90	NVGQTV**T**VDL**S**QSMSAAKIG	100	NVGQTVSVDLTQSMSAAKIG
17	BPSL3319 [161-180]	TQSMSAAKIGGGMVQTGQTL	90	**S**QSMSAAKIGGG**L**VQTGQTL	100	TQSMSAAKIGGGMVQTGQTL
18	BPSL3319 [171-190]	GGMVQTGQTLGTIKVAIDSS	85	GG**L**VQTGQTLGT**F**KVA**V**DSS	100	GGMVQTGQTLGTIKVAIDSS
19	BPSL3319 [181-200]	GTIKVAIDSSGAAWSSGSTG	75	GT**F**KVA**V**DSSGAAW**TAS**STG	100	GTIKVAIDSSGAAWSSGSTG
20	BPSL3319 [191-210]	GAAWSSGSTGQETTQINVVS	80	GAAW**TAS**STGQETTQINV**L**S	100	GAAWSSGSTGQETTQINVVS
21	BPSL3319 [201-220]	QETTQINVVSDGKGGFTFTD	95	QETTQINV**L**SDGKGGFTFTD	100	QETTQINVVSDGKGGFTFTD
22	BPSL3319 [211-230]	DGKGGFTFTDQNNQALSSTA	95	DGKGGFTFTDQNNQ**T**LSSTA	100	DGKGGFTFTDQNNQALSSTA
23	BPSL3319 [221-240]	QNNQALSSTAVTAVFGSSTA	85	QNNQ**T**LSSTAVTA**L**FG**A**STA	100	QNNQALSSTAVTAVFGSSTA
24	BPSL3319 [231-250]	VTAVFGSSTAGTGTAASPSF	60	VTA**L**FG**A**STAG**S**GTA**LTVTL**	100	VTAVFGSSTAGTGTAASPSF
25	BPSL3319 [241-260]	GTGTAASPSFQTLALSTSAT	30	G**S**GTA**LTVTLNSA**ATS**SLSA**	100	GTGTAASPSFQTLALSTSAT
26	BPSL3319 [251-270]	QTLALSTSATSALSATDQAN	50	**LTVT**L**NSA**ATS**S**LSA**A**DQA**A**	100	QTLALSTSATSALSATDQAN
27	BPSL3319 [261-280]	SALSATDQANATAMVAQINA	65	S**S**LSA**A**DQA**A**A**A**AM**QT**Q**V**NA	100	SALSATDQANATAMVAQINA
28	BPSL3319 [271-290]	ATAMVAQINAVNKPQTVSNL	75	A**A**AM**QT**Q**V**NAVN**Q**PQTVSNL	100	ATAMVAQINAVNKPQTVSNL
29	BPSL3319 [281-300]	VNKPQTVSNLDISTQTGAYQ	90	VN**Q**PQTVSNL**N**ISTQTGAYQ	100	VNKPQTVSNLDISTQTGAYQ
30	BPSL3319 [291-310]	DISTQTGAYQAMVSIDNALA	95	**N**ISTQTGAYQAMVSIDNALA	100	DISTQTGAYQAMVSIDNALA
31	BPSL3319 [301-320]	AMVSIDNALATVNNLQATLG	100	AMVSIDNALATVNNLQATLG	100	AMVSIDNALATVNNLQATLG
32	BPSL3319 [311-330]	TVNNLQATLGAAQNRFTAIA	100	TVNNLQATLGAAQNRFTAIA	100	TVNNLQATLGAAQNRFTAIA
33	BPSL3319 [321-340]	AAQNRFTAIATTQQAGSNNL	100	AAQNRFTAIATTQQAGSNNL	100	AAQNRFTAIATTQQAGSNNL
34	BPSL3319 [331-350]	TTQQAGSNNLAQAQSQIQSA	100	TTQQAGSNNLAQAQSQIQSA	100	TTQQAGSNNLAQAQSQIQSA
35	BPSL3319 [341-360]	AQAQSQIQSADFAQETANLS	100	AQAQSQIQSADFAQETANLS	100	AQAQSQIQSADFAQETANLS
36	BPSL3319 [351-370]	DFAQETANLSRAQVLQQAGI	100	DFAQETANLSRAQVLQQAGI	100	DFAQETANLSRAQVLQQAGI
37	BPSL3319 [361-380]	RAQVLQQAGISVLAQANSLP	100	RAQVLQQAGISVLAQANSLP	100	RAQVLQQAGISVLAQANSLP
38	BPSL3319 [371-388]	SVLAQANSLPQQVLKLLQ	100	SVLAQANSLPQQVLKLLQ	100	SVLAQANSLPQQVLKLLQ

BPSS0477

	Antigen BPSS0477	*Burkholderia pseudomallei*	*Burkholderia thailandensis*		*Burkholderia mallei*	
**Peptide No.**	**Peptide**		**% homology**	**Sequence**	**% homology**	**Sequence**
1	BPSS0477 [1-20]	MAAKEIIFHDGARAKLVEGV	95	MAAKEIIFHDGARTKLVEGV	70	MAAKDVVFGDSARAKMVEGV
2	BPSS0477 [11-30]	GARAKLVEGVNLLANAVKVT	95	GARTKLVEGVNLLANAVKVT	85	SARAKMVEGVNILANAVKVT
3	BPSS0477 [21-40]	NLLANAVKVTLGPKGRNVVL	100	NLLANAVKVTLGPKGRNVVL	95	NILANAVKVTLGPKGRNVVL
4	BPSS0477 [31-50]	LGPKGRNVVLERSFGSPVVT	100	LGPKGRNVVLERSFGSPVVT	90	LGPKGRNVVLERSFGGPTVT
5	BPSS0477 [41-60]	ERSFGSPVVTKDGVSVAKEI	100	ERSFGSPVVTKDGVSVAKEI	90	ERSFGGPTVTKDGVSVAKEI
6	BPSS0477 [51-70]	KDGVSVAKEIELADKVQNIG	100	KDGVSVAKEIELADKVQNIG	85	KDGVSVAKEIELKDKLQNMG
7	BPSS0477 [61-80]	ELADKVQNIGAQLVKEVASK	100	ELADKVQNIGAQLVKEVASK	80	ELKDKLQNMGAQMVKEVASK
8	BPSS0477 [71-90]	AQLVKEVASKTSDAAGDGTT	100	AQLVKEVASKTSDAAGDGTT	90	AQMVKEVASKTSDNAGDGTT
9	BPSS0477 [81-100]	TSDAAGDGTTTATVLAQAIV	100	TSDAAGDGTTTATVLAQAIV	90	TSDNAGDGTTTATVLAQSIV
10	BPSS0477 [91-110]	TATVLAQAIVREGQKYVAAG	100	TATVLAQAIVREGQKYVAAG	85	TATVLAQSIVREGMKYVASG
11	BPSS0477 [101-120]	REGQKYVAAGLNPLDLKRGI	100	REGQKYVAAGLNPLDLKRGI	80	REGMKYVASGMNPMDLKRGI
12	BPSS0477 [111-130]	LNPLDLKRGIDKAVAAAVDE	95	LNPLDLKRGIDKAVAAAVEE	85	MNPMDLKRGIDKAVAAAVEE
13	BPSS0477 [121-140]	DKAVAAAVDELKKISKPTTT	95	DKAVAAAVEELKKISKPTTT	80	DKAVAAAVEELKKISKPCTT
14	BPSS0477 [131-150]	LKKISKPTTTSKEIAQVATI	100	LKKISKPTTTSKEIAQVATI	80	LKKISKPCTTNKEIAQVGAI
15	BPSS0477 [141-160]	SKEIAQVATISANGEESIGQ	100	SKEIAQVATISANGEESIGQ	65	NKEIAQVGAISANSDSSIGD
16	BPSS0477 [151-170]	SANGEESIGQRIAEAIDRVG	100	SANGEESIGQRIAEAIDRVG	70	SANSDSSIGDRIAEAMDKVG
17	BPSS0477 [161-180]	RIAEAIDRVGKEGVITVEDG	100	RIAEAIDRVGKEGVITVEDG	90	RIAEAMDKVGKEGVITVEDG
18	BPSS0477 [171-190]	KEGVITVEDGKSLADELDVV	100	KEGVITVEDGKSLADELDVV	100	KEGVITVEDGKSLADELDVV
19	BPSS0477 [181-200]	KSLADELDVVEGLQFDRGYL	100	KSLADELDVVEGLQFDRGYL	95	KSLADELDVVEGMQFDRGYL
20	BPSS0477 [191-210]	EGLQFDRGYLSPYFINHPER	90	EGLQFDRGYLSPYFINNPDR	80	EGMQFDRGYLSPYFINNPDK
21	BPSS0477 [201-220]	SPYFINHPERQLAVLDEPFI	90	SPYFINNPDRQLAVLDEPFI	65	SPYFINNPDKQVAVLENPFV
22	BPSS0477 [211-230]	QLAVLDEPFILLHDKKISNI	100	QLAVLDEPFILLHDKKISNI	75	QVAVLENPFVLLHDKKVSNI
23	BPSS0477 [221-240]	LLHDKKISNIRDLLPVLEQV	100	LLHDKKISNIRDLLPVLEQV	95	LLHDKKVSNIRDLLPVLEQV
24	BPSS0477 [231-250]	RDLLPVLEQVAKAGRPLLIV	100	RDLLPVLEQVAKAGRPLLIV	95	RDLLPVLEQVAKAGRPLLII
25	BPSS0477 [241-260]	AKAGRPLLIVAEDVEGEALA	100	AKAGRPLLIVAEDVEGEALA	95	AKAGRPLLIIAEDVEGEALA
26	BPSS0477 [251-270]	AEDVEGEALATLVVNNIRGI	95	AEDVEGEALATLVVNNIRGT	100	AEDVEGEALATLVVNNIRGI
27	BPSS0477 [261-280]	TLVVNNIRGILKTVAVKAPG	95	TLVVNNIRGTLKTVAVKAPG	100	TLVVNNIRGILKTVAVKAPG
28	BPSS0477 [271-290]	LKTVAVKAPGFGDRRKALLE	100	LKTVAVKAPGFGDRRKALLE	95	LKTVAVKAPGFGDRRKAMLE
29	BPSS0477 [281-300]	FGDRRKALLEDIAILTGGQV	100	FGDRRKALLEDIAILTGGQV	95	FGDRRKAMLEDIAILTGGQV
30	BPSS0477 [291-310]	DIAILTGGQVITEETGLTLE	95	DIAILTGGQVIAEETGLTLE	95	DIAILTGGQVIAEETGLTLE
31	BPSS0477 [301-320]	ITEETGLTLEKATLQELGRA	95	IAEETGLTLEKATLQELGRA	85	IAEETGLTLEKATLAELGQA
32	BPSS0477 [311-330]	KATLQELGRAKRIEVGKENT	100	KATLQELGRAKRIEVGKENT	90	KATLAELGQAKRIEVGKENT
33	BPSS0477 [321-340]	KRIEVGKENTTLIDGAGDKP	100	KRIEVGKENTTLIDGAGDKP	80	KRIEVGKENTTIIDGAGEAV
34	BPSS0477 [331-350]	TLIDGAGDKPNIDARVKQIR	100	TLIDGAGDKPNIDARVKQIR	75	TIIDGAGEAVNIEARVKQIR
35	BPSS0477 [341-360]	NIDARVKQIRAQIAEATSDY	95	NIDARVKQIRAQIADATSDY	85	NIEARVKQIRTQIEEATSDY
36	BPSS0477 [351-370]	AQIAEATSDYDREKLQERVA	95	AQIADATSDYDREKLQERVA	90	TQIEEATSDYDREKLQERVA
37	BPSS0477 [361-380]	DREKLQERVAKLAGGVAVIK	100	DREKLQERVAKLAGGVAVIK	100	DREKLQERVAKLAGGVAVIK
38	BPSS0477 [371-390]	KLAGGVAVIKVGGATEVEVK	100	KLAGGVAVIKVGGATEVEVK	90	KLAGGVAVIKVGAATEVEMK
39	BPSS0477 [381-400]	VGGATEVEVKEKKDRVDDAL	100	VGGATEVEVKEKKDRVDDAL	80	VGAATEVEMKEKKARVEDAL
40	BPSS0477 [391-410]	EKKDRVDDALHATRAAVEEG	100	EKKDRVDDALHATRAAVEEG	90	EKKARVEDALHATRAAVEEG
41	BPSS0477 [401-420]	HATRAAVEEGIVPGGGVALI	100	HATRAAVEEGIVPGGGVALI	100	HATRAAVEEGIVPGGGVALI
42	BPSS0477 [411-430]	IVPGGGVALIRVKQAIAALA	90	IVPGGGVALIRVKQAIAELT	75	IVPGGGVALIRARTAIASLT
43	BPSS0477 [421-440]	RVKQAIAALAGANADQKAGI	90	RVKQAIAELTGANADQKAGI	65	RARTAIASLTGVNADQNAGI
44	BPSS0477 [431-450]	GANADQKAGISIVLRALEEP	95	GANADQKAGINIVLRALEEP	80	GVNADQNAGIKIVLRAMEEP
45	BPSS0477 [441-460]	SIVLRALEEPLRQIVANAGE	95	NIVLRALEEPLRQIVANAGE	80	KIVLRAMEEPLRQIVTNGGE
46	BPSS0477 [451-470]	LRQIVANAGEEASVVVATVA	100	LRQIVANAGEEASVVVATVA	85	LRQIVTNGGEEASVVVAAVA
47	BPSS0477 [461-480]	EASVVVATVAAGQGNYGYNA	95	EASVVVATVAAGSGNYGYNA	90	EASVVVAAVAAGKGNYGYNA
48	BPSS0477 [471-490]	AGQGNYGYNAATGEYGDLVE	95	AGSGNYGYNAATGEYGDLVE	85	AGKGNYGYNAATGEYVDMVE
49	BPSS0477 [481-500]	ATGEYGDLVESGVLDPTKVT	100	ATGEYGDLVESGVLDPTKVT	80	ATGEYVDMVEAGVVDPTKVT
50	BPSS0477 [491-510]	SGVLDPTKVTRTALQNAASI	100	SGVLDPTKVTRTALQNAASI	85	AGVVDPTKVTRTALQNAASV
51	BPSS0477 [501-520]	RTALQNAASIAGLLLTTDAT	100	RTALQNAASIAGLLLTTDAT	90	RTALQNAASVAGLLLTTDAA
52	BPSS0477 [511-530]	AGLLLTTDATVHEAPKDAPP	100	AGLLLTTDATVHEAPKDAPP	70	AGLLLTTDAAVAELPKEDAP
53	BPSS0477 [521-540]	VHEAPKDAPPAAPAGVPGAG	95	VHEAPKDAPPAQPAGVPGAG	30	VAELPKEDAPMPGGMPGGMG
54	BPSS0477 [531-546]	AAPAGVPGAGGPGFDF	87	AQPAGVPGAGGTGFDF	20	MPGGMPGGMGGMGMGM

BPSS0530

Peptide No.	Antigen BPSS0530 Peptide	*Burkholderia pseudomallei*	*Burkholderia thailandensis* % homology	*Burkholderia thailandensis* Sequence	*Burkholderia mallei* % homology	*Burkholderia mallei* Sequence
1	BPSS0530 [1-20]	MSSLPVGPVAWSDGMLIETQ	100	MSSLPVGPVAWSDGMLIETQ	100	MSSLPVGPVAWSDGMLIETQ
2	BPSS0530 [11-30]	WSDGMLIETQHFQQLERHLA	100	WSDGMLIETQHFQQLERHLA	100	WSDGMLIETQHFQQLERHLA
3	BPSS0530 [21-40]	HFQQLERHLAHQASLRLGQT	95	HFQQLERHLAHQA**A**LRLGQT	100	HFQQLERHLAHQASLRLGQT
4	BPSS0530 [31-50]	HQASLRLGQTSNHGWGFTLL	95	HQA**A**LRLGQTSNHGWGFTLL	100	HQASLRLGQTSNHGWGFTLL
5	BPSS0530 [41-60]	SNHGWGFTLLDLDQDGLGLG	100	SNHGWGFTLLDLDQDGLGLG	100	SNHGWGFTLLDLDQDGLGLG
6	BPSS0530 [51-70]	DLDQDGLGLGRLGLRHARGV	100	DLDQDGLGLGRLGLRHARGV	100	DLDQDGLGLGRLGLRHARGV
7	BPSS0530 [61-80]	RLGLRHARGVFQDGTAFSLP	100	RLGLRHARGVFQDGTAFSLP	100	RLGLRHARGVFQDGTAFSLP
8	BPSS0530 [71-90]	FQDGTAFSLPSDDPLPPPLE	100	FQDGTAFSLPSDDPLPPPLE	100	FQDGTAFSLPSDDPLPPPLE
9	BPSS0530 [81-100]	SDDPLPPPLETELAQAGDIA	95	SDDPLPPPLETELAQAGD**V**A	100	SDDPLPPPLETELAQAGDIA
10	BPSS0530 [91-110]	TELAQAGDIACLALQAARTG	95	TELAQAGD**V**ACLALQAARTG	100	TELAQAGDIACLALQAARTG
11	BPSS0530 [101-120]	CLALQAARTGGPEMAFGDVE	95	CLALQAARTGGPEMAFGDV**A**	100	CLALQAARTGGPEMAFGDVE
12	BPSS0530 [111-130]	GPEMAFGDVELASRYRAVST	90	GPEMAFGDV**AS**ASRYRAVST	100	GPEMAFGDVELASRYRAVST
13	BPSS0530 [121-140]	LASRYRAVSTEVPDLAVGLD	95	**S**ASRYRAVSTEVPDLAVGLD	100	LASRYRAVSTEVPDLAVGLD
14	BPSS0530 [131-150]	EVPDLAVGLDAPGTPRRLTI	100	EVPDLAVGLDAPGTPRRLTI	100	EVPDLAVGLDAPGTPRRLTI
15	BPSS0530 [141-160]	APGTPRRLTIETGQLVTRLC	95	APGTPRRLTIETGQL**I**TRLC	100	APGTPRRLTIETGQLVTRLC
16	BPSS0530 [151-170]	ETGQLVTRLCWKSQLRSDEV	95	ETGQL**I**TRLCWKSQLRSDEV	100	ETGQLVTRLCWKSQLRSDEV
17	BPSS0530 [161-180]	WKSQLRSDEVALPIARVAGR	100	WKSQLRSDEVALPIARVAGR	100	WKSQLRSDEVALPIARVAGR
18	BPSS0530 [171-190]	ALPIARVAGRNASRTVSLDP	100	ALPIARVAGRNASRTVSLDP	100	ALPIARVAGRNASRTVSLDP
19	BPSS0530 [181-200]	NASRTVSLDPRFIPPLLDTR	100	NASRTVSLDPRFIPPLLDTR	100	NASRTVSLDPRFIPPLLDTR
20	BPSS0530 [191-210]	RFIPPLLDTRAHLVLRSLID	100	RFIPPLLDTRAHLVLRSLID	100	RFIPPLLDTRAHLVLRSLID
21	BPSS0530 [201-220]	AHLVLRSLIDELQSTLRVRL	100	AHLVLRSLIDELQSTLRVRL	100	AHLVLRSLIDELQSTLRVRL
22	BPSS0530 [211-230]	ELQSTLRVRLASTSAQRVLS	100	ELQSTLRVRLASTSAQRVLS	100	ELQSTLRVRLASTSAQRVLS
23	BPSS0530 [221-240]	ASTSAQRVLSTGGGVADLIE	100	ASTSAQRVLSTGGGVADLIE	100	ASTSAQRVLSTGGGVADLIE
24	BPSS0530 [231-250]	TGGGVADLIELLLRQAIAEY	100	TGGGVADLIELLLRQAIAEY	70	TGGGVADLIELLLR------
25	BPSS0530 [241-260]	LLLRQAIAEYRMRLANLDAF	100	LLLRQAIAEYRMRLANLDAF	0	
26	BPSS0530 [251-270]	RMRLANLDAFDPLPPAMLYH	100	RMRLANLDAFDPLPPAMLYH	0	
27	BPSS0530 [261-280]	DPLPPAMLYHELVGLLGRLS	100	DPLPPAMLYHELVGLLGRLS	0	
28	BPSS0530 [271-290]	ELVGLLGRLSVLPGVDEELA	100	ELVGLLGRLSVLPGVDEELA	0	
29	BPSS0530 [281-300]	VLPGVDEELADRELGYDHDD	100	VLPGVDEELADRELGYDHDD	0	
30	BPSS0530 [291-310]	DRELGYDHDDLQTSFEPLAM	100	DRELGYDHDDLQTSFEPLAM	0	
31	BPSS0530 [301-320]	LQTSFEPLAMMLRQALARVI	100	LQTSFEPLAMMLRQALARVI	0	
32	BPSS0530 [311-330]	MLRQALARVIETPVLPLRFE	100	MLRQALARVIETPVLPLRFE	0	
33	BPSS0530 [321-340]	ETPVLPLRFEDRGDQVHICI	100	ETPVLPLRFEDRGDQVHICI	0	
34	BPSS0530 [331-350]	DRGDQVHICIVDKQWNLKKL	100	DRGDQVHICIVDKQWNLKKL	0	
35	BPSS0530 [341-360]	VDKQWNLKKLIFAFSAAMPA	100	VDKQWNLKKLIFAFSAAMPA	0	
36	BPSS0530 [351-370]	IFAFSAAMPAEKLRQLLPQQ	100	IFAFSAAMPAEKLRQLLPQQ	0	
37	BPSS0530 [361-380]	EKLRQLLPQQTKLGAVEQIQ	100	EKLRQLLPQQTKLGAVEQIQ	0	
38	BPSS0530 [371-390]	TKLGAVEQIQKLVDLQLPGA	100	TKLGAVEQIQKLVDLQLPGA	0	
39	BPSS0530 [381-400]	KLVDLQLPGARLNALPNPPR	100	KLVDLQLPGARLNALPNPPR	0	
40	BPSS0530 [391-410]	RLNALPNPPRQIPYYAQSTY	100	RLNALPNPPRQIPYYAQSTY	0	
41	BPSS0530 [401-420]	QIPYYAQSTYFEVESTDPFW	100	QIPYYAQSTYFEVESTDPFW	0	
42	BPSS0530 [411-430]	FEVESTDPFWKQTLAGSAMA	100	FEVESTDPFWKQTLAGSAMA	0	
43	BPSS0530 [421-440]	KQTLAGSAMALRIVGDFPDL	100	KQTLAGSAMALRIVGDFPDL	0	
44	BPSS0530 [431-453]	LRIVGDFPDLRFEAWGLRDGKVA	100	LRIVGDFPDLRFEAWGLRDGKVA	0	

BPSS1385

Peptide No.	Antigen BPSS1385 Peptide	*Burkholderia pseudomallei*	*Burkholderia thailandensis* % homology	*Burkholderia thailandensis* Sequence	*Burkholderia mallei* % homology	*Burkholderia mallei* Sequence
1	BPSS1385 [1-20]	MLEHGVMKIPGINNVGKTGQ	0		0	
2	BPSS1385 [11-30]	GINNVGKTGQAGGETERIPS	0		0	
3	BPSS1385 [21-40]	AGGETERIPSTEPLGSSAAT	0		0	
4	BPSS1385 [31-50]	TEPLGSSAATSPAGPLGGLP	0		0	
5	BPSS1385 [41-60]	SPAGPLGGLPARSSSISNTN	0		0	
6	BPSS1385 [51-70]	ARSSSISNTNRTGENPMITP	0		0	
7	BPSS1385 [61-80]	RTGENPMITPIISSNLGLKH	0		0	
8	BPSS1385 [71-90]	IISSNLGLKHRVTLRKATLA	0		0	
9	BPSS1385 [81-100]	RVTLRKATLASLMQSLSGES	0		0	
10	BPSS1385 [91-110]	SLMQSLSGESSNRVMWNDRY	0		0	
11	BPSS1385 [101-120]	SNRVMWNDRYDTLLIARDPR	0		0	
12	BPSS1385 [111-130]	DTLLIARDPREIKNAIEKSV	0		0	
13	BPSS1385 [121-140]	EIKNAIEKSVTDFGGLENYK	0		0	
14	BPSS1385 [131-150]	TDFGGLENYKELTGGADPFA	0		0	
15	BPSS1385 [141-160]	ELTGGADPFALMTPVCGLSA	0		0	
16	BPSS1385 [151-170]	LMTPVCGLSANNIFKLMTEK	0		0	
17	BPSS1385 [161-180]	NNIFKLMTEKDVPIDPTSIE	0		0	
18	BPSS1385 [171-190]	DVPIDPTSIEYLENTSFAEH	0		0	
19	BPSS1385 [181-200]	YLENTSFAEHVNTLDSHKNY	0		0	
20	BPSS1385 [191-210]	VNTLDSHKNYVVIVNDGRLG	0		0	
21	BPSS1385 [201-220]	VVIVNDGRLGHKFLIDLPAL	0		0	
22	BPSS1385 [211-230]	HKFLIDLPALTQGPRTAYII	0		0	
23	BPSS1385 [221-240]	TQGPRTAYIIQSDLGGGALP	0		0	
24	BPSS1385 [231-250]	QSDLGGGALPAVRVEDWISR	0		0	
25	BPSS1385 [241-260]	AVRVEDWISRRGSDPVSLDE	0		0	
26	BPSS1385 [251-270]	RGSDPVSLDELNQLLSKDFS	0		0	
27	BPSS1385 [261-280]	LNQLLSKDFSKMPDDVQTRL	0		0	
28	BPSS1385 [271-290]	KMPDDVQTRLLASILQIDKD	0		0	
29	BPSS1385 [281-300]	LASILQIDKDPHKVDIKKLH	0		0	
30	BPSS1385 [291-310]	PHKVDIKKLHLDGKLRFASH	0		0	
31	BPSS1385 [301-320]	LDGKLRFASHEYDFRQFQRN	0		0	
32	BPSS1385 [311-328]	EYDFRQFQRNAQYVAGLG	0		0	

BPSS1492

	Antigen BPSS1492	*Burkholderia pseudomallei*	*Burkholderia thailandensis*		*Burkholderia mallei*	
Peptide No.	Peptide		% homology	Sequence	% homology	Sequence
1	BPSS1492 [1-20]	MHAKASSSHAPDAPKPSSIA	0		0	
2	BPSS1492 [11-30]	PDAPKPSSIATTLCRALASL	0		0	
3	BPSS1492 [21-40]	TTLCRALASLSLGLSMDAEA	0		0	
4	BPSS1492 [31-50]	SLGLSMDAEANPPEPPGGTN	0		0	
5	BPSS1492 [41-60]	NPPEPPGGTNIPVPPPMPGG	0		0	
6	BPSS1492 [51-70]	IPVPPPMPGGGANIPVPPPM	0		0	
7	BPSS1492 [61-80]	GANIPVPPPMPGGGANIPPP	0		0	
8	BPSS1492 [71-90]	PGGGANIPPPPPPPGGIGGA	0		0	
9	BPSS1492 [81-100]	PPPPGGIGGATPSPPPLTPV	0		0	
10	BPSS1492 [91-110]	TPSPPPLTPVNGNPGASTPT	0		0	
11	BPSS1492 [101-120]	NGNPGASTPTKTGLLKTLNR	0		0	
12	BPSS1492 [111-130]	KTGLLKTLNRLSAELQNNPR	0		0	
13	BPSS1492 [121-140]	LSAELQNNPRVTEDVVDNVD	0		0	
14	BPSS1492 [131-150]	VTEDVVDNVDAVIRNAVNLA	0		0	
15	BPSS1492 [141-160]	AVIRNAVNLAPDANGDFSGR	0		0	
16	BPSS1492 [151-170]	PDANGDFSGRSAMPIEMAAN	0		0	
17	BPSS1492 [161-180]	SAMPIEMAANAALRSLKKNP	0		0	
18	BPSS1492 [171-190]	AALRSLKKNPGDAGHAAPAY	0		0	
19	BPSS1492 [181-200]	GDAGHAAPAYLPAERIGQLR	0		0	
20	BPSS1492 [191-210]	LPAERIGQLREKVRRTIEAL	0		0	
21	BPSS1492 [201-220]	EKVRRTIEALESNRPPKPQP	0		0	
22	BPSS1492 [211-230]	ESNRPPKPQPRSTPPQSTPP	0		0	
23	BPSS1492 [221-240]	RSTPPQSTPPKPTQHPTAPN	0		0	
24	BPSS1492 [231-250]	KPTQHPTAPNPNVPDASTPD	0		0	
25	BPSS1492 [241-260]	PNVPDASTPDASTPDASTPD	0		0	
26	BPSS1492 [251-270]	ASTPDASTPDASTPDASTPS	0		0	
27	BPSS1492 [261-280]	ASTPDASTPSRPAPAPRAGT	0		0	
28	BPSS1492 [271-290]	RPAPAPRAGTGAPAASAATR	0		0	
29	BPSS1492 [281-300]	GAPAASAATRAPAFANRVRK	0		0	
30	BPSS1492 [291-310]	APAFANRVRKPNPAMPAASS	0		0	
31	BPSS1492 [301-320]	PNPAMPAASSHAIASDFASS	0		25	PNATPTSGSATNVTINFNST
32	BPSS1492 [311-330]	HAIASDFASSNAFAIGDDST	30	KYVAVNSGASDAFAAGVNAV	30	TNVTINFNSTGASAMGTNSI
33	BPSS1492 [321-340]	NAFAIGDDSTAVGAQAIAFS	45	DAFAAGVNAVAIGADARAQG	35	GASAMGTNSIALDFHARAKD
34	BPSS1492 [331-350]	AVGAQAIAFSEQSIAIGSRA	50	AIGADARAQGQESLATGWRA	35	ALDFHARAKDSDSLASGRLA
35	BPSS1492 [341-360]	EQSIAIGSRAIAAGARSIAV	45	QESLATGWRAQADGHRAVAT	45	SDSLASGRLAHASGPRSTAI
36	BPSS1492 [351-370]	IAAGARSIAVGTDATAAAPD	40	QADGHRAVATGARAIASGRD	40	HASGPRSTAIGAEANASGQN
37	BPSS1492 [361-380]	GTDATAAAPDSVALGSGSIA	60	GARAIASGRDAVALGAGSIA	55	GAEANASGQNTVALGAGSIA
38	BPSS1492 [371-390]	SVALGSGSIAEREGTVSVGR	70	AVALGAGSIADRDNTVSVGQ	75	TVALGAGSIADRNNTVSVGR
39	BPSS1492 [381-400]	EREGTVSVGRDGHERQITHV	65	DRDNTVSVGQRGSERQIVHV	70	DRNNTVSVGRHGDERQIVHV
40	BPSS1492 [391-410]	DGHERQITHVASGTEPTDAV	65	RGSERQIVHVAPGAQGTDAV	70	HGDERQIVHVAAGTQATDAV
41	BPSS1492 [401-420]	ASGTEPTDAVNVTQLRAAMS	60	APGAQGTDAVNVDQLNLAIS	70	AAGTQATDAVNVGQLNLAMS
42	BPSS1492 [411-430]	NVTQLRAAMSNANAYTNQRI	75	NVDQLNLAISNSNAYTNQRI	85	NVGQLNLAMSNANAYTNQRI
43	BPSS14920 [421-440]	NANAYTNQRIGDLQQSITDT	90	NSNAYTNQRIGDLQQSITET	100	NANAYTNQRIGDLQQSITDT
44	BPSS1492 [431-450]	GDLQQSITDTARDAYSGVAA	95	GDLQQSITETARDAYSGVAA	100	GDLQQSITDTARDAYSGVAA
45	BPSS1492 [441-460]	ARDAYSGVAAATALTMIPDV	100	ARDAYSGVAAATALTMIPDV	100	ARDAYSGVAAATALTMIPDV
46	BPSS1492 [451-470]	ATALTMIPDVDRDKRVSIGV	90	ATALTMIPDVDRDKMLSIGV	100	ATALTMIPDVDRDKRVSIGV
47	BPSS1492 [461-480]	DRDKRVSIGVGGAVYKGHRA	90	DRDKMLSIGVGGAVYKGHRA	100	DRDKRVSIGVGGAVYKGHRA
48	BPSS1492 [471-490]	GGAVYKGHRAVALGGTARIN	95	GGAVYKGHRAVALGGTARIG	100	GGAVYKGHRAVALGGTARIN
49	BPSS1492 [481-500]	VALGGTARINENLKVRAGVA	95	VALGGTARIGENLKVRAGVA	100	VALGGTARINENLKVRAGVA
50	BPSS1492 [491-516]	ENLKVRAGVAMSAGGNAVGIGMSWQW	92	ENLKVRAGVAMSAGGNTVGVGMSWQW	100	ENLKVRAGVAMSAGGNAVGIGMSWQW

BPSS1525

	Antigen BPSS1525	*Burkholderia pseudomallei*	*Burkholderia thailandensis*		*Burkholderia mallei*	
Peptide No.	Peptide		% homology	Sequence	% homology	Sequence
1	BPSS1525 [1-20]	MTYNPRIGGFTHVKQASFDV	100	MTYNPRIGGFTHVKQASFDV	0	
2	BPSS1525 [11-30]	THVKQASFDVHVKRGEAQPR	90	THVKQASFDVHVKR**S**EA**R**PR	0	
3	BPSS1525 [21-40]	HVKRGEAQPRTSFAQQIKRI	90	HVKR**S**EA**R**PRTSFAQQIKRI	0	
4	BPSS1525 [31-50]	TSFAQQIKRIFSKIGETLGQ	100	TSFAQQIKRIFSKIGETLGQ	0	
5	BPSS1525 [41-60]	FSKIGETLGQLFRHRAPDSA	95	FSKIGETLGQLFRHRAPD**G**A	0	
6	BPSS1525 [51-70]	LFRHRAPDSAPGRVRLQGVR	90	LFRHRAPD**G**APGRV**K**LQGVR	0	
7	BPSS1525 [61-80]	PGRVRLQGVRYVGSYRPTGD	95	PGRV**K**LQGVRYVGSYRPTGD	0	
8	BPSS1525 [71-90]	YVGSYRPTGDAKQAIRHFVD	95	YVGSYRPTGDA**R**QAIRHFVD	0	
9	BPSS1525 [81-100]	AKQAIRHFVDEAVKQVAHAR	95	A**R**QAIRHFVDEAVKQVAHAR	0	
10	BPSS1525 [91-110]	EAVKQVAHARTPEIRQDAEF	100	EAVKQVAHARTPEIRQDAEF	0	
11	BPSS1525 [101-120]	TPEIRQDAEFGRQVYEATLC	100	TPEIRQDAEFGRQVYEATLC	0	
12	BPSS1525 [111-130]	GRQVYEATLCAIFSEAKDRF	100	GRQVYEATLCAIFSEAKDRF	0	
13	BPSS1525 [121-140]	AIFSEAKDRFCMDPATRAGN	100	AIFSEAKDRFCMDPATRAGN	0	
14	BPSS1525 [131-150]	CMDPATRAGNVRPAFIEALG	95	CMDPATRAGNVRPAFI**A**ALG	0	
15	BPSS1525 [141-160]	VRPAFIEALGDAARATGLPG	90	VRPAFI**A**ALGDAARATGLPG	0	
16	BPSS1525 [151-170]	DAARATGLPGADKQGVFTPS	100	DAARATGLPGADKQGVFTPS	0	
17	BPSS1525 [161-180]	ADKQGVFTPSGAGTNPLYTE	100	ADKQGVFTPSGAGTNPLYTE	0	
18	BPSS1525 [171-190]	GAGTNPLYTEIRLRADTLMG	100	GAGTNPLYTEIRLRADTLMG	0	
19	BPSS1525 [181-200]	IRLRADTLMGAELAARPEYR	100	IRLRADTLMGAELAARPEYR	0	
20	BPSS1525 [191-210]	AELAARPEYRELQPYARQQA	95	AELAARPEYRELQ**S**YARQQA	0	
21	BPSS1525 [201-220]	ELQPYARQQAIDLVANALPA	90	ELQ**S**YARQQAIDLVANALP**G**	0	
22	BPSS1525 [210-230]	IDLVANALPAERSNTLVEFR	90	IDLVANALP**G**ERSNTL**A**EFR	0	
23	BPSS1525 [221-240]	ERSNTLVEFRQTVQTLEATY	95	ERSNTL**A**EFRQTVQTLEATY	0	
24	BPSS1525 [231-250]	QTVQTLEATYRRAAQDASRD	100	QTVQTLEATYRRAAQDASRD	0	
25	BPSS1525 [241-261]	RRAAQDASRDEKGATNAADGA	95	RRAAQDASRDEKGA**A**NAADGA	0	

BPSS1531

	Antigen BPSS1531	*Burkholderia pseudomallei*	*Burkholderia thailandensis*		*Burkholderia mallei*	
Peptide No.	Peptide		% homology	Sequence	% homology	Sequence
1	BPSS1531 [1-20]	MSIGVQSSGINISHAELSRL	95	MSIGVQSSGINISNAELSRL	75	MQSSGINISHAELSRL
2	BPSS1531 [11-30]	NISHAELSRLVDAGKSEQGD	80	NISNAELSRLVDAGMSELGG	100	NISHAELSRLVDAGKSEQGD
3	BPSS1531 [21-40]	VDAGKSEQGDKAVRDDGRAL	85	VDAGMSELGGKAVRDDGRAL	100	VDAGKSEQGDKAVRDDGRAL
4	BPSS1531 [31-50]	KAVRDDGRALARADAALAAV	95	KAVRDDGRALARAEAALAAV	100	KAVRDDGRALARADAALAAV
5	BPSS1531 [41-60]	ARADAALAAVVGERVAARRD	85	ARAEAALAAVVGERVSPRRD	100	ARADAALAAVVGERVAARRD
6	BPSS1531 [51-70]	VGERVAARRDAVAGSGAQRV	75	VGERVSPRRDA--GAGAQRV	100	VGERVAARRDAVAGSGAQRV
7	BPSS1531 [61-80]	AVAGSGAQRVELARPKPDAQ	75	RDAGAGAQRVELAQPKPAAQ	100	AVAGSGAQRVELARPKPDAQ
8	BPSS1531 [71-90]	ELARPKPDAQTRATDRRTVS	90	ELAQPKPAAQTRATDRRTVS	100	ELARPKPDAQTRATDRRTVS
9	BPSS1531 [81-100]	TRATDRRTVSGLEREHKRLA	90	TRATDRRTVSGMEREHRRLA	100	TRATDRRTVSGLEREHKRLA
10	BPSS1531 [91-110]	GLEREHKRLAASQTPRVTGM	80	GMEREHRRLAASQMPAVTGM	100	GLEREHKRLAASQTPRVTGM
11	BPSS1531 [101-120]	ASQTPRVTGMHDALVQRHVS	85	ASQMPAVTGMHEALVQRHVS	100	ASQTPRVTGMHDALVQRHVS
12	BPSS1531 [111-130]	HDALVQRHVSLDGAKAAHGE	60	HEALVQRHVSPGGAKTPGEG	100	HDALVQRHVSLDGAKAAHGE
13	BPSS1531 [121-140]	LDGAKAAHGEGVKRAAGDAP	45	PGGAKTPGEGGATRVGGDAP	100	LDGAKAAHGEGVKRAAGDAP
14	BPSS1531 [131-150]	GVKRAAGDAPRAAADAPQRF	40	GATRVGGDAPRFSFAEDKAF	100	GVKRAAGDAPRAAADAPQRF
15	BPSS1531 [141-160]	RAAADAPQRFAFADDKAFDA	50	TRVGGDAPRFSFAEDKAFDA	100	RAAADAPQRFAFADDKAFDA
16	BPSS1531 [151-170]	AFADDKAFDAMLALGAAMQK	80	SFAEDKAFDAMIALGIAMQK	100	AFADDKAFDAMLALGAAMQK
17	BPSS1531 [161-180]	MLALGAAMQKNVQSDLAMQG	85	MIALGIAMQKNVQSDLVMQG	100	MLALGAAMQKNVQSDLAMQG
18	BPSS1531 [171-190]	NVQSDLAMQGKLTMLAHDAM	90	NVQSDLVMQGKLTTLAHDAM	100	NVQSDLAMQGKLTMLAHDAM
19	BPSS1531 [181-200]	KLTMLAHDAMMSAAAQDRSI	95	KLTTLAHDAMMSAAAQDRSI	100	KLTMLAHDAMMSAAAQDRSI
20	BPSS1531 [191-210]	MSAAAQDRSIGAAQMTAAIA	100	MSAAAQDRSIGAAQMTAAIA	100	MSAAAQDRSIGAAQMTAAIA
21	BPSS1531 [201-220]	GAAQMTAAIAGGALQATTSL	95	GAAQMTAAIAGGALQAATSL	100	GAAQMTAAIAGGALQATTSL
22	BPSS1531 [211-230]	GGALQATTSLGGAMQQMKSL	85	GGALQAATSLGGAVQQMKGL	100	GGALQATTSLGGAMQQMKSL
23	BPSS1531 [221-240]	GGAMQQMKSLSTKSMSIEKE	85	GGAVQQMKGLGTKSMSIEKE	100	GGAMQQMKSLSTKSMSIEKE
24	BPSS1531 [231-250]	STKSMSIEKELKPQAELKQF	95	GTKSMSIEKELKPQAELKQF	100	STKSMSIEKELKPQAELKQF
25	BPSS1531 [241-260]	LKPQAELKQFHAEQALELRG	100	LKPQAELKQFHAEQALELRG	100	LKPQAELKQFHAEQALELRG
26	BPSS1531 [251-270]	HAEQALELRGINKPVLSNDE	100	HAEQALELRGINKPVLSNDE	100	HAEQALELRGINKPVLSNDE
27	BPSS1531 [261-280]	INKPVLSNDEVSHVKIKRDT	90	INKPVLSNDEVSHVKVKRET	100	INKPVLSNDEVSHVKIKRDT
28	BPSS1531 [271-290]	VSHVKIKRDTGETVRHEIDH	80	VSHVKVKRETGESVRHEIDP	100	VSHVKIKRDTGETVRHEIDH
29	BPSS1531 [281-300]	GETVRHEIDHGGERMSDEHA	90	GESVRHEIDPGGERMSDEHA	100	GETVRHEIDHGGERMSDEHA
30	BPSS1531 [291-310]	GGERMSDEHASVLAQEAPAR	100	GGERMSDEHASVLAQEAPAR	100	GGERMSDEHASVLAQEAPAR
31	BPSS1531 [301-320]	SVLAQEAPARQHRIDMHGMR	100	SVLAQEAPARQHRIDMHGMR	100	SVLAQEAPARQHRIDMHGMR
32	BPSS1531 [311-330]	QHRIDMHGMRHEENLVKAGR	95	QHRIDMHGMRHEQNLVKAGR	100	QHRIDMHGMRHEENLVKAGR
33	BPSS1531 [321-340]	HEENLVKAGRQQMKGDLLQS	90	HEQNLVKAGRQQMKGDLIQS	100	HEENLVKAGRQQMKGDLLQS
34	BPSS1531 [331-350]	QQMKGDLLQSGGQIGKNQID	90	QQMKGDLIQSGGQVGKNQID	100	QQMKGDLLQSGGQIGKNQID
35	BPSS1531 [341-360]	GGQIGKNQIDGASAQQQGAD	90	GGQVGKNQIDGASAQQQGAE	100	GGQIGKNQIDGASAQQQGAD
36	BPSS1531 [351-370]	GASAQQQGADRAEQKEDENA	90	GASAQQQGAERAEQKEDESA	100	GASAQQQGADRAEQKEDENA
37	BPSS1531 [361-380]	RAEQKEDENAQQTAMAAAST	90	RAEQKEDESAQQTAMAAASA	100	RAEQKEDENAQQTAMAAAST
38	BPSS1531 [371-390]	QQTAMAAASTRDEAAHRSRE	90	QQTAMAAASARDEAAHRGRE	100	QQTAMAAASTRDEAAHRSRE
39	BPSS1531 [381-400]	RDEAAHRSREAAQKAIDAAK	95	RDEAAHRGREAAQKAIDAAK	100	RDEAAHRSREAAQKAIDAAK
40	BPSS1531 [391-410]	AAQKAIDAAKSQVANDNAVA	100	AAQKAIDAAKSQVANDNAVA	100	AAQKAIDAAKSQVANDNAVA
41	BPSS1531 [401-419]	SQVANDNAVAAQVAGNLRT	100	SQVANDNAVAAQVAGNLRT	100	SQVANDNAVAAQVAGNLRT

BPSS1532

Peptide No.	Antigen BPSS1532 Peptide	*Burkholderia pseudomallei*	*Burkholderia thailandensis* % homology	*Burkholderia thailandensis* Sequence	*Burkholderia mallei* % homology	*Burkholderia mallei* Sequence
1	BPSS1532 [1-20]	MSSGVQGGPAANANAYQTHP	95	MSSGVQGGSAANANAYQTHP	100	MSSGVQGGPAANANAYQTHP
2	BPSS1532 [11-30]	ANANAYQTHPLRDAASALGT	100	ANANAYQTHPLRDAASALGT	100	ANANAYQTHPLRDAASALGT
3	BPSS1532 [21-40]	LRDAASALGTLSPQAYVDVV	100	LRDAASALGTLSPQAYVDVV	100	LRDAASALGTLSPQAYVDVV
4	BPSS1532 [31-50]	LSPQAYVDVVSAAQRNFLER	100	LSPQAYVDVVSAAQRNFLER	100	LSPQAYVDVVSAAQRNFLER
5	BPSS1532 [41-60]	SAAQRNFLERMSQLASEQCD	95	SAAQRNFLERMSRLASEQCD	100	SAAQRNFLERMSQLASEQCD
6	BPSS1532 [51-70]	MSQLASEQCDAQPAAHDARL	80	MSRLASEQCDAQPVTDDARL	100	MSQLASEQCDAQPAAHDARL
7	BPSS1532 [61-80]	AQPAAHDARLDDRPALRAPQ	45	AQPVTDDARLDRLDDKPALR	100	AQPAAHDARLDDRPALRAPQ
8	BPSS1532 [71-90]	DDRPALRAPQERDAPPLGAS	35	DDKPALRAPRDSAAHAADGN	100	DDRPALRAPQERDAPPLGAS
9	BPSS1532 [81-100]	ERDAPPLGASDTGSRASGAA	25	DAAHAADGNARGNGGASGAA	100	ERDAPPLGASDTGSRASGAA
10	BPSS1532 [91-110]	DTGSRASGAAKLTELLGVLM	80	RGNGGASGAAKLTELLGVLM	100	DTGSRASGAAKLTELLGVLM
11	BPSS1532 [101-120]	KLTELLGVLMSVISASSLDE	100	KLTELLGVLMSVISASSLDE	100	KLTELLGVLMSVISASSLDE
12	BPSS1532 [111-130]	SVISASSLDELKQRSDIWNQ	95	SVISASSLDELRQRSDIWNQ	100	SVISASSLDELKQRSDIWNQ
13	BPSS1532 [121-140]	LKQRSDIWNQMSKAAQDNLS	90	LRQRSDIWNQMSKAAQDNLT	100	LKQRSDIWNQMSKAAQDNLS
14	BPSS1532 [131-150]	MSKAAQDNLSRLSDAFQRAT	80	MSKAAQDNLTSLSDKFQCAT	100	MSKAAQDNLSRLSDAFQRAT
15	BPSS1532 [141-160]	RLSDAFQRATDEAKAAADAA	75	SLSDKFQCATDDAKAATDAA	100	RLSDAFQRATDEAKAAADAA
16	BPSS1532 [151-170]	DEAKAAADAAEQAAAAAKQA	85	DDAKAATDAAERAAAAAKQA	100	DEAKAAADAAEQAAAAAKQA
17	BPSS1532 [161-180]	EQAAAAAKQAGADAKAADAA	90	ERAAAAAKQAAADAKAADAA	100	EQAAAAAKQAGADAKAADAA
18	BPSS1532 [171-190]	GADAKAADAAVDAAQKRYDD	90	AADAKAADAAADAAQKRYDD	100	GADAKAADAAVDAAQKRYDD
19	BPSS1532 [181-200]	VDAAQKRYDDAVKQGLPDDR	90	ADAAQKRYDDAVKQGLPDDQ	100	VDAAQKRYDDAVKQGLPDDR
20	BPSS1532 [191-210]	AVKQGLPDDRLQSLKAALEQ	80	AVKQGLPDDQLKTLQAALEQ	100	AVKQGLPDDRLQSLKAALEQ
21	BPSS1532 [201-220]	LQSLKAALEQARQQAGDAHG	75	LKTLQAALEQAKQQAGDAHA	100	LQSLKAALEQARQQAGDAHG
22	BPSS1532 [211-230]	ARQQAGDAHGRADALQADAT	85	AKQQAGDAHARADALQADAA	100	ARQQAGDAHGRADALQADAT
23	BPSS1532 [221-240]	RADALQADATKKLDAASALA	85	RADALQADAAGKLDAATALA	100	RADALQADATKKLDAASALA
24	BPSS1532 [231-250]	KKLDAASALATQARACEQQV	75	GKLDAATALATQAREWEQQI	100	KKLDAASALATQARACEQQV
25	BPSS1532 [241-260]	TQARACEQQVDDAVNQATQQ	75	TQAREWEQQIDDAVNQASRQ	100	TQARACEQQVDDAVNQATQQ
26	BPSS1532 [251-270]	DDAVNQATQQYGASASLRTP	90	DDAVNQASRQYGASASLRTP	100	DDAVNQATQQYGASASLRTP
27	BPSS1532 [261-280]	YGASASLRTPQSPRLSGAAE	90	YGASASLRTPPSPKLSGAAE	100	YGASASLRTPQSPRLSGAAE
28	BPSS1532 [271-290]	QSPRLSGAAELTAVLGKLQE	90	PSPKLSGAAELTAVLGKLQE	100	QSPRLSGAAELTAVLGKLQE
29	BPSS1532 [281-300]	LTAVLGKLQELISSGNVKEL	100	LTAVLGKLQELISSGNVKEL	100	LTAVLGKLQELISSGNVKEL
30	BPSS1532 [291-310]	LISSGNVKELESKQKLFTEM	100	LISSGNVKELESKQKLFTEM	100	LISSGNVKELESKQKLFTEM
31	BPSS1532 [301-320]	ESKQKLFTEMQAKREAELQK	100	ESKQKLFTEMQAKREAELQK	100	ESKQKLFTEMQAKREAELQK
32	BPSS1532 [311-330]	QAKREAELQKKSDEYQAQVK	95	QAKREAELQKKSDEYQEQVK	100	QAKREAELQKKSDEYQAQVK
33	BPSS1532 [321-340]	KSDEYQAQVKKAEEMQKTMG	95	KSDEYQEQVKKAEEMQKTMG	100	KSDEYQAQVKKAEEMQKTMG
34	BPSS1532 [331-350]	KAEEMQKTMGCIGKIVGWVI	100	KAEEMQKTMGCIGKIVGWVI	100	KAEEMQKTMGCIGKIVGWVI
35	BPSS1532 [341-360]	CIGKIVGWVITAVSFAAAAF	100	CIGKIVGWVITAVSFAAAAF	100	CIGKIVGWVITAVSFAAAAF
36	BPSS1532 [351-370]	TAVSFAAAAFTGGASLALAA	100	TAVSFAAAAFTGGASLALAA	100	TAVSFAAAAFTGGASLALAA
37	BPSS1532 [361-380]	TGGASLALAAVGLALAVGDE	100	TGGASLALAAVGLALAVGDE	100	TGGASLALAAVGLALAVGDE
38	BPSS1532 [371-390]	VGLALAVGDEISRATTGVSF	100	VGLALAVGDEISRATTGVSF	100	VGLALAVGDEISRATTGVSF
39	BPSS1532 [381-400]	ISRATTGVSFMDKLMQPVMD	100	ISRATTGVSFMDKLMQPVMD	100	ISRATTGVSFMDKLMQPVMD
40	BPSS1532 [391-410]	MDKLMQPVMDAILKPLMEMI	95	MDKLMQPVMDAILKPLMEVI	100	MDKLMQPVMDAILKPLMEMI
41	BPSS1532 [401-420]	AILKPLMEMISSLITKALVA	95	AILKPLMEVISSLITKALVA	100	AILKPLMEMISSLITKALVA
42	BPSS1532 [411-430]	SSLITKALVACGVDQQKAEL	100	SSLITKALVACGVDQQKAEL	100	SSLITKALVACGVDQQKAEL
43	BPSS1532 [421-440]	CGVDQQKAELAGAILGAVVT	100	CGVDQQKAELAGAILGAVVT	100	CGVDQQKAELAGAILGAVVT
44	BPSS1532 [431-450]	AGAILGAVVTGVALVAAAFV	100	AGAILGAVVTGVALVAAAFV	100	AGAILGAVVTGVALVAAAFV
45	BPSS1532 [441-460]	GVALVAAAFVGASAVKAVAS	100	GVALVAAAFVGASAVKAVAS	100	GVALVAAAFVGASAVKAVAS
46	BPSS1532 [451-470]	GASAVKAVASKVIDAMAGQL	95	GASAVKAVASKVIDAVAGQL	100	GASAVKAVASKVIDAMAGQL
47	BPSS1532 [461-480]	KVIDAMAGQLTKLMDSAIGK	95	KVIDAVAGQLTKLMDSAIGK	100	KVIDAMAGQLTKLMDSAIGK
48	BPSS1532 [471-490]	TKLMDSAIGKMLVQLIEKFS	100	TKLMDSAIGKMLVQLIEKFS	100	TKLMDSAIGKMLVQLIEKFS
49	BPSS1532 [481-500]	MLVQLIEKFSEKSGLQALGS	100	MLVQLIEKFSEKSGLQALGS	100	MLVQLIEKFSEKSGLQALGS
50	BPSS1532 [491-510]	EKSGLQALGSRTATAMTRMR	100	EKSGLQALGSRTATAMTRMR	100	EKSGLQALGSRTATAMTRMR
51	BPSS1532 [501-520]	RTATAMTRMRRAIGVEAKED	100	RTATAMTRMRRAIGVEAKED	100	RTATAMTRMRRAIGVEAKED
52	BPSS1532 [511-530]	RAIGVEAKEDGMLLANRFEK	100	RAIGVEAKEDGMLLANRFEK	100	RAIGVEAKEDGMLLANRFEK
53	BPSS1532 [521-540]	GMLLANRFEKAGTVMNVGNQ	100	GMLLANRFEKAGTVMNVGNQ	100	GMLLANRFEKAGTVMNVGNQ
54	BPSS1532 [531-550]	AGTVMNVGNQVSQAAGGIVV	100	AGTVMNVGNQVSQAAGGIVV	100	AGTVMNVGNQVSQAAGGIVV
55	BPSS1532 [541-560]	VSQAAGGIVVGVERAKAMGL	100	VSQAAGGIVVGVERAKAMGL	100	VSQAAGGIVVGVERAKAMGL
56	BPSS1532 [551-570]	GVERAKAMGLLADVKEAMYD	100	GVERAKAMGLLADVKEAMYD	100	GVERAKAMGLLADVKEAMYD
57	BPSS1532 [561-580]	LADVKEAMYDIKLLGDLLKQ	100	LADVKEAMYDIKLLGDLLKQ	95	LADVKEAMYDIKLLGNLLKQ
58	BPSS1532 [571-590]	IKLLGDLLKQAVDAFAEHNR	95	IKLLGDLLKQAVDSFAEHNR	95	IKLLGNLLKQAVDAFAEHNR
59	BPSS1532 [581-600]	AVDAFAEHNRVLAQLMQQMS	95	AVDSFAEHNRVLAQLMQQMS	100	AVDAFAEHNRVLAQLMQQMS
60	BPSS1532 [591-610]	VLAQLMQQMSDAGEMQTSTG	100	VLAQLMQQMSDAGEMQTSTG	100	VLAQLMQQMSDAGEMQTSTG
61	BPSS1532 [601-620]	DAGEMQTSTGKLILRNARAV	100	DAGEMQTSTGKLILRNARAV	100	DAGEMQTSTGKLILRNARAV

BPSS1599

	Antigen BPSS1599	*Burkholderia pseudomallei*	*Burkholderia thailandensis*		*Burkholderia mallei*	
Peptide No.	Peptide		% homology	Sequence	% homology	Sequence
1	BPSS1599 [1-20]	MSAQVIQIGRQRFVGGLFWQ	100	MSAQVIQIGRQRFVGGLFWQ	100	MSAQVIQIGRQRFVGGLFWQ
2	BPSS1599 [11-30]	QRFVGGLFWQSLSRRNELRA	100	QRFVGGLFWQSLSRRNELRA	100	QRFVGGLFWQSLSRRNELRA
3	BPSS1599 [21-40]	SLSRRNELRAEAVELAKKLK	100	SLSRRNELRAEAVELAKKLK	100	SLSRRNELRAEAVELAKKLK
4	BPSS1599 [31-50]	EAVELAKKLKFDLMVLRIDR	100	EAVELAKKLKFDLMVLRIDR	100	EAVELAKKLKFDLMVLRIDR
5	BPSS1599 [41-60]	FDLMVLRIDRGVAAAGYANT	100	FDLMVLRIDRGVAAAGYANT	100	FDLMVLRIDRGVAAAGYANT
6	BPSS1599 [51-70]	GVAAAGYANTRDGFAPGHLS	100	GVAAAGYANTRDGFAPGHLS	100	GVAAAGYANTRDGFAPGHLS
7	BPSS1599 [61-80]	RDGFAPGHLSLGAMVSRAIA	100	RDGFAPGHLSLGAMVSRAIA	100	RDGFAPGHLSLGAMVSRAIA
8	BPSS1599 [71-90]	LGAMVSRAIALEGAFYNGRR	100	LGAMVSRAIALEGAFYNGRR	100	LGAMVSRAIALEGAFYNGRR
9	BPSS1599 [81-100]	LEGAFYNGRRQPAPNWLGAF	100	LEGAFYNGRRQPAPNWLGAF	100	LEGAFYNGRRQPAPNWLGAF
10	BPSS1599 [91-110]	QPAPNWLGAFALPDGRWAYF	100	QPAPNWLGAFALPDGRWAYF	100	QPAPNWLGAFALPDGRWAYF
11	BPSS1599 [101-120]	ALPDGRWAYFAVRDHAFMPN	100	ALPDGRWAYFAVRDHAFMPN	100	ALPDGRWAYFAVRDHAFMPN
12	BPSS1599 [111-130]	AVRDHAFMPNGDWVGSREEA	100	AVRDHAFMPNGDWVGSREEA	100	AVRDHAFMPNGDWVGSREEA
13	BPSS1599 [121-140]	GDWVGSREEALERLHTDYAW	100	GDWVGSREEALERLHTDYAW	100	GDWVGSREEALERLHTDYAW
14	BPSS1599 [131-150]	LERLHTDYAWGGWNVVIGEP	100	LERLHTDYAWGGWNVVIGEP	100	LERLHTDYAWGGWNVVIGEP
15	BPSS1599 [141-160]	GGWNVVIGEPELERQGFQNF	95	GGWNVVIGEPELEKQGFQNF	100	GGWNVVIGEPELERQGFQNF
16	BPSS1599 [151-170]	ELERQGFQNFQPKRLDDLLP	90	ELEKQGFQNFQPKRLDELLP	100	ELERQGFQNFQPKRLDDLLP
17	BPSS1599 [161-180]	QPKRLDDLLPRRGGRPRTER	95	QPKRLDELLPRRGGRPRTER	100	QPKRLDDLLPRRGGRPRTER
18	BPSS1599 [171-190]	RRGGRPRTERWWALRPVERR	95	RRGGRPRTERWWALKPVERR	100	RRGGRPRTERWWALRPVERR
19	BPSS1599 [181-200]	WWALRPVERRLSPRAALIAA	95	WWALKPVERRLSPRAALIAA	100	WWALRPVERRLSPRAALIAA
20	BPSS1599 [191-210]	LSPRAALIAATAACVVLGGA	85	LSPRAALIAATVACVAFGGA	100	LSPRAALIAATAACVVLGGA
21	BPSS1599 [201-220]	TAACVVLGGAFAYWHHRAKV	75	TVACVAFGGALAYWHHRAKL	100	TAACVVLGGAFAYWHHRAKV
22	BPSS1599 [211-230]	FAYWHHRAKVEAEEREAALE	85	LAYWHHRAKLEAQEREAALE	100	FAYWHHRAKVEAEEREAALE
23	BPSS1599 [221-240]	EAEEREAALERVRAELAARQ	95	EAQEREAALERVRAELAARQ	100	EAEEREAALERVRAELAARQ
24	BPSS1599 [231-250]	RVRAELAARQARSGPVAPPW	95	RVRAELAARQARGGPVAPPW	100	RVRAELAARQARSGPVAPPW
25	BPSS1599 [241-260]	ARSGPVAPPWAALPDALAFA	85	ARGGPVAPPWAKLPDAVAFA	100	ARSGPVAPPWAALPDALAFA
26	BPSS1599 [251-270]	AALPDALAFARACAMRFGRL	85	AKLPDAVAFARACARRFGRL	100	AALPDALAFARACAMRFGRL
27	BPSS1599 [261-280]	RACAMRFGRLAPGGWRLERY	90	RACARRFGRLSPGGWRLERY	100	RACAMRFGRLAPGGWRLERY
28	BPSS1599 [271-290]	APGGWRLERYECTPGTAHYA	95	SPGGWRLERYECTPGTAHYA	100	APGGWRLERYECTPGTAHYA
29	BPSS1599 [281-300]	ECTPGTAHYAWARNGSNVRY	100	ECTPGTAHYAWARNGSNVRY	100	ECTPGTAHYAWARNGSNVRY
30	BPSS1599 [291-310]	WARNGSNVRYLLVVEPGATL	80	WARNGSNVRYLLAMEPAATV	100	WARNGSNVRYLLVVEPGATL
31	BPSS1599 [301-320]	LLVVEPGATLDTDGERATLD	80	LLAMEPAATVDTDGERATLD	100	LLVVEPGATLDTDGERATLD
32	BPSS1599 [311-330]	DTDGERATLDVPLTAPTAND	95	DTDGERATLDVPLTAPTADD	100	DTDGERATLDVPLTAPTAND
33	BPSS1599 [321-340]	VPLTAPTANDTPLADDSVVR	90	VPLTAPTADDTPLVDDSVVR	100	VPLTAPTANDTPLADDSVVR
34	BPSS1599 [331-350]	TPLADDSVVRTQLLARLQWL	90	TPLVDDSVVRTQLLSRLQWL	100	TPLADDSVVRTQLLARLQWL
35	BPSS1599 [341-360]	TQLLARLQWLDAAAKLERLL	90	TQLLSRLQWLDAAAKLDRLL	100	TQLLARLQWLDAAAKLERLL
36	BPSS1599 [351-370]	DAAAKLERLLPEQGPRAPLA	80	DAAAKLDRLLPDQASRAPLA	100	DAAAKLERLLPEQGPRAPLA
37	BPSS1599 [361-380]	PEQGPRAPLANLAQQAAALP	75	PDQASRAPLADLAQQAAAAP	100	PEQGPRAPLANLAQQAAALP
38	BPSS1599 [371-390]	NLAQQAAALPASPTWRAYRL	70	DLAQQAAAAPAW---RAYRL	100	NLAQQAAALPASPTWRAYRL
39	BPSS1599 [381-400]	ASPTWRAYRLNANLGGIAPP	80	AAPAWRAYRLNASLGGVAPP	100	ASPTWRAYRLNANLGGIAPP
40	BPSS1599 [391-410]	NANLGGIAPPEFVRAIDVPG	90	NASLGGVAPPEFVRAIDVPG	100	NANLGGIAPPEFVRAIDVPG
41	BPSS1599 [401-420]	EFVRAIDVPGLRVERIAYQN	100	EFVRAIDVPGLRVERIAYQN	100	EFVRAIDVPGLRVERIAYQN
42	BPSS1599 [411-430]	LRVERIAYQNNQWTLEGVLY	100	LRVERIAYQNNQWTLEGVLY	100	LRVERIAYQNNQWTLEGVLY
43	BPSS1599 [421-432]	NQWTLEGVLYAK	100	NQWTLEGVLYAK	100	NQWTLEGVLYAK
44	BPSS1599 [413-432]	VERIAYQNNQWTLEGVLYAK	100	VERIAYQNNQWTLEGVLYAK	100	VERIAYQNNQWTLEGVLYAK

BPSS2141

Peptide No.	Antigen BPSS2141 Peptide	*Burkholderia pseudomallei*	*Burkholderia thailandensis* % homology	*Burkholderia thailandensis* Sequence	*Burkholderia mallei* % homology	*Burkholderia mallei* Sequence
1	BPSS2141 [1-20]	MRRALPFRYHYQSHTMKHTH	100	MRRALPFRYHYQSHTMKHTH	0	
2	BPSS2141 [11-30]	YQSHTMKHTHAFAAVLAALA	95	YQSHTMKHTHAFAAVLA**S**LA	75	----MKHTHAFAAVLAALA
3	BPSS2141 [21-40]	AFAAVLAALALTIAPSAPAV	85	AFAAVLA**S**LALTIA**Q**SA**S**AV	100	AFAAVLAALALTIAPSAPAV
4	BPSS2141 [31-50]	LTIAPSAPAVTVASNVTLAD	85	LTIA**Q**SA**S**AVTV**P**SNVTLAD	100	LTIAPSAPAVTVASNVTLAD
5	BPSS2141 [41-60]	TVASNVTLADQQDLTRQVPA	95	TV**P**SNVTLADQQDLTRQVPA	100	TVASNVTLADQQDLTRQVPA
6	BPSS2141 [51-70]	QQDLTRQVPAEVESLDPAHI	100	QQDLTRQVPAEVESLDPAHI	100	QQDLTRQVPAEVESLDPAHI
7	BPSS2141 [61-80]	EVESLDPAHIESWTGNTIGL	100	EVESLDPAHIESWTGNTIGL	100	EVESLDPAHIESWTGNTIGL
8	BPSS2141 [71-90]	ESWTGNTIGLDLFEGLARID	100	ESWTGNTIGLDLFEGLARID	100	ESWTGNTIGLDLFEGLARID
9	BPSS2141 [81-100]	DLFEGLARIDASGAVVPGVA	100	DLFEGLARIDASGAVVPGVA	100	DLFEGLARIDASGAVVPGVA
10	BPSS2141 [91-110]	ASGAVVPGVAQAWEHKAPDT	90	ASGAVVPGVAQ**S**WEHK**T**PDT	100	ASGAVVPGVAQAWEHKAPDT
11	BPSS2141 [101-120]	QAWEHKAPDTWIFKLRRDAK	90	Q**S**WEHK**T**PDTWIFKLRRDAK	100	QAWEHKAPDTWIFKLRRDAK
12	BPSS2141 [111-130]	WIFKLRRDAKWSNGQPVTAA	100	WIFKLRRDAKWSNGQPVTAA	100	WIFKLRRDAKWSNGQPVTAA
13	BPSS2141 [121-140]	WSNGQPVTAADFVYAWQRLA	95	WSNGQPVTAADFVY**S**WQRLA	100	WSNGQPVTAADFVYAWQRLA
14	BPSS2141 [131-150]	DFVYAWQRLADPKTGSKYTI	95	DFVY**S**WQRLADPKTGSKYTI	100	DFVYAWQRLADPKTGSKYTI
15	BPSS2141 [141-160]	DPKTGSKYTILVEFVKNASA	100	DPKTGSKYTILVEFVKNASA	100	DPKTGSKYTILVEFVKNASA
16	BPSS2141 [151-170]	LVEFVKNASAIIAGKQPPGD	95	LVEFVKNASAIIAGKQPPG**S**	100	LVEFVKNASAIIAGKQPPGD
17	BPSS2141 [161-180]	IIAGKQPPGDLGIRAIDPYT	95	IIAGKQPPG**S**LGIRAIDPYT	100	IIAGKQPPGDLGIRAIDPYT
18	BPSS2141 [171-190]	LGIRAIDPYTIEVKTEVPVS	95	LGIRAIDPYTIEV**T**TEVPVS	100	LGIRAIDPYTIEVKTEVPVS
19	BPSS2141 [181-200]	IEVKTEVPVSYFPELTAMAP	95	IEV**T**TEVPVSYFPELTAMAP	100	IEVKTEVPVSYFPELTAMAP
20	BPSS2141 [191-210]	YFPELTAMAPLTPVNKDAVA	90	YFPELTAMAPL**A**PVNKD**T**VA	100	YFPELTAMAPLTPVNKDAVA
21	BPSS2141 [201-220]	LTPVNKDAVAKFGDAWTRPK	90	L**A**PVNKD**T**VAKFGDAWTRPK	100	LTPVNKDAVAKFGDAWTRPK
22	BPSS2141 [211-230]	KFGDAWTRPKNIVSNGPYTL	90	KFGDAWTRPKNIV**G**NGPY**S**L	100	KFGDAWTRPKNIVSNGPYTL
23	BPSS2141 [221-240]	NIVSNGPYTLVDWQPNNRIV	90	NIV**G**NGPY**S**LVDWQPNNRIV	100	NIVSNGPYTLVDWQPNNRIV
24	BPSS2141 [231-250]	VDWQPNNRIVMAKSDKYWNA	100	VDWQPNNRIVMAKSDKYWNA	100	VDWQPNNRIVMAKSDKYWNA
25	BPSS2141 [241-260]	MAKSDKYWNARNVVIRKVTY	100	MAKSDKYWNARNVVIRKVTY	100	MAKSDKYWNARNVVIRKVTY
26	BPSS2141 [251-270]	RNVVIRKVTYLPIENDETAL	100	RNVVIRKVTYLPIENDETAL	100	RNVVIRKVTYLPIENDETAL
27	BPSS2141 [261-280]	LPIENDETALRMYQAGQIDY	100	LPIENDETALRMYQAGQIDY	100	LPIENDETALRMYQAGQIDY
28	BPSS2141 [271-290]	RMYQAGQIDYTYSIPAGGFG	100	RMYQAGQIDYTYSIPAGGFG	100	RMYQAGQIDYTYSIPAGGFG
29	BPSS2141 [281-300]	TYSIPAGGFGQISKQFGKEL	100	TYSIPAGGFGQISKQFGKEL	100	TYSIPAGGFGQISKQFGKEL
30	BPSS2141 [291-310]	QISKQFGKELRPGLQLATYY	100	QISKQFGKELRPGLQLATYY	100	QISKQFGKELRPGLQLATYY
31	BPSS2141 [301-320]	RPGLQLATYYYYLKNSDPAL	100	RPGLQLATYYYYLKNSDPAL	100	RPGLQLATYYYYLKNSDPAL
32	BPSS2141 [311-330]	YYLKNSDPALKDKRVREALA	100	YYLKNSDPALKDKRVREALA	100	YYLKNSDPALKDKRVREALA
33	BPSS2141 [321-340]	KDKRVREALAMVLDREILTS	95	KDKRVREALAMVLDR**D**ILTS	100	KDKRVREALAMVLDREILTS
34	BPSS2141 [331-350]	MVLDREILTSKITQAGEVPM	95	MVLDR**D**ILTSKITQAGEVPM	100	MVLDREILTSKITQAGEVPM
35	BPSS2141 [341-360]	KITQAGEVPMYGLMPKGVKG	100	KITQAGEVPMYGLMPKGVKG	100	KITQAGEVPMYGLMPKGVKG
36	BPSS2141 [351-370]	YGLMPKGVKGVQRPFTPDWA	100	YGLMPKGVKGVQRPFTPDWA	100	YGLMPKGVKGVQRPFTPDWA
37	BPSS2141 [361-380]	VQRPFTPDWASWPMARRVDY	95	VQRPFTPDWASWPMA**K**RVDY	100	VQRPFTPDWASWPMARRVDY
38	BPSS2141 [371-390]	SWPMARRVDYAKNLLKQAGH	90	SWPMA**K**RVDYAKNLLKQAG**Y**	100	SWPMARRVDYAKNLLKQAGH
39	BPSS2141 [381-400]	AKNLLKQAGHGDANPLTFTL	85	AKNLLKQAG**YS**DA**H**PLTFTL	100	AKNLLKQAGHGDANPLTFTL
40	BPSS2141 [391-410]	GDANPLTFTLTYNTNDLHKK	90	**S**DA**H**PLTFTLTYNTNDLHKK	100	GDANPLTFTLTYNTNDLHKK
41	BPSS2141 [401-420]	TYNTNDLHKKVALFAASEWR	100	TYNTNDLHKKVALFAASEWR	100	TYNTNDLHKKVALFAASEWR
42	BPSS2141 [411-430]	VALFAASEWRTKLGVTAKLE	100	VALFAASEWRTKLGVTAKLE	100	VALFAASEWRTKLGVTAKLE
43	BPSS2141 [421-440]	TKLGVTAKLENVEFKVLMKQ	100	TKLGVTAKLENVEFKVLMKQ	100	TKLGVTAKLENVEFKVLMKQ
44	BPSS2141 [431-450]	NVEFKVLMKQRHDGKVQIAR	95	NVEFKVLMKQRHDGKVQ**V**AR	95	NVEFKVLMKQRHDGKVQ**V**AR
45	BPSS2141 [441-460]	RHDGKVQIARDGWFADYNDA	95	RHDGKVQ**V**ARDGWFADYNDA	95	RHDGKVQ**V**ARDGWFADYNDA
46	BPSS2141 [451-470]	DGWFADYNDAMTFFDLIRCG	100	DGWFADYNDAMTFFDLIRCG	100	DGWFADYNDAMTFFDLIRCG
47	BPSS2141 [461-480]	MTFFDLIRCGSSQNTVGYCN	100	MTFFDLIRCGSSQNTVGYCN	100	MTFFDLIRCGSSQNTVGYCN
48	BPSS2141 [471-490]	SSQNTVGYCNPKVDSLVAEA	95	SSQNTVGYCNPKVD**A**LVAEA	100	SSQNTVGYCNPKVDSLVAEA
49	BPSS2141 [481-500]	PKVDSLVAEANQKLDDGARA	90	PKVD**A**LVAEANQKLDDGAR**S**	100	PKVDSLVAEANQKLDDGARA
50	BPSS2141 [491-510]	NQKLDDGARAALLTQAHDLA	95	NQKLDDGAR**S**ALLTQAHDLA	100	NQKLDDGARAALLTQAHDLA
51	BPSS2141 [501-520]	ALLTQAHDLAMNDYPMVPLF	95	ALLTQAHDLAMNDYPMV**S**LF	100	ALLTQAHDLAMNDYPMVPLF
52	BPSS2141 [511-530]	MNDYPMVPLFQYSADRLVKS	95	MNDYPMV**S**LFQYSADRLVKS	100	MNDYPMVPLFQYSADRLVKS
53	BPSS2141 [521-540]	QYSADRLVKSYVGGYTLTNY	100	QYSADRLVKSYVGGYTLTNY	100	QYSADRLVKSYVGGYTLTNY
54	BPSS2141 [531-554]	YVGGYTLTNYIDMRASQDMYLIKH	95	YVGGYTLTNYIDMRASQDMYL**A**KH	100	YVGGYTLTNYIDMRASQDMYLIKH
