# Supplementary material for: Immune Control of Burkholderia pseudomallei––Common, High-Frequency T-Cell Responses to a Broad Repertoire of Immunoprevalent Epitopes
Source: Front Immunol. 2018 Mar 20;9:484. doi: 10.3389/fimmu.2018.00484 (PMC5869189; doi:10.3389/fimmu.2018.00484)
Supplement: Supplementary file 4 [file image_1.PDF]

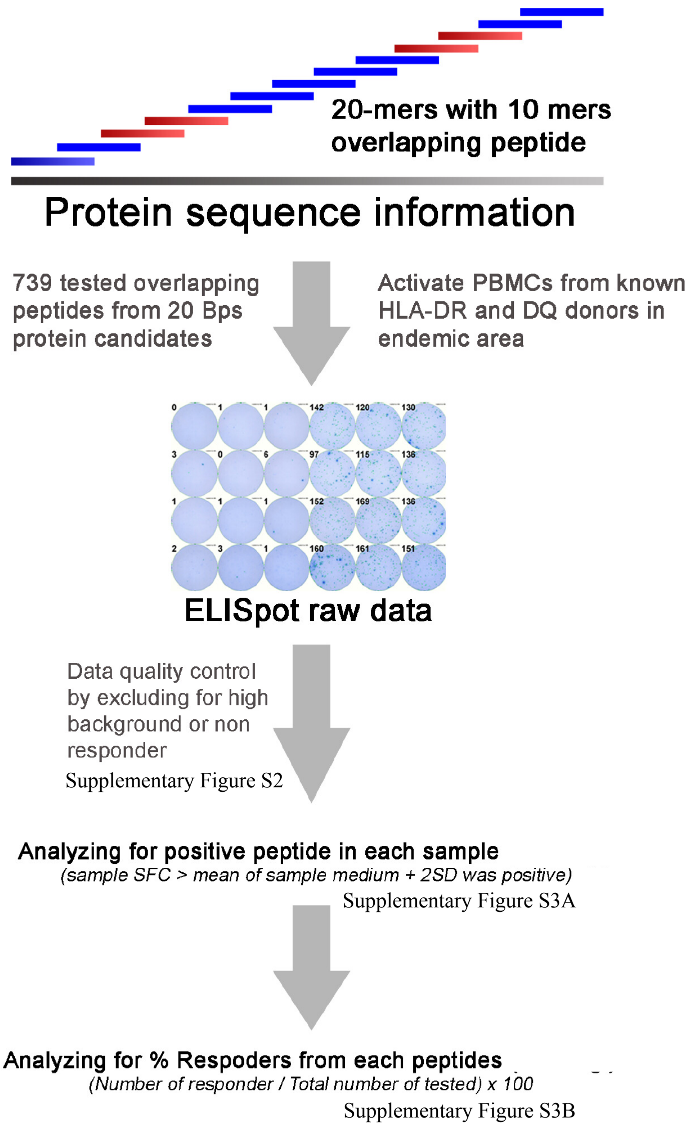

Figure S1: Strategy of data analysis pipeline for 20 Bp peptides libraries. Peptide libraries from 20 Bp antigen candidates were synthesized as 20-mers with a 10-mer overlap (739 peptides in total). Peptides were cultured with human PBMC for 48h and IFNγ SFC number was determined by ELISpot. Samples with a high background for medium only control and/or low response to intact Bp were excluded from further analysis. A positive responses to peptide was defined as the sample SFC being > mean of sample medium only controls + 2 S.D. The % Responder for each peptide was defined as the number of individuals that responded to a given peptide divided by the total number of donors tested multiplied by 100.
