# Supplementary material for: Immune Control of Burkholderia pseudomallei––Common, High-Frequency T-Cell Responses to a Broad Repertoire of Immunoprevalent Epitopes
Source: Front Immunol. 2018 Mar 20;9:484. doi: 10.3389/fimmu.2018.00484 (PMC5869189; doi:10.3389/fimmu.2018.00484)
Supplement: Supplementary file 5 [file image_2.PDF]

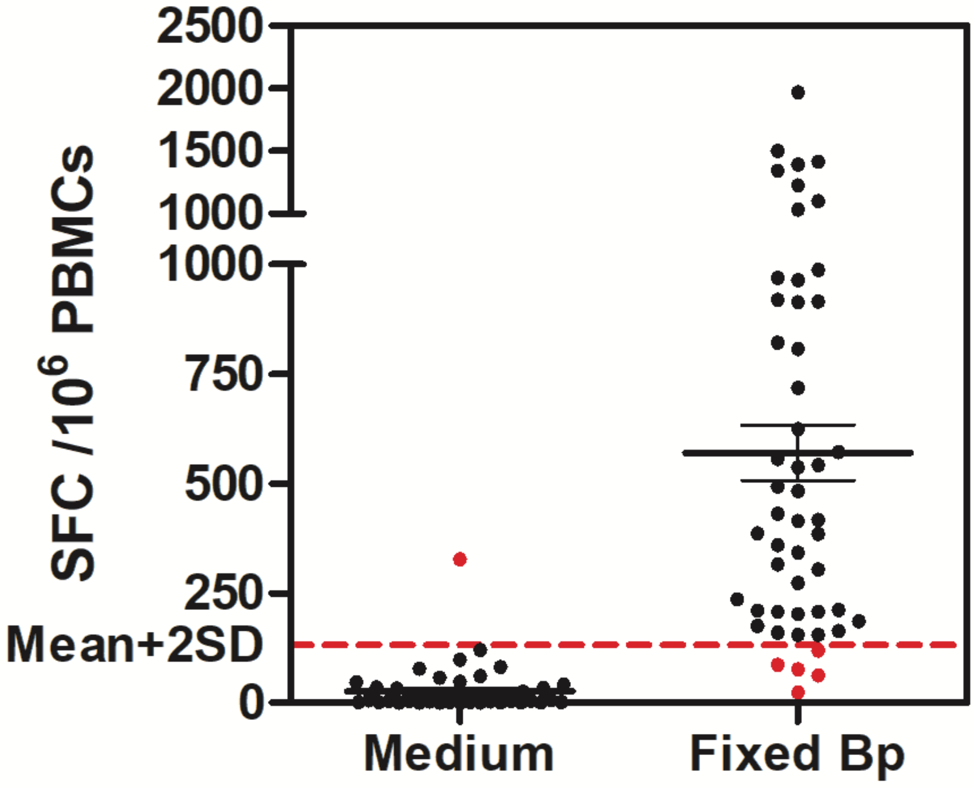

Figure S2: T cell response to medium only and Bp controls of 51 seropositive healthy donors. The y-axis represents IFNγ spot forming cells per one million PBMCs. The cut off (red line) was calculated by mean of medium only control + 2 S.D. of all donors. Any donor with a response greater than the mean + 2 S.D. of all medium only controls from all donors tested was considered a 'high background', and any donor with a response to Bp less than mean of medium only controls + 2 S.D. of all donors tested was considered a 'low responder' (red dot). These donors were excluded from further analysis.
