# Supplementary material for: Immune Control of Burkholderia pseudomallei––Common, High-Frequency T-Cell Responses to a Broad Repertoire of Immunoprevalent Epitopes
Source: Front Immunol. 2018 Mar 20;9:484. doi: 10.3389/fimmu.2018.00484 (PMC5869189; doi:10.3389/fimmu.2018.00484)
Supplement: Supplementary file 6 [file image_3.PDF]

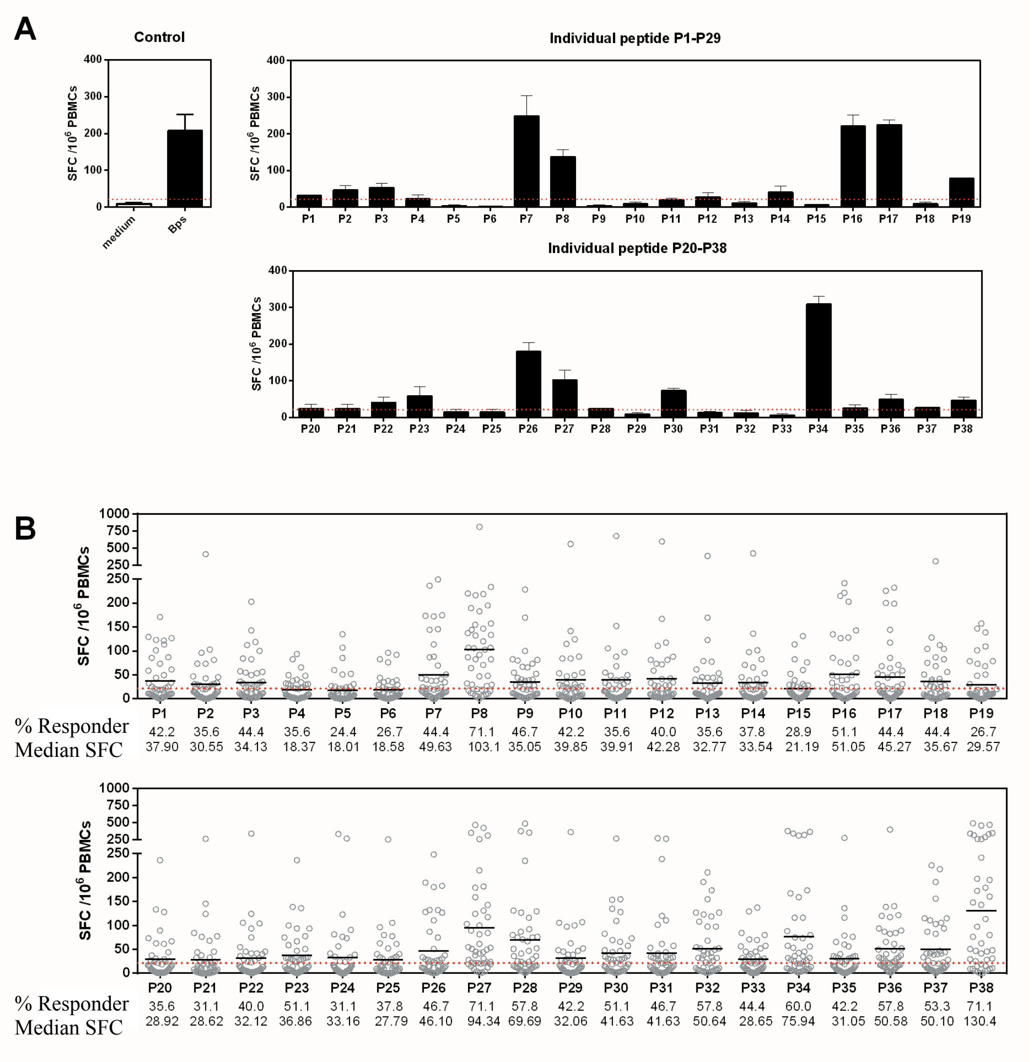

Figure S3: Strategy for data analysis. Example shown for BPSL3319 peptide library - T cell epitope mapping by ELISpot. A BPSL3319 peptide library was generated to cover the full-length amino acid sequence of BPSL3319 (38 peptides, P1-P38, using 20 mer peptides with a 10 amino acid overlap). The ELISpot result from one representative donor is shown in Panel A. Any donor with a response to peptide greater than the mean + 2 S.D. of the medium only control (red dotted line) was defined as a 'responder'. The distribution of IFNγ SFC/10^6 PBMCs of 45 donors to peptide is shown in Panel B. The results were analyzed as '% Responder' by calculating the number donors tested that had a positive response to an individual peptide divided by the total number of donors tested multiplied by 100. The 'Median SFC' is the horizontal line-median of SFC/10^6PBMC to individual peptide calculated from all donors).
